# Energy metabolism in health and diseases

**DOI:** 10.1038/s41392-025-02141-x

**Published:** 2025-02-18

**Authors:** Hui Liu, Shuo Wang, Jianhua Wang, Xin Guo, Yujing Song, Kun Fu, Zhenjie Gao, Danfeng Liu, Wei He, Lei-Lei Yang

**Affiliations:** Department of Stomatology, The First Affiliated Hospital of Zhengzhou University, Zhengzhou, China

**Keywords:** Drug development, Diagnostics

## Abstract

Energy metabolism is indispensable for sustaining physiological functions in living organisms and assumes a pivotal role across physiological and pathological conditions. This review provides an extensive overview of advancements in energy metabolism research, elucidating critical pathways such as glycolysis, oxidative phosphorylation, fatty acid metabolism, and amino acid metabolism, along with their intricate regulatory mechanisms. The homeostatic balance of these processes is crucial; however, in pathological states such as neurodegenerative diseases, autoimmune disorders, and cancer, extensive metabolic reprogramming occurs, resulting in impaired glucose metabolism and mitochondrial dysfunction, which accelerate disease progression. Recent investigations into key regulatory pathways, including mechanistic target of rapamycin, sirtuins, and adenosine monophosphate-activated protein kinase, have considerably deepened our understanding of metabolic dysregulation and opened new avenues for therapeutic innovation. Emerging technologies, such as fluorescent probes, nano-biomaterials, and metabolomic analyses, promise substantial improvements in diagnostic precision. This review critically examines recent advancements and ongoing challenges in metabolism research, emphasizing its potential for precision diagnostics and personalized therapeutic interventions. Future studies should prioritize unraveling the regulatory mechanisms of energy metabolism and the dynamics of intercellular energy interactions. Integrating cutting-edge gene-editing technologies and multi-omics approaches, the development of multi-target pharmaceuticals in synergy with existing therapies such as immunotherapy and dietary interventions could enhance therapeutic efficacy. Personalized metabolic analysis is indispensable for crafting tailored treatment protocols, ultimately providing more accurate medical solutions for patients. This review aims to deepen the understanding and improve the application of energy metabolism to drive innovative diagnostic and therapeutic strategies.

## Introduction

Energy metabolism, a cornerstone of physiological function, has been extensively scrutinized since von Helmholtz first articulated the concept in 1847.^1,2^ Over time, our comprehension of this vital process—which underpins life by supplying the essential energy required for diverse cellular activities—has expanded profoundly.^3–7^ Energy metabolism involves a series of sophisticated biochemical pathways that convert nutrients into adenosine triphosphate (ATP), the primary energy currency of cells. The meticulous regulation of these pathways is paramount for sustaining cellular homeostasis and ensuring the optimal functioning of organs and tissues. Dysregulation in energy metabolism is intricately associated with the pathogenesis of various disorders, encompassing neurological diseases, cardiovascular conditions, metabolic syndromes, autoimmune disorders, and cancer.^8–14^

Abnormal energy metabolism linked to the aforementioned diseases has been extensively studied.^15,16^ However, the mechanisms of intracellular energy conversion, dynamic changes in energy metabolic pathways, and the regulatory signals of different energy metabolic pathways remain unclear. Notably, the heterogeneous regulation of metabolism across different tissues and organ systems involving genetics, environment, and sex, among other factors, remains relatively under-studied. Recent studies are shifting focus towards intercellular energy transfer interactions, such as in pancreatic ductal adenocarcinoma, where lipid-rich cancer-associated fibroblasts transfer lipids to cancer cells, increasing oxidative phosphorylation (OXPHOS) to promote cancer cell growth.^17^ Nevertheless, owing to the complex interplay between immune and non-immune cells, mechanisms of energy interactions and regulatory strategies require further exploration. Mitochondria are central to energy metabolism; however, our understanding of mitochondrial dynamics, their role in regulating energy metabolism, and leveraging mitochondrial function to improve disease prognosis is still limited.

**Fig. 1:**
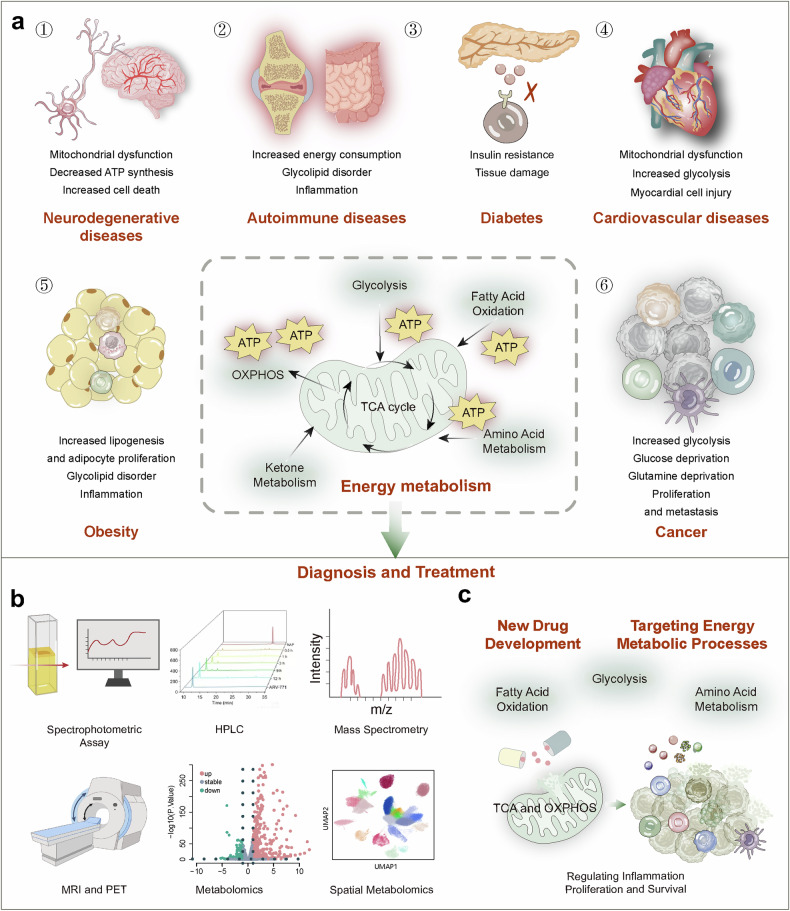
Diagram of energy metabolism alterations, detection, and therapeutics. **a** Energy metabolic alterations accompany a variety of diseases, which include increased energy demands and shifts in energy production pathways, ultimately leading to mitochondrial dysfunction-based metabolic disorders that cause functional abnormalities or cell death in normal cells. **b** Detection methods for altered energy metabolism encompass established spectroscopic assays, as well as advanced imaging techniques such as MRI and PET/CT, and the burgeoning field of metabolomics, including spatial omics technologies. **c** Pharmacological interventions targeting changes in energy metabolism are directed at multiple stages of metabolic pathways, including glycolysis, fatty acid oxidation and mitochondrial oxidation, to ameliorate abnormal energy metabolic shifts

Therefore, a deeper understanding and research on energy metabolism can contribute to the diagnosis and treatment of various diseases. With technological advancements, detecting energy metabolic processes has become more feasible. However, in complex physiological and pathological microenvironments, the challenge lies in non-invasively and reliably monitoring the heterogeneous energy metabolism across different cell types using imaging, mass spectrometry, and biosensors. Although multi-omics technologies are evolving, the integration of metabolomics, spatial transcriptomics, imaging, and clustered regularly interspaced short palindromic repeats screening techniques for interdisciplinary diagnosis of diseases presents a promising yet under-explored research direction. Current treatments for energy metabolic diseases mainly target key pathways; however, owing to dynamic changes in disease energy metabolism, where early-stage tumors rely on glycolysis and advanced stages on fatty acid oxidation (FAO), treatment efficacy is often sub-optimal. This ongoing controversy underscores the importance of some studies supporting dietary modifications (such as ketogenic diets) to adjust energy metabolism, while others emphasize the significance of drug therapies. Future exploration lies in integrating targeted energy metabolism pathways with diet and/or standard care therapies like immunotherapy. Deep insights into the dynamic changes of energy metabolism in health and disease aid in discovering early diagnostic and therapeutic metabolic biomarkers. Studies on targeted therapeutic drugs for metabolic pathways are limited. Thus, future studies should focus on identifying specific and stable metabolic biomarkers and developing multi-targeted therapeutic drugs (Fig. 1).

This comprehensive review explores the evolution and progress of energy metabolism research, providing an in-depth examination of its core pathways and regulatory mechanisms. We briefly summarize various energy metabolic pathways, including glycolysis, OXPHOS, FAO, and amino acid metabolism. A detailed analysis of the regulatory pathways is provided, encompassing hormone regulation, adenosine monophosphate-activated protein kinase and mechanistic target of rapamycin signaling, and the impact of the sirtuin (SIRT) protein family, emphasizing the roles of metabolic products or non-metabolic enzymatic functions in energy metabolism. Our analysis extends to the specific metabolic adaptations inherent in various pathophysiological states, including metabolic shifts in neurodegenerative diseases (ND), heart metabolic reprogramming in cardiovascular diseases, disturbances in metabolic pathways in obesity and diabetes syndromes, autoimmune diseases, and cancer, focusing on how disease treatment can be achieved through targeting energy metabolism. Throughout the discussion, we further analyzed the controversies and limitations of current research, identifying directions for future investigation. We introduced cutting-edge technologies such as fluorescent probes, chromatography, metabolomics, and nanobiomaterials, comparing the characteristics of these technologies, significantly enhancing the capacity to study and monitor metabolic processes. However, review articles may lack depth and detail in certain areas compared with research articles focused on a single theme, which can be a limitation. Ultimately, this review aims to provide a comprehensive understanding of the intricate patterns of energy metabolism, stimulate further exploration, and drive the development of innovative diagnostic and treatment strategies.

## Chronicles of energy metabolism research

**Fig. 2:**
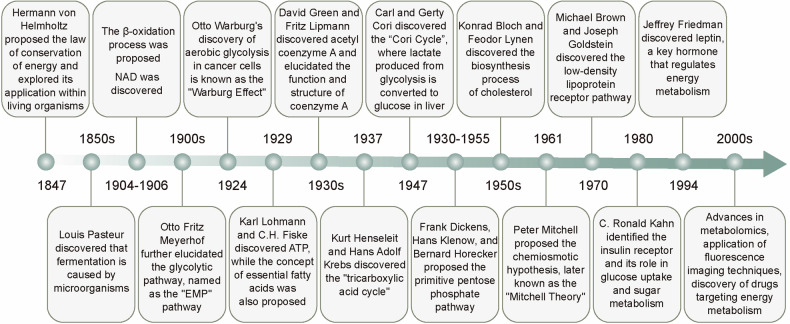
The milestones of energy metabolism development. The field of energy metabolism research originated in the mid-19th century and experienced rapid growth throughout the 20th century. Historical milestones, including the establishment of the law of energy conservation, the discovery of the Warburg effect, the identification of ATP, and the elucidation of the TCA cycle, have significantly advanced our understanding of energy metabolic processes and led to the revelation of multiple metabolic pathways. With the advent of the 21st century, researchers are extensively investigating the regulatory mechanisms of energy metabolism and actively exploring methods for its detection, as well as therapeutic strategies targeting energy metabolism for disease treatment

Energy metabolism encompasses a series of intricate and complex biochemical processes within organisms that involve the release, transfer, storage, and utilization of energy. Organisms must continually extract energy from food to sustain and promote growth. The metabolism of glucose, fats, and amino acids produces ATP, which is required for cellular energy processes. Notably, carbohydrate and lipid metabolism accounts for >90% of the energy requirements of the body. Among these metabolic pathways, aerobic oxidation plays a crucial role in ATP production, ensuring the efficient conversion and utilization of energy. This process is not only essential for maintaining fundamental biological functions but also plays a pivotal role in the onset, progression, and treatment of various diseases (Fig. 2).

In 1847, von Helmholtz introduced the concept of energy metabolism and further developed this theory by exploring the application of the “law of conservation of energy” within biological systems, building on the work of Mayer and others.^1^ Further, the discovery of nicotinamide adenine dinucleotide (NAD) in 1906 provided critical insights into the roles of enzymes in energy metabolism.^18,19^ In 1929, Lohmann and Fiske identified ATP as a pivotal energy molecule.^20,21^ Moreover, since its discovery, it has been recognized as a central molecule in energy metabolism, catalyzing extensive scientific investigations.^22^

Glucose metabolism is the primary energy-producing mechanism in the body, contributing approximately 50–70% of the total energy supply. For over a century, studies on glucose metabolism, with an early focus on glycolysis, have broadened our understanding of the mechanisms underlying cellular energy production. In the 1850s, Louis Pasteur first unveiled the process of microbial fermentation, providing a foundation for understanding how cells convert glucose into energy.^23^ In 1897, Buchner discovered that cell-free extracts could also perform fermentation.^24^ Building on previous studies, including the isolation of adenosine monophosphate (AMP) and description of the glycolytic pathway by Embden, Parnas’s studies on phosphorylative processes, and Lohmann’s discovery of ATP, Meyerhof characterized the glycolytic pathway and identified the enzymes involved. This pathway was subsequently named the “Embden–Meyerhof–Parnas pathway” and became recognized as the first metabolic pathway ever discovered.^25,26^ During the 1920s and 1930s, Warburg further elucidated the glycolysis process in cancer cells, in which glucose is broken down into lactic acid.^27–30^ He observed that cancer cells prefer glycolysis for energy production even under aerobic conditions, a phenomenon later termed the “Warburg effect”.^31–34^ This discovery has provided new avenues for cancer research, contributing crucial insights into the metabolic adaptations in cancer cells.

In 1931, Warburg was awarded the Nobel Prize in Physiology or Medicine for his discovery of the nature of and mechanism associated with respiratory enzymes, establishing the groundwork to understand electron transfer mechanisms involved in cellular respiration. He later conceptualized the respiratory chain, determining that NAD^+^ serves as an electron carrier, and uncovered the existence of nicotinamide adenine dinucleotide phosphate (NADP^+^). This provided the basis for OXPHOS, which involves the transfer of electrons through a series of protein complexes to ultimately produce ATP.^35,36^ In 1937, Krebs and Henseleit discovered the tricarboxylic acid (TCA) cycle, which converts pyruvate into carbon dioxide and water under aerobic conditions while generating energy.^37,38^ This discovery is regarded as a milestone in metabolic research and provides crucial insights into the mechanisms underlying energy production in cells under aerobic conditions. The TCA cycle is a central pathway for the complete oxidation of carbohydrates, fats, and proteins (amino acids) and represents a pivotal link between their inter-conversion and energy release.^39^ In 1961, Mitchell proposed a chemiosmotic hypothesis to explain the formation and utilization of a proton gradient during OXPHOS. This theory, later known as the Mitchell hypothesis, revolutionized the understanding of bioenergetics.^40^ Further, in 1957, Boyer, Walker, and Skou elucidated the roles of ATP synthase and ATPases in energy production, via glucose metabolism.^41^ Their work provided key insights into the molecular mechanisms that drive ATP synthesis, further advancing knowledge on cellular energy metabolism.

Other pathways and regulatory molecules involved in glucose metabolism were also identified. In 1947, Carl and Gerty Cori identified the Cori cycle, a mechanism by which lactate, produced *via* anaerobic glycolysis in the muscles, is converted back to glucose in the liver.^42^ Between 1930 and 1955, substantial contributions by Dickens, Klenow, and Horecker led to the identification and subsequent refinement of the pentose phosphate pathway.^43^ In cells, this pathway generates NADPH, which supports their antioxidant responses.^44^ In the 1980s, Kahn elucidated the role of insulin receptor in glucose uptake and metabolism, advancing the understanding of insulin signaling and resistance.^45–48^ Moreover, in 1994, Friedman discovered leptin, a hormone that regulates energy balance and metabolism.^49^ Since the 2000s, studies have revealed how alterations in glucose metabolism promote cancer cell growth, uncovering reprogrammed metabolic pathways in cancer cells and elucidating the role of glucose metabolism in tumor development.^50^ These findings have suggested new strategies for cancer therapy, offering potential avenues for targeting metabolic alterations in cancer cells.

Concurrently, with ongoing advancements in glucose metabolism research, fatty acid metabolism processes have also been progressively elucidated. Fatty acid metabolism contributes to approximately 30–50% of the energy requirements of the body. In the 1920s, Bloor and Burr conducted preliminary studies on the role of lipids in cellular functions and nutrition. In 1929, George and Mildred Burr discovered the dietary necessity of certain fatty acids, highlighting that specific fats not only provide energy to the body but also play a critical role in sustaining life. These essential fatty acids, including the well-known ω-3 and ω-6 fatty acids, cannot be synthesized by the body and must be obtained through the diet.^51–54^ In the 1930s, Green and Lipmann discovered ATP-dependent acetylating enzymes and elucidated the role and structure of coenzyme A, revealing its critical role in fatty acid metabolism. This discovery facilitated an understanding of the activation and entry of fatty acids into the β-oxidation process.^55–57^ Specifically, acetyl-CoA generated from β-oxidation enters the TCA cycle for ATP production. In the 1950s, Bloch and Lynen uncovered the complex process of cholesterol biosynthesis.^58,59^ Moreover, in 1971, Corey and Skoulos successfully synthesized prostaglandins, and during the 1970s, Brown and Goldstein discovered the low-density lipoprotein receptor pathway, which not only provides cells with the cholesterol necessary for constructing cell membranes and other biomolecules but also helps to maintain cholesterol homeostasis in the plasma.^60–62^ In the 1980s, the role of oxidized low-density lipoprotein in atherosclerosis was identified, enhancing the understanding of the involvement of fatty acid metabolism in cardiovascular disease.^63–65^ Since the 2000s, the development of metabolomics has enabled the comprehensive analysis of lipids within biological systems, advancing research on the metabolic pathways linking lipid metabolism to cancer progression. Amino acid metabolism primarily supports the resynthesis of cellular components or the synthesis of bioactive substances, such as enzymes and hormones. Under normal conditions, the role of amino acid metabolism in energy provisions is limited.

As research on energy metabolism broadens, its links to various diseases have progressively been revealed, alongside continuous advancements in therapeutic approaches for metabolic disorders. In the 1950s, the discovery of the Cori cycle revealed the biochemical pathway by which lactate is converted back to glucose in the liver, enhancing our understanding of glucose metabolism in conditions such as diabetes.^42,66^ In the 1980s, Kahn provided crucial insights into how insulin regulates glucose uptake and metabolism, thereby enhancing our understanding of insulin resistance and type 2 diabetes (T2DM).^45,48,67^ Concurrently, in-depth studies of fatty acid metabolism have highlighted the critical role of FAO in energy metabolism, offering essential clues to understanding the role of fats in metabolic diseases.^68,69^ In 1994, the discovery of leptin, a hormone that regulates energy balance and body weight, provided new possibilities for treating obesity and cancer.^70,71^ In the 1990s, researchers began to focus on the central role of mitochondria in energy metabolism, identifying mitochondrial dysfunction as a contributing factor to various metabolic diseases,^72,73^ thus providing a theoretical foundation for developing mitochondria-targeted therapies. Since the 2000s, studies have increasingly emphasized metabolic pathways in cancer cells, revealing how altered glucose and lipid metabolism support cancer cell growth and survival.^74,75^ Additionally, studies on glutamine metabolism in cancer cells have identified new therapeutic targets for cancer treatment, further elucidating the relationship between metabolism and cancer progression.^76^ For example, researchers can influence tumor cell growth and survival by modulating energy metabolism pathways, and clinical studies integrating metabolic inhibitors into cancer treatment protocols are transforming metabolic insights into therapeutic strategies. With advancements in genomics and metabolomics, the application of metabolomic technologies has enabled more accurate diagnosis and monitoring of metabolic diseases, such as cancer, diabetes, and obesity.^77,78^ Recent studies have also demonstrated, for the first time, that alterations in cardiac energy metabolism can promote heart regeneration. By inhibiting FAO, cardiomyocyte energy metabolism shifts from FAO to glycolysis, thereby triggering the regenerative capacity of heart cells.^79^

## Energy metabolism in physiology

### Overview of energy metabolism

**Fig. 3:**
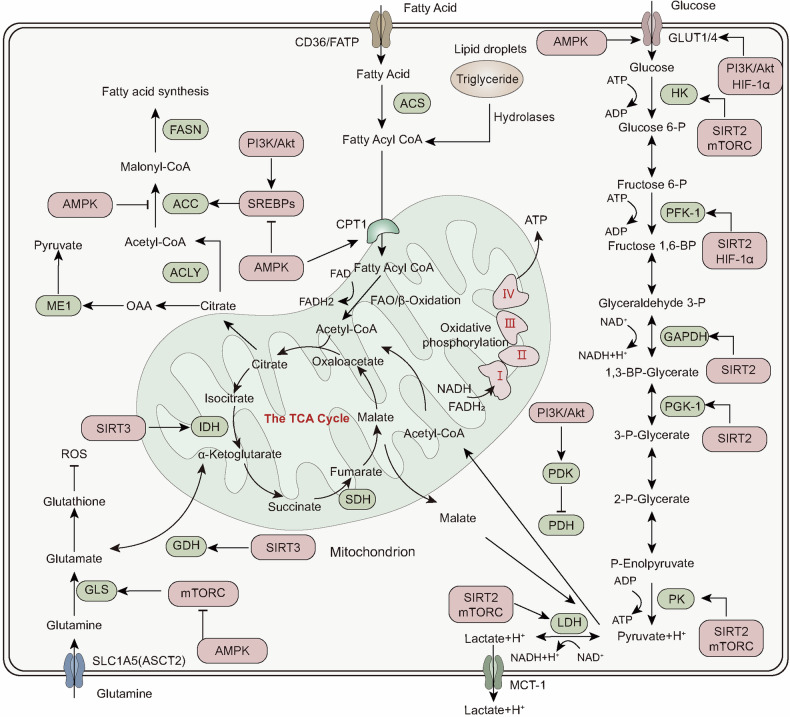
Main pathways for cellular energy production and molecular signal regulation. Glycolysis begins with the phosphorylation of glucose by hexokinase (HK), producing 6-phosphogluconate. Subsequently, phosphofructokinase-1 (PFK-1) converts 6-phosphofructose into 1,3-bisphosphoglycerate. In the subsequent cleavage reaction, aldolase (ALDO) cleaves 1,3-bisphosphoglycerate into two molecules of 3-phosphoglyceraldehyde. 3-phosphoglyceraldehyde is oxidized to 1,3-bisphosphoglycerate under the catalysis of glyceraldehyde-3-phosphate dehydrogenase (GAPDH), and reduced coenzyme II (NADH) is produced in the process. Thereafter, 1,3-bisphosphoglycerate is converted into 3-phosphoglycerate by phosphoglycerate kinase (PGK1), generating one molecule of ATP. 3-phosphoglycerate is then transformed into 2-phosphoglycerate by phosphoglycerate mutase (PGAM1), and then catalyzed by enolase (ENO1) to form 2-phosphoenolpyruvate. 2-phosphoenolpyruvate is ultimately converted into pyruvate under the action of pyruvate kinase (PK), releasing another molecule of ATP. Under anaerobic conditions, pyruvate is reduced to lactate by lactate dehydrogenase (LDH). Fatty acids first need to be activated into acyl-CoA (acyl-coenzyme A). After activation, the fatty acids are transferred from the cytoplasm to the mitochondrial matrix through Carnitine palmitoyltransferase I (CPT1). The fatty acids undergo a series of β-oxidation cycles, resulting in the production of acetyl-CoA and NADH. Glutamine enters the cell through ASCT2/SLC1A5 and is converted into glutamate by the deamination reaction catalyzed by glutaminase (GLS). Glutamate can be further converted into alpha-ketoglutarate (α-KG). Nutrient-derived acetyl-CoA enters the TCA cycle, which is catalyzed by enzymes such as succinate dehydrogenase (SDH), fumarate hydratase (FH), and isocitrate dehydrogenase (IDH), ultimately producing energy molecules ATP and reducing agents NADH and FADH2. NADH and FADH2 enter OXPHOS to further generate ATP. In the aforementioned process, various signaling molecules, such as AMP-activated protein kinase (AMPK), phosphatidylinositol-3-kinase/protein kinase B (PI3K/AKT), mechanistic target of rapamycin complex (mTORC), and sirtuins (SIRT), play crucial regulatory roles in controlling energy production under physiological conditions. PDH, pyruvate dehydrogenase; PDK, pyruvate dehydrogenase kinase; GDH, glutamate dehydrogenase; ACS, acyl-CoA synthetase; ACC, acetyl-CoA carboxylase; FASN, fatty acid synthase; ACLY, ATP citrate lyase

Living organisms rely on energy to support growth and reproduction, maintain structural integrity, and respond to environmental changes. Energy metabolism encompasses the intricate biochemical processes responsible for extracting energy from food sources and utilizing it for various physiological activities. Associated pathways consist of interconnected processes, such as glycolysis, the citric acid cycle (Krebs cycle), and OXPHOS, which are tightly regulated by enzymes and metabolic intermediates. The primary objective of energy metabolism is ATP generation, which is considered the cellular currency for energy transactions. ATP plays a critical role in essential functions, including muscle contraction, neuronal signaling, and metabolic synthesis. The subsequent sections provide a comprehensive overview of the different aspects of energy metabolism, including the fundamental steps of various energy metabolism types and the hormonal and signaling systems governing its regulation (Fig. 3).

#### Glycolysis

Glycolysis was the first metabolic route discovered, and the term “glycolysis” derives from the Greek word “glykys,” meaning sweetness, and “lysis,” meaning division or splitting.^80^ This refers to the breakdown of a single glucose molecule into two pyruvate molecules that act as glycolytic end products. Under aerobic conditions, pyruvate usually enters the mitochondria for oxidation to generate acetyl-CoA. Conversely, under anaerobic conditions, pyruvate is reduced to lactate. There are three essential components associated with glycolysis. First, it serves as the primary pathway for ATP generation when oxygen is limited or in cells without mitochondria, such as red blood cells. Second, under conditions of abundant oxygen, glycolysis produces pyruvate, which subsequently enters the mitochondrial TCA cycle to generate ATP. Third, glycolysis and TCA cycle yield various metabolites that can participate in anabolic pathways for NADPH synthesis and the generation of vital constituents.^80^

#### TCA cycle

In 1937, an important study titled “The Significance of Citric Acid in Animal Tissue Intermediate Metabolism” initially presented the notion of the TCA cycle, which was alternatively recognized as the Krebs cycle.^81^ The TCA cycle plays a vital role in eukaryotic cell metabolism by facilitating the entry of various molecules, such as fatty acids, amino acids, and pyruvate, into the cycle. Unlike linear pathways, the TCA cycle operates in a cyclic manner, with oxaloacetate serving as both the initial material for citrate synthesis, catalyzed by citrate synthase and the final product, produced by malate dehydrogenase, ensuring continuous renewal of the cycle. Notably, this pathway is considered amphibolic because it provides intermediates for macromolecule synthesis (such as lipids) and generates NADH and reduced flavin adenine dinucleotide (FADH_2_) molecules, which are necessary for ATP production *via* OXPHOS. Owing to its ability to accommodate multiple substrates, the TCA cycle plays a central role in cellular metabolism.

#### OXPHOS

OXPHOS is an essential process for ATP production within cells, particularly under aerobic conditions as part of cellular respiration. The key to OXPHOS lies in the electron transport chain (ETC), composed of a series of protein complexes that accept electrons from NADH and FADH2 and pass them to oxygen, the final electron acceptor, which combines with protons to form water. The energy released during this electron transport is converted into chemical energy, which leads to the combination of ADP and inorganic phosphate (Pi) to form ATP. This process is central to cellular energy metabolism and is crucial for maintaining the life activities of cells and organisms.

#### Glutamine metabolism

Glutamine plays a crucial role as the primary source of energy for rapidly dividing cells, including hematopoietic stem cells and cancer cells. Glutamine is taken up by cells through specific transporters, such as SLC1A5, SLC38A1, and SLC38A2. Once inside the cell, it is used for various biosynthetic processes in the cytoplasm, including hexosamine production, nucleotide synthesis, and asparagine formation.^82^ Glutaminase (GLS) converts glutamine to glutamate by catalyzing its hydrolysis and releasing ammonium ions. The resulting mitochondrial glutamate can exit the mitochondria into the cytosol, where this exported glutamate plays a role in the synthesis of important molecules, such as glutathione and non-essential amino acids (NEAAs). Glutamate within mitochondria is further converted into α-ketoglutarate (α-KG). α-KG participates in fatty acid biosynthesis and NADH generation.^83^ It also serves as a substrate for both OXPHOS pathways, supporting the TCA cycle.^84^ Within the OXPHOS pathway, metabolites derived from glutamine contribute to the generation of electron donors, such as NADH or FADH2, which are utilized for ATP synthesis through the ETC.

#### FAO

FAO is one of the important pathways for cells to obtain energy, especially during prolonged fasting, starvation, or intense exercise when glycogen stores are depleted. The FAO process begins with acyl-CoA dehydrogenase. This enzyme forms a trans double bond between the alpha and beta carbons on acyl-CoA, yielding FADH2, subsequently contributing 1.5 ATP molecules *via* the ETC.^85^ The next step involves enoyl-CoA hydratase, which hydrates the double bond through the addition of a hydroxyl group to the beta carbon and a proton to the alpha carbon. In the third step, β-hydroxyacyl CoA dehydrogenase oxidizes the beta carbon, producing NADH, which generates 2.5 ATP molecules through the ETC.^86^ In the final step, catalyzed by β-keto thiolase, the α-β carbon bond is broken, resulting in the formation of acetyl-CoA and a shortened fatty acyl-CoA, allowing the cycle to repeat until all even-chain fatty acids are converted into acetyl-CoA.

#### Ketone metabolism

Ketones, including β-hydroxybutyrate (BHB), acetoacetate (AcAc), and acetone, are synthesized primarily from fatty acids by the liver.^87^ Catalyzed by β-ketoacyl-CoA synthase (HMGCS2), two acetyl-CoA molecules combine to form HMG-CoA, which is then broken down into AcAc and acetyl-CoA by HMG-CoA lyase (HMGCL). Most AcAc is then reduced back to BHB by β-hydroxybutyrate dehydrogenase (BDH1). Ketones are transported to target organs, metabolized in the mitochondria, and converted back to acetyl-CoA for energy generation. Ketone production increases mainly when the supply of glucose/acetoacetate decreases to maintain energy production. However, ketone production also leads to an increase in mitochondrial oxidative stress, a mechanism significantly incongruent with the anti-inflammatory effects of ketone supplementation.^88^ Ketone supplementation is generally believed to initially promote inflammation but gradually shifts toward anti-inflammatory and antioxidative mechanisms as cells adapt. This involves the regulation of NRF2, SIRT, and AMPK.^89^

The relationship between ketone metabolism and pathophysiology is closely intertwined. In the nervous system, ketones can enter brain tissue through monocarboxylic acid transporters (MCTS) in endothelial and astrocytic cells. Neurons, astrocytes, and oligodendrocytes metabolize ketones at a higher rate than glucose dose, providing energy. Ketones protect neurons by improving mitochondrial respiration and reducing inflammation.^90^ In the cardiovascular system, ketones serve as an energy source for cardiac muscle and endothelial cells. While they do not enhance cardiac efficiency,^91^ they can improve cardiac inflammatory conditions by inhibiting the NLRP3 signal.^92^ In cardiovascular diseases, ketones provide a potential alternative fuel source for a failing heart.^93^ However, the role of ketone oxidation in myocardial infarction remains unclear. In cancer, ketones exhibit diverse effects, either promoting or inhibiting cancer cell proliferation. Studies suggest that ketones are essential for CD8^+^ T cells as an energy source, enhancing tumor killing effects and strengthening effector functions.^94^ However, other research indicates that ketones may support tumor growth and metastasis as energy sources. In pancreatic ductal adenocarcinoma, ketone metabolism, particularly BHB as an energy source, promotes tumor growth and progression.^95^ Further research is needed to understand how ketones regulate cancer progression.

### Non-metabolic functions of energy metabolism enzymes and metabolites

During energy metabolism, several metabolic enzymes and metabolites play crucial non-metabolic roles, referred to as “moonlighting” functions, to regulate gene transcription, translation, and epigenetic modifications. These functions exert considerable impacts under various physiological and pathological conditions.

#### Regulation of gene expression

Metabolic enzymes influence gene expression through diverse mechanisms. For instance, metabolic enzymes localized in the cytoplasm or mitochondria can translocate to the nucleus and modify chromatin structure by interacting with histones and DNA, thereby directly regulating gene expression. HK2 interacts with nuclear proteins such as Max, Sirt1, IWS1, CTR9, and Spin1, increasing chromatin accessibility to regulate gene expression.^96^ Upon activation, phosphofructokinase 1 (PFK1) can bind to the transcription factor TEAD, stabilizing its interaction with YAP/TAZ in the nucleus, promoting YAP/TAZ transcriptional output, and impacting breast cancer progression.^97^ In glioblastoma cells with aberrant epidermal growth factor receptor (EGFR) signaling, PFK1 undergoes acetylation at the K395 site and translocates to the plasma membrane, recruiting and binding with p85α, leading to sequential activation of PI3K/AKT, PFK2, and PFK1.^98^ Moreover, pyruvate kinase M2 (PKM2) can enter mitochondria to maintain its function and translocate to the nucleus to regulate gene expression.^99^ Once in the nucleus, it can activate various transcription factors such as hypoxia-inducible factor 1 alpha (HIF-1α), histone H3, NRF2, and STAT3, influencing the activation of downstream target genes. Fructose-1,6-bisphosphatase inhibits the activity of HIF-1α in the nucleus, decreases the expression of HIF target genes, and promotes its degradation by binding to Notch1, regulating tumor formation.^100^ Nuclear translocation of fructose-1,6-bisphosphatase 2 inhibits c-Myc-mediated gene expression, thereby suppressing mitochondrial biogenesis and respiration.^101^ Phosphorylation of fumarate hydratase (FH) by p38 in response to TGF-β signaling leads to its binding to the transcription factor CSL/p53 complex on the p21 promoter, which inhibits histone H3K36 demethylation, enhances p21 transcription, and induces cell growth arrest.^102^ Phosphoenolpyruvate carboxykinase 1 utilizes GTP as a phosphate donor to phosphorylate INSIG1 and INSIG2 in the endoplasmic reticulum (ER), leading to the transcription of downstream lipid synthesis genes mediated by SREBP.^103^ NADPH directly inhibits the activation of HDAC3 through interaction, disrupting the binding of HDAC3 with its coactivating factors NCOR1/2, thereby regulating cellular epigenetics.^104^ Recent studies revealed that in macrophages, the mediator binds to 2-ketoacid dehydrogenases, generating acetyl-CoA, which increases histone acetylation levels in specific chromatin regions, thereby regulating gene transcription. However, the exact molecular mechanism of this binding requires further investigation.^105^

#### DNA repair

HK2 exhibits moonlighting functions in the nucleus, with its overexpression increasing chromatin accessibility at DNA repair sites, thereby reducing DNA double-strand breaks.^96^ Tumor-inducing EGFR signaling induces the phosphorylation of phosphoglycerate kinase 1 (PGK1) at S256 by casein kinase 2α (CK2α), leading to the binding of phosphorylated PGK1 to CDC7, converting local adenosine diphosphate produced by CDC7 into ATP, facilitating the recruitment of DNA helicase to the replication origin, and promoting DNA replication.^106^ Nuclear ATP-citrate lyase can be phosphorylated and activated in response to DNA damage, providing acetyl-CoA to promote the recruitment of BRCA1 and DNA repair.^107^ Under DNA damage stress, activation of tyrosine kinase SRC leads to phosphorylation of glyceraldehyde-3-phosphate dehydrogenase (GAPDH), crucial for its nuclear translocation. Nuclear GAPDH is recruited to DNA damage sites and interacts with DNA polymerase (PAR), participating in DNA repair mechanisms.^108^ Fumarate is involved in DNA damage response; it translocates to the nucleus after DNA damage, produces fumarate esters that inhibit histone lysine demethylase KDM2B, and promotes protein binding during DNA repair.^109^ Additionally, metabolites such as 2-hydroxyglutarate, succinate, and fumarate derived from mutations in IDH1/2, FH, and SDH inhibit DNA repair pathways by repressing lysine demethylase KDM4B, resulting in abnormal excessive H3K9 methylation at DNA break sites, impairing recruitment of TIP60 and ATM, reducing end resection, and decreasing recruitment of downstream repair factors.^110^

#### Post-translational protein modifications

Post-translational modifications of proteins, including acetylation, phosphorylation, and lactylation, are extensively influenced by metabolic processes. Acetyl-CoA serves as a primary driver for histone acetylation, a process facilitated by lactate dehydrogenase A (LDHA). In T cells lacking LDHA, histone acetylation is reduced at H3K9AC.^111^ Under oxidative stress, LDHA undergoes a transition from a tetramer to a dimer, facilitating its translocation to the nucleus. The atypical enzymatic activity of nuclear LDHA catalyzes the conversion of α-KB to α- hydroxybutyrate (α-HB), leading to α-HB accumulation. This accumulation, through enhanced histone methylation modifications, regulates gene expression, enhances cell resistance to reactive oxygen species (ROS), and promotes cell survival.^112^ Lactate accumulation influences protein transcription or physicochemical characteristics through lactylation. Lactylation of lysine 62 (K62) on PKM2 plays a role in regulating the feedback signaling of glycolysis.^113^ Lactylation also modulates immune cell functions; for instance, lactylation of MOESIN at Lys72 enhances its interaction with TGF-β receptor I, activating the TGF-β-SMAD3 signaling pathway and regulating Treg cell functions.^114^ Lactate-fueled histone lactylation facilitates the activation of repair genes in macrophages; however, reduced histone lactylation weakens gene expression in repair macrophages.^115^

In addition to affecting lactylation, lactate also impacts acetylation. Lactate inhibits the deacetylase SIRT1 through Hippo/YAP-mediated lactylation and G protein-coupled receptor 81-dependent β-arrestin2-mediated recruitment of p300/CBP in the nucleus, driving HMGB1 acetylation.^116^ Lactate also inhibits histone deacetylase activity, leading to excessive H3K27 acetylation at Tcf7 super-enhancer sites, increasing Tcf7 expression. This results in an increased proportion of CD8 + T cells expressing stem cell-like Tcf-1 in tumors, enhancing the efficacy of immunotherapy.^117^ Succinate indirectly influences the expression of histone deacetylases by stabilizing HIF-1α.^118^ Accumulation of fumarate inhibits the KDM5 family of histone demethylases, increasing the levels of the active gene transcription marker H3K4me3.^119^ Furthermore, fumarate accumulation can activate the PI3K/AKT signaling pathway through PTEN succinylation, exerting oncogenic effects.^120^ Acetylation of PKM2 at the K433 site by acetyltransferase p300 leads to the accumulation of the dimeric form of PKM2 in the nucleus, functioning as a protein kinase, phosphorylating STAT3 and activating downstream signaling pathways.^121^ However, the debate over whether PKM2 exhibits protein kinase activity continues. PGK1 can undergo auto-phosphorylation at the Y324 site to achieve maximal activation. Conversely, PTEN dephosphorylates self-phosphorylated PGK1, inhibiting its activity and thereby leading to a reduction in glycolysis in brain tumors.^122^

Extensive research on post-translational protein modifications has led to the discovery of new modification modes, such as histone tyrosine sulfation. SULT1B1 catalyzes the sulfation modification of histone H3 at the Y99 site directly using PAPS as a substrate, thereby regulating H4R3me2a and gene transcription.^123^ However, the specific sites and types of protein residue modifications remain partially defined, and the exact roles of protein residue modifications remain unclear. While protein modifications are reportedly involved in the development of many diseases, their precise roles and molecular mechanisms in different biological processes need further elucidation. A deeper understanding of the “writers,” “erasers,” and “readers” of these modifications on proteins, as well as their mechanisms of action in various biological processes, remains a focal point and challenge in current research.

### Signaling pathways that regulate energy metabolism

To maintain balanced energy intake and expenditure, which are crucial for the overall well-being and health of an organism,^124,125^ energy metabolism must be regulated. The absorption and utilization of nutrients, as well as the release of stored energy from fuel sources, are significantly influenced by signaling molecules and pathways.

#### Hormonal regulation of energy metabolism

Hormones are critical in energy metabolism, acting as messengers produced by various organs to regulate physiological processes. Key hormones like insulin, glucagon, leptin, and ghrelin influence metabolism by binding to specific receptors on target cells. Insulin, crucial during feeding for energy balance, modulates glucose metabolism and overall homeostasis. Its effects include (i) suppressing liver glucose production; (ii) enhancing glucose uptake in muscles, liver, and fat cells; (iii) inhibiting lipolysis, decreasing plasma fatty acids, and reducing liver glucose output; and (iv) promoting vasodilation in muscles to increase glucose disposal.^126^

Insulin’s role is mediated through signaling pathways associated with its receptor.^126^ The PI3K/Akt pathway is pivotal, where insulin activation phosphorylates phosphatidylinositol 4,5-bisphosphate *via* PI3K to produce phosphatidylinositol 3,4,5-triphosphate, triggering Akt.^127^ Akt also hinders ATP-citrate lyase activity, impedes fatty acid synthesis, and disrupts mammalian target of rapamycin complex 1 (mTORC1) function, thereby enhancing protein synthesis. Additionally, it activates sterol regulatory element binding proteins (SREBPs), which mediate the transcription of genes related to cholesterol and fatty acid synthesis. The PI3K/Akt pathway also regulates the translocation of glucose transporter 4 (GLUT4) from intracellular vesicles to muscle and fat cell membranes, facilitating glucose uptake upon insulin stimulation.^128^ Any interference in the sequential steps involved in GLUT4 transport could cause diabetes mellitus and insulin resistance.

In addition to insulin, pancreatic islets release glucagon, somatostatin, and other pancreatic polypeptides hormones, which collaborate to maintain ideal glucose levels in the bloodstream and control energy metabolism.^129^ Insulin secretion is stimulated by glucagon, whereas somatostatin acts as an inhibitor.^129^ Consequently, β-cells integrate multiple regulatory inputs to ensure adequate insulin secretion and maintain glucose homeostasis.^129^ Additionally, the brain is affected by the hormone leptin, which is released by fat cells and regulates overall energy balance by reducing food intake and increasing energy utilization.^130^ Leptin signaling involves a receptor located on the cell membrane that initiates downstream signaling pathways, such as the Janus kinase (JAK)/signal transducer and activator of transcription (STAT) pathway, to stimulate energy expenditure while suppressing appetite.^131^

#### AMPK signaling and energy metabolism

AMPK, which comprises an enzyme complex, is critical for controlling cellular metabolism and maintaining energy equilibrium.^132^ Moreover, it functions in the detection of the cellular energy level and adjusts to variations in baseline conditions, stressors, and pathological conditions.^132^ In mammals, the energy status is sensed by AMPK through its ability to monitor cellular AMP, ADP, and ATP levels.^133^ AMPK is activated when ATP levels decrease and AMP levels increase,^133^ and its activation aids in restoring energy equilibrium by inhibiting ATP-consuming processes and promoting ATP-generating processes.^133^ Furthermore, by regulating cellular metabolic functions, AMPK can prime organs and tissues to defend against ischemic damage and promote the prompt resolution of inflammatory responses.

AMPK regulation is pivotal in responding to energy stress by sensing changes in intracellular AMP, ADP, and ATP levels.^134^ AMPK activation occurs through a three-step mechanism: first, AMP or ADP binds to the γ subunit, leading to Thr172 phosphorylation in the α subunit’s kinase domain via upstream kinases.^134^ Liver-kinase-B1 (LKB1), a serine/threonine kinase, is the main kinase for Thr172 phosphorylation during energy stress,^135–137^ a critical step that increases AMPK activity 100-fold in vitro.^138–140^ Second, AMP or ADP binding causes a structural change that protects Thr172 from dephosphorylation, although the specific phosphatases involved under physiological conditions are largely unidentified, with some suggested by recent studies.^141,142^ Lastly, AMP (but not ADP) significantly boosts allosteric activity, increasing it potentially by 10-fold.^138^ Notably, ATP inhibits all three mechanisms. In addition to variations in adenine nucleotide levels, there are alternative and noteworthy mechanisms that control AMPK. One extensively investigated strategy for regulating AMPK, which does not rely on nucleotides, involves Thr172 phosphorylation mediated by calcium/calmodulin-dependent protein kinase kinase 2 (CAMKK2).^143–145^ CAMKK2 is triggered by elevations in intracellular Ca^2+^ level. Therefore, although CAMKK2 does not directly sense the cellular energy status, it plays a crucial role in regulating various aspects of overall metabolism through AMPK.

Beyond the traditional AMP/ADP-dependent mechanisms, AMPK can be activated through alternative pathways.^146,147^ The glycolytic process involves the conversion of glucose into fructose-1,6-bisphosphate (FBP), a molecule that is subsequently metabolized by FBP aldolases. Research indicates that glucose deprivation leads to a decrease in FBP-bound aldolase, which in turn initiates the phosphorylation and subsequent activation of LKB1, a key upstream kinase of AMPK. Consequently, this study reveals the pivotal role of FBP as a metabolic signal for glucose levels and identifies FBP aldolases as metabolic sensors that communicate the status of glucose availability to AMPK.^146,147^

In recent years, our knowledge of how AMPK regulates metabolism has advanced significantly, owing to the discovery of numerous substrates that are targeted by this kinase. This progress was greatly facilitated by the identification of a specific motif recognized by AMPK.^148,149^ Additionally, innovative proteomic techniques have expanded the list and range of substances that are potentially regulated by AMPK.^150–152^ As previously mentioned, AMPK plays a crucial role in replenishing ATP levels under metabolic stress by temporarily suppressing ATP utilization in biosynthetic processes, while simultaneously activating pathways that facilitate ATP production. AMPK phosphorylates various transcription factors (or cofactors) that serve as key regulators of biosynthetic pathways and metabolism.^153^ In this manner, AMPK can promptly reinstate energy equilibrium while transcriptionally reprogramming cellular metabolism in response to extended periods of reduced energy levels.

##### Glucose and lipid metabolism

Glucose and lipids play crucial roles in providing and storing energy within cells. Through separate mechanisms, AMPK stimulates glucose uptake by phosphorylating TBC domain family member 1 (TBC1D1) and thioredoxin-interacting protein (TXNIP), which regulate the movement and surface expression of GLUT4 and GLUT1.^154,155^ AMPK also plays a role in the acute regulation of glycolysis in certain tissues through the phosphorylation of PFKFB3. Additionally, it inhibits glucose storage in specific tissues by targeting and inhibiting multiple GYS isoforms involved in glycogen synthesis.^154,156^ It also regulates cellular lipid metabolism by directly phosphorylating acetyl-CoA carboxylase (ACC)1 and ACC2, thereby inhibiting fatty acid synthesis and promoting FAO. This is achieved by alleviating the inhibition of carnitine palmitoyltransferase 1 (CPT1), which is mediated by the local production of malonyl-CoA at the outer membrane of the mitochondria, *via* ACC2. Notably, the amino terminus of ACC2 contains a sequence that targets it to the mitochondria. Additionally, 3-hydroxy-3-methyl-glutaryl-CoA (HMG-CoA) reductase (HMGCR) is phosphorylated and inhibited by AMPK. These combined effects on ACC1, ACC2, and HMGCR result in the preprogramming of lipid and sterol synthesis within the cell. AMPK was initially discovered for its ability to suppress fatty acid and cholesterol synthesis. This is achieved through direct phosphorylation, leading to the inhibition of ACC and HMG-CoA reductase. Furthermore, AMPK enhances food intake and reduces liver steatosis, fibrosis, insulin resistance, platelet dysfunction, renal fibrosis, and hepatocellular carcinoma. Moreover, the promotion of FAO in response to pharmacological activators also relies on the phosphorylation and inhibition of ACC, whereas alternative mechanisms, such as AKAP1 phosphorylation, might play a significant role during exercise. AMPK activity hinders the activation of gluconeogenesis-related genes by phosphorylating cyclic-AMP-regulated transcriptional co-activator 2 (CRTC2) and histone deacetylases (HDACs), which are essential cofactors for gene transcription and are involved in de novo glucose synthesis.^157,158^ In addition, AMPK phosphorylates and inhibits transcription factors that stimulate glycolytic and lipogenic transcriptional processes, particularly SREBP1, a key regulator of lipid synthesis,^159^ as well as hepatocyte nuclear factor-4α (HNF4α) and carbohydrate-responsive element binding protein (ChREBP).^160,161^ Hence, the immediate stimulation of AMPK facilitates the absorption of glucose to facilitate ATP replenishment, whereas its prolonged activation alters cellular functions to restrict glucose and lipid synthesis while promoting the utilization of fatty acids for energy production.

##### Protein metabolism

The suppression of protein synthesis, mediated by AMPK, is primarily achieved through direct inhibition of mTORC1. Mammalian TOR serves as a central regulator that integrates signals from nutrients and growth factors, triggering various biosynthetic pathways, particularly the protein translation process, which promotes cellular expansion. Moreover, AMPK and mTORC1 play opposing roles in controlling cellular metabolism, and the activity of mTORC1 is suppressed by AMPK through a dual mechanism involving the phosphorylation-induced activation of tuberous sclerosis complex 2 (TSC2)^162^ and the phosphorylation-mediated inhibition of regulatory-associated protein of mTOR (Raptor), a component of the mTORC1 complex.^148^ In addition to inhibiting mTOR, AMPK limits protein synthesis by hindering ribosomal RNA production. This is accomplished through the phosphorylation and inhibition of TIF-IA, which is responsible for initiating RNA polymerase I activity.^163^ AMPK impedes protein elongation by activating eEF2K, an enzyme that suppresses the elongation process.^164^ Crucially, mTORC1 also acts as a primary regulator of eEF2K,^165^ exemplified by the numerous downstream targets of AMPK that are directly phosphorylated by mTORC1 or S6K1, to counteractively modulate their functions in relation to AMPK phosphorylation. Thus, AMPK and mTOR govern anabolism and catabolism by traversing the cellular environment and activating or deactivating a limited set of pivotal metabolic switches.

##### Autophagy

Autophagy is a cellular process involving the breakdown of proteins, organelles, and other large molecules *via* lysosomal transport. When energy availability is low, cells utilize this mechanism for regular turnover and to generate nutrients. AMPK plays a crucial role in promoting autophagy through various mechanisms. One such mechanism involves the phosphorylation and activation of unc-51-like autophagy-activating kinase 1 (ULK1) by AMPK, which initiates a cascade that triggers autophagy.^166–168^ Moreover, mTOR effectively inhibits autophagy by directly phosphorylating and suppressing ULK1.^167^ Consequently, AMPK promotes autophagy not only through direct ULK1 activation but also by inhibiting mTORC1 and preventing its suppressive effect on ULK1. Furthermore, ULK1 comprises another critical juncture where AMPK and mTOR regulate specific metabolic processes in contrasting manners. AMPK also differentially regulates VPS34-containing complexes that contribute to the initiation of autophagy.^169^ These complexes are essential for autophagosome formation and initiation. In addition, AMPK directly inhibits VPS34 by phosphorylating non-autophagic complexes lacking autophagic adaptor proteins. Simultaneously, it enhances VPS34 activity through the direct phosphorylation of beclin-1 in pro-autophagic complexes.^169^ Thus, AMPK is thought to hinder the unnecessary movement of vesicles and instead direct membrane trafficking towards the autophagic pathway in the absence of nutrients. However, many unanswered questions remain regarding the precise regulation and coordination of autophagy initiation in response to different stressors, as both AMPK and ULK1 can directly phosphorylate distinct regions within beclin-1 and Vps34. Additionally, both AMPK and ULK1 can phosphorylate Atg9, a transmembrane protein involved in the early formation of autophagosomes, thereby exerting control over its localization.^168,170,171^

#### mTOR signaling and energy metabolism

Mammalian TOR is widely recognized as a critical regulator of homeostasis, particularly in maintaining energy balance and mTORC1 plays a pivotal role in balancing growth-promoting factors with nutrient availability.^172^ At the cellular level, mTORC1 functions as a nutrient sensor, coordinating the equilibrium between anabolism and catabolism in response to external conditions.^173^ In mammals, changes in energy levels are closely tied to dietary intake. Under restricted feeding conditions, mTORC1 is activated to promote tissue growth and energy storage in organs, such as the liver and muscles. Conversely, during fasting, mTORC1 activity is suppressed to conserve resources.^173^ Activation of mTORC1 enhances metabolic pathways, including glycolysis, the oxidative branch of the pentose phosphate pathway, and de novo lipid biosynthesis.^174^

While mTORC2 signaling is less understood than mTORC1 signaling is, recent research has indicated that mTORC2 is involved in cellular metabolism and the organization of the actin cytoskeleton. It also enhances cell viability by activating the survival kinase Akt.^175,176^ Mammalian TORC1 is implicated in regulating the size, morphology, and synaptic plasticity of neurons and maintaining energy balance within the central nervous system.^177^ Additionally, mTORC1 is sensitive to intrinsic and extrinsic factors that inhibit cell growth, such as reduced ATP levels, oxygen deprivation, and genetic damage.^173^ A decrease in cellular energy, such as inadequate glucose supply, triggers the activation of AMPK, a metabolic regulator involved in the stress response, leading to the inhibition of mTORC1.^178^

##### Molecular structure of mTOR

The serine-threonine kinase mTOR, a member of the PI3K-related kinase (PIKK) family, is evolutionarily conserved. It forms two distinct protein complexes, mTORC1 and mTORC2, each with unique roles, regulatory mechanisms, and rapamycin sensitivity.^174^ Thus, mTORC1 consists of mTOR, Raptor, GβL/mLST8, DEPTOR, and PRAS40,^174^ whereas mTORC2 includes mTOR, GβL/mLST8, Rictor, Protor/PRR5, DEPTOR, and mSIN1. The primary role of mTORC1 is to integrate signals from growth factors and nutrients to drive cellular growth, under conditions of energy abundance or nutrient-scarcity-triggered catabolism.^174^ While mTORC1 is well-recognized for its role in cellular growth and metabolism, mTORC2 is more associated with the regulation of cell proliferation and survival.^173^

##### mTOR as an energy sensor

The mTORC1 pathway detects and regulates cellular growth and survival in response to environmental, extracellular, and intracellular stresses, such as hypoxia, reduced ATP levels, and DNA damage.^172^ Under glucose-deprived conditions, cellular energy levels decrease significantly, leading to the activation of AMPK, a metabolic regulator that senses energy stress. AMPK activation inhibits mTORC1 activity by directly phosphorylating Raptor or indirectly through TSC2 phosphorylation.^148^ Moreover, low glucose levels suppress mTORC1 signaling by inhibiting RAG GTPase activity, particularly in AMPK-deficient cells. A study by Dai et al. demonstrated that mTORC1 activation in response to glucose can be modulated *via* the AMPK-mediated phosphorylation of WDR24.^179^

Amino acids not only are essential for protein synthesis but also serve as vital reservoirs of carbon and energy for various metabolic signaling pathways.^180^ The modulation of amino acid levels in response to dietary changes is closely linked to mTORC1 activation. The discovery of RAG GTPases as key components in mTORC1 signaling, particularly in amino acid detection, has significantly advanced our understanding of mTOR signaling.^181^ These findings suggest that mTORC1 can effectively perceive glucose- and energy-related challenges through multiple molecular pathways.^182,183^

##### Energy regulation of mTOR

Mammalian TORC1 regulates cellular energy requirements through AMPK, which functions as an intracellular energy sensor. Enhanced glucose metabolism triggers mitochondrial activity by increasing AMP levels, disrupting the ATP:AMP ratio and subsequently activating AMPK. This activation leads to the phosphorylation of TSC2, which increases its GAP activity toward Rheb and suppresses mTORC1 activity.^184^ Additionally, AMPK directly phosphorylates Raptor, further reducing mTORC1 activity under low energy conditions.^148^

In addition, mTORC1 promotes cellular proliferation by shifting glucose metabolism from OXPHOS to glycolysis, a phenomenon known as the Warburg effect.^185^ mTORC1 signaling promotes the Warburg effect by upregulating and activating key glycolytic enzymes, including PKM2, HK2, and lactate dehydrogenase A (LDHA). This upregulation enhances glycolytic flux, supplying energy and essential building blocks for cell growth and division.^185,186^

Under hypoxic stress or oxygen deprivation, LDH catalyzes the conversion of pyruvate to lactate *via* NADH, potentially increasing lactic acid levels and leading to lactic acidosis. Lactic acidosis can promote oncogenesis by altering the tumor microenvironment (TME). HIF-1α is a well-known regulator that enhances the expression of glucose transporters and glycolytic enzymes and,^186,187^ facilitates glucose entry into cells and catabolism, respectively.^185–187^

Furthermore, mTORC1 can enhance the translation of HIF-1α. The activation of SREBP by mTORC1 also increases flux through the pentose phosphate pathway, leading to the production of NADPH and other intermediate metabolites necessary for cellular proliferation and growth.^188^

#### SIRT protein family and energy metabolism

Sirtuins are enzymes that remove acetyl groups from histones and depend on NAD for their function. They play a vital role in regulating key signaling pathways in both prokaryotes and eukaryotes and are involved in various biological processes.^189^ By modulating fat and glucose metabolism in response to energy fluctuations, sirtuins serve as essential regulators of the complex network responsible for maintaining energy balance.^190^ In mammals, the sirtuin family comprises seven proteins (SIRT1–SIRT7), each with distinct tissue specificity, subcellular localization, enzymatic activity, and target genes.^190^ Here, we discuss the specific roles of sirtuin family members in regulating cellular energy metabolism.

Cellular NAD availability, which regulates SIRT1, is influenced by various environmental signals.^191^ For example, SIRT1 activity is stimulated under conditions that elevate cellular NAD levels due to decreased energy status, such as fasting, caloric restriction, and physical exercise.^192–195^ Conversely, SIRT1 activity is reduced in high-energy states, which lower cellular NAD levels, such as during high-fat diet consumption or acute inflammatory responses.^196–198^ Additionally, modifications in NAD synthesis and breakdown can affect cellular NAD levels and, consequently, SIRT1 activity. SIRT1 is crucial for regulating liver metabolism, controlling fat release, promoting the conversion of white adipose tissue into brown fat, regulating insulin secretion, sensing nutrient availability by the hypothalamus, influencing obesity-related inflammatory responses in macrophages, and modulating circadian clock function in metabolic tissues.^199^ An intricate regulatory network that is responsive to nutritional, hormonal, and environmental signals operates at multiple levels to govern SIRT1 expression and activity and modulate cellular NAD levels.^200,201^ This network is vital for maintaining appropriate SIRT1 levels in response to diverse environmental stimuli.

SIRT2, the primary sirtuin localized in the cytoplasm, shows decreased expression during the transition from mitochondrial OXPHOS to glycolysis when cells transform into cancer cells. This reduction leads to increased acetylation and activation of key glycolytic enzymes.^202^ Hamaidi et al. demonstrated that SIRT2 interacts with eight glycolytic enzymes: HK1, phosphofructokinase (PFK), ALDOA, GAPDH, PGK1, ENO1, PKM, and LDH.^203^ Cha et al. found that SIRT2, rather than SIRT1, regulates the acetylation levels and activities of glycolytic enzymes (ALDOA, PGK1, ENO1, and GAPDH), highlighting the crucial role of SIRT2 in the glycolytic pathway. Moreover, GCK facilitates glucose consumption through glycolysis by converting glucose to G6P.^204^ SIRT2 activates GCK by deacetylating GKRP, which regulates GCK expression. This deacetylation causes GKRP to dissociate from GCK, thereby promoting hepatic glucose uptake.^204^

Numerous studies have shown that SIRT3 is expressed predominantly in mitochondria-rich tissues such as the liver, muscle, heart, brain, and kidney.^205^ SIRT3 functions as a deacetylase, regulating mitochondrial acetylation levels, which are closely linked to metabolic processes such as OXPHOS, FAO, and the TCA cycle across various organs. The enzymatic core of SIRT3 includes an NAD binding domain, a zinc-binding motif, and substrate-binding sites. SIRT3 activity is directly regulated by its metabolic cofactor (NAD^+^) and the resulting byproduct, nicotinamide. NAD^+^ facilitates the deacetylation process of SIRT3,^206^ whereas nicotinamide inhibits it by promoting the reverse reaction through binding to the reaction product.^207^ SIRT3 expression is significantly enhanced by caloric restriction, a critical factor in its regulation. The essential role of SIRT3 during fasting was confirmed by the identification of multiple deacetylation sites in 3-hydroxy-3-methylglutaryl CoA synthase 2, an enzyme regulating ketone body production, crucial energy sources for the brain when blood glucose levels are low.^208^ Additionally, SIRT3 regulates metabolism by facilitating the deacetylation and activation of isocitrate dehydrogenase 2 (IDH2), a key enzyme in the TCA cycle. SIRT3 also enhances the deacetylation of components within the OXPHOS complexes I53, II54, and III55, which are involved in the final stage of mitochondrial aerobic respiration.^206^ SIRT3 further plays a role in the deacetylation and activation of GDH within the TCA cycle, although the physiological significance of this function remains unclear.^209^

SIRT4 modulates the efficiency of OXPHOS by regulating ANT2 and its overexpression is associated with elevated ATP levels, while its deficiency is linked to mitochondrial uncoupling, resulting in increased oxygen consumption.^210^ This effect may be mediated by retrograde signaling from the mitochondria to the nucleus, which regulates ATP levels *via* the ANT2/AMPK/PGC-1α (peroxisome proliferator-activated receptor-γ coactivator-1α) signaling pathway in cases of SIRT4 deficiency.^210^ The potential involvement of other SIRT proteins in energy metabolism has not been extensively explored and warrants further investigation. Given the significant impact of sirtuins on energy metabolism, the development of sirtuin modulators is considered a promising avenue for drug discovery to address unmet needs in both common and rare diseases.^211^

#### Electrolytes and energy metabolism

Electrolytes such as potassium (K^+^), calcium (Ca^2+^), and magnesium (Mg^2+^) play crucial roles in modulating various metabolic pathways and enzyme activities, thereby regulating cellular energy metabolism. Among these ions, Mg^2+^ serves as an essential cofactor for numerous enzymes, including those pivotal in glycolysis and the TCA cycle, such as HK2, PFK, and PK.^212^ Disruption of Mg^2+^ homeostasis within mitochondria not only impacts the morphology and dynamics of mitochondria but also disturbs ATP synthesis and energy metabolism. K^+^ participates in diverse cellular metabolic processes, such as protein synthesis, carbohydrate metabolism, and the maintenance of enzyme activity.^213^ Adequate K^+^ concentration is indispensable for amino acid transport into cells during protein synthesis. In carbohydrate metabolism, both glycogen synthesis and glucose oxidation necessitate the involvement of K^+^. Furthermore, K^+^ activates ATPase in the TCA cycle and provides energy for the Na^+^-K^+^ pump to maintain normal cellular membrane function. The accumulation of Ca^2+^ within mitochondria serves not only as a buffering system but also as a signaling pathway regulating energy metabolism.^214^ Under physiological conditions, increased Ca^2+^ levels within mitochondria activate OXPHOS, stimulating ATP synthesis, inducing depolarization of the mitochondrial membrane, enhancing cellular respiration, activating Ca^2+^-dependent mitochondrial enzymes, and subsequently facilitating energy accumulation within mitochondria.

#### Energy metabolism and cell death

Energy metabolism is closely related to cell death in a way in which energy and energy mutually influence each other. In the execution process of apoptosis, the activation of caspases triggers the cleavage of nuclear DNA and cytoplasmic shrinkage, requiring significant energy support. Apoptotic cells typically upregulate the glycolytic pathway to provide additional energy, and mitochondria also participate in regulating apoptosis. Additionally, the functionality and activity of glucose transporter (GLUT) proteins can influence the cell’s apoptotic process.^215^ There is a close connection between lipid metabolism and ferroptosis. Intracellular iron overload can lead to increased oxidative stress reactions, resulting in the accumulation of lipid peroxides, which in turn impair mitochondrial function and cause abnormal cell energy metabolism. As the primary source of ROS, mitochondria can stimulate cells to undergo ferroptosis. Intermediate metabolites in the TCA cycle, such as alpha-ketoglutarate, oxaloacetate, succinate, and malate, can effectively replace glutamine and induce ferroptosis.^216^ On the other hand, mitochondrial metabolic pathways such as fatty acid beta-oxidation can reduce lipid peroxidation, thereby inhibiting ferroptosis.^217^ The glycolytic pathway affects ferroptosis by regulating the redox reactions of cells. Aberrant expression of G-6-P dehydrogenase may induce ferroptosis by disrupting NADPH and GSH metabolism.^218^ L Lactic acid, a product of glycolysis, can upregulate the expression of the ferroptosis-related proteins GPX4 and FSP1 and increase the levels of NADH, NADPH, and GSH, thereby inhibiting ferroptosis.^219,220^ Many regulatory molecules of energy metabolism also affect cell death. For instance, the activation of AMPK can inhibit ferroptosis,^221^ whereas the interaction between AKT and p53 can influence the execution of apoptosis.^222^ These interconnected molecules and pathways collectively form a complex regulatory network between cell death and energy metabolism, revealing the intricacy and diversity of cellular activities.

#### Role of the gut‒liver‒brain axis in energy metabolism

The intricate multi-directional connections formed between the intestines, liver, and brain constitute the gut‒liver‒brain axis. This axis regulates the body’s complex energy metabolism network through the exchange of hormones, cytokines, and nutritional metabolites. Initially proposed for regulating hepatic glycogen metabolism and maintaining energy balance, the gut‒liver‒brain axis plays critical roles in metabolic regulation. When blood glucose levels rise, the hypothalamus activates the parasympathetic nervous system, stimulating hepatic glycogen synthesis, and promoting insulin release from the pancreas to lower blood glucose levels. Conversely, when blood glucose levels decrease, the hypothalamus triggers the sympathetic nervous system, facilitating hepatic glycogen breakdown to maintain stable blood glucose levels. Moreover, the hypothalamus influences feeding and glucose metabolism regulation through appetite suppression and promotion signals.^223^ In the central nervous system, leptin enhances triglyceride outflow *via* the vagus nerve, reducing hepatic fat synthesis and thereby inhibiting hepatic fat deposition.^224^

Moreover, the liver and intestines can secrete metabolites that mutually influence each other and modulate brain signaling. For instance, the intestinal secretion of glucagon-like peptide 1 (GLP-1) influences the hypothalamus *via* the gut-brain axis, inducing insulin secretion, regulating appetite, and energy metabolism.^225^ Additionally, the gut microbiota plays crucial roles in the physiological functions of the gut‒liver axis and the gut‒brain axis. The gut microbiota produces short-chain fatty acids that enter the liver through the bloodstream, serving as an energy source for liver cells.^226^ These short-chain fatty acids can activate vagal afferent neurons, inhibiting food intake.^227^ Bile acids, synthesized in the liver through the cholesterol metabolism pathway, act as ligands for the transmembrane G protein-coupled receptor 5 (TGR5) and increase intestinal GLP-1 secretion. Bile acids not only regulate central metabolism and immune balance in the central nervous system but also modulate food intake through direct or indirect actions on the brain *via* TG5.^228^ Bile acids secreted by the liver promote FGF15 production by activating the FXR hormone receptor. FGF15 controls bile acid synthesis and hepatic lipid metabolism, impacting gluconeogenesis by inhibiting the CREB-PGC-1α pathway.^229^ During fasting, the liver perceives glycogen deficiency and transmits signals for fat breakdown through the hepatic vagus nerve, converting the energy source from carbohydrates to triglycerides to maintain energy balance.^230^ The complex interactions of the gut‒liver‒brain axis pose challenges for research but also offer potential targets for treating metabolic disorders.

#### Synergy of signaling pathways regulating energy metabolism across physiological states

The interconnected effects of signaling pathways can sense the energy status and regulate the storage and consumption of energy to ensure a balance between energy supply and demand, which is highly important for nearly all physiological states, including cell growth and differentiation, maintenance of biological rhythms, adaptation to environmental changes, and responses to multiple stresses.^231^ Under basal metabolic conditions, such as during rest, energy metabolism primarily supports essential functions through glucose oxidation in the brain and red blood cells, alongside FAO in muscle tissues.^232,233^ In exercise states, energy metabolism shifts dynamically: during high-intensity or short-duration exercise, glucose metabolism dominates as glycogen stores are rapidly utilized,^233,234^ while prolonged or lower-intensity activity increasingly relies on FAO to conserve glycogen.^235^ As exercise commences, the cellular AMP-to-ATP ratio increases, leading to the allosteric activation of AMPK.^236^ Once activated, AMPK stimulates FAO and glycolysis, which are essential for energy generation.^236^ AMPK also activates SIRT1, which then deacetylates and activates PGC-1α, increasing mitochondrial biosynthesis levels and enhancing energy production.^195,237^ Concurrently, AMPK activation curbs the activity of mTORC1, thereby inhibiting energy-consuming processes like gluconeogenesis, lipid synthesis, and protein synthesis.^238^ This strategic suppression aids in the reallocation of resources, ensuring that the energy balance is maintained during periods of scarcity. This coordinated regulation by the AMPK, SIRT, and mTOR pathways ensures energy balance and maintains cellular and systemic energy equilibrium during physical exertion.

In cold environments, energy metabolism undergoes distinct changes to maintain body temperature.^239^ The body increases thermogenesis, primarily through non-shivering thermogenesis in brown adipose tissue, which involves increased FAO and uncoupling of OXPHOS to generate heat rather than ATP.^240^ Within non-shivering thermogenesis, the β3-adrenergic signaling pathway plays a pivotal role.^241^ When exposed to cold, the sympathetic nervous system is activated, leading to the release of norepinephrine, which binds to β3-adrenergic receptors (β3-ARs) on the surface of brown adipocytes.^241^ This binding activates adenylate cyclase, increasing the production of cyclic AMP (cAMP), leading to the upregulation of thermogenic genes such as uncoupling protein 1 (UCP1).^242^ UCP1 facilitates proton leakage into the mitochondrial matrix, bypassing ATP synthesis and releasing energy as heat, which is essential for thermoregulation under cold conditions.^243^ Furthermore, energy-boosting pathways, including the AMPK and SIRT pathways, enhance FAO and glycolysis, increasing heat generation beyond ATP production under the influence of UCP1. Shivering thermogenesis, driven by muscle contractions, also amplifies glucose and lipid metabolism to meet the heightened energy demands of muscle activity.^244^

Under conditions of starvation or fasting, the body adeptly shifts its energy reliance towards stored fat, with increased FAO and ketogenesis providing energy to the brain and other tissues, while gluconeogenesis from amino acids sustains blood glucose levels.^245^ A coordinated series of signaling pathways are activated to regulate energy metabolism and preserve energy balance for vital physiological functions.^245^ Initially, during fasting, signaling pathways mediated by mTOR, insulin, and leptin are suppressed.^245,246^ This suppression reduces anabolic effects on glycogen, fat, and protein synthesis, while simultaneously promoting gluconeogenesis and lipolysis to furnish the body with energy. The diminished leptin levels invigorate the hypothalamus, subsequently increasing appetite and stimulating food intake behaviors.^247^ Furthermore, the hypothalamus releases corticotropin-releasing hormone, which prompts the pituitary gland to secrete adrenocorticotropic hormone. This sequence culminates in increased cortisol production by the adrenal cortex. Cortisol, in turn, stimulates gluconeogenesis and lipolysis, bolstering the body’s energy supply.^248^ Similar to the state during exercise, fasting increases a rise in the cellular AMP-to-ATP ratio, thereby activating AMPK signaling.^249^ This activation promotes catabolic processes that generate energy and, concurrently, inhibits mTORC1, halting energy-consuming processes such as gluconeogenesis, lipid synthesis, and protein synthesis. Additionally, in response to nutrient deprivation, AMPK phosphorylates the Unc-51-like kinase 1 (ULK1), initiating autophagy—a process that degrades and recycles cellular components to sustain energy balance.^167^

In summary, the interactive effects of signaling pathways are pivotal in orchestrating the complex dance of energy metabolism within cells, which allows the body to adapt swiftly to environmental changes, such as fluctuations in nutrient availability, shifts in oxygen levels, and temperature variations. Moreover, the signaling pathways also contribute to the intricate timing of biological rhythms, such as circadian rhythms, which regulate sleep patterns, hormone release, and other physiological processes.^250,251^ Additionally, the intricate balance maintained by these signaling pathways is vital for the prevention and treatment of a myriad of diseases. Disorders of energy metabolism, such as obesity and diabetes, are often linked to the dysregulation of these pathways. Understanding how these pathways interact can lead to the development of targeted therapies that can restore metabolic balance and improve patient outcomes.

## Energy metabolism disorders in the development of diseases

### Energy metabolism in neurodegenerative diseases

Neurodegenerative diseases such as Alzheimer’s disease (AD) and Parkinson’s disease (PD) are frequently linked to abnormal changes in energy metabolism and oxidative damage during their progression. These alterations, including impaired glucose uptake, may drive the onset and progression of these diseases.^252–255^ Disruptions in energy metabolism can result in insufficient cellular energy, oxidative stress, and cellular injury, leading to neuronal malnutrition, structural changes, and functional loss. Before the diagnosis of AD, there is generally a decrease in glucose uptake, but at this stage, brain oxygen, lactate, and ketone metabolism remain normal. As the disease progresses, reduced cerebral glucose metabolism is caused by a variety of factors, including decreased neuronal glucose uptake, impaired aerobic glycolysis and the tricarboxylic acid cycle, disrupted axonal transport function, and glial cell failure to supply energy to neurons. Additionally, neuroinflammation can promote glial cells to compete for glucose, further exacerbating neuronal glucose hypometabolism. This ultimately leads to synaptic loss and neuronal death.

#### Energy metabolism in AD

The high energy demand of the brain makes it particularly vulnerable to fluctuations in energy supply and mitochondrial function. In AD, a progressive neurodegenerative disorder, disruptions in energy metabolism contribute to the gradual degeneration and apoptosis of neuronal cells in the brain, ultimately causing severe cognitive impairment. Glucose oxidation serves as the primary energy foundation for the brain, with ketones, glycogen, and amino acids acting as supplementary sources. The metabolic processes of these substances are interlinked through the mitochondrial TCA cycle. The pathological progression of AD primarily results from mitochondrial dysfunction, characterized by reduced glucose uptake by neurons, alterations in glucose receptors, and changes in the metabolic phenotype of astrocytes. These dysfunctions are collectively regulated by metabolites, transport proteins, receptors, and enzymes. Such disruptions in energy metabolism facilitate the accumulation of brain amyloid-beta (Aβ) and aggregation of tau, leading to oxidative stress, inflammation, impaired autophagy, and various disease-causing cascades.^113^

##### Glycolysis

The brain accounts for approximately 25% of total body glucose consumption, primarily generating energy through OXPHOS. Research shows a significant reduction in brain glucose metabolism in patients with AD, shifting energy production from efficient aerobic oxidation to a less efficient glycolytic pathway. This shift decreases ATP generation efficiency, likely leading to an inadequate energy supply for neurons and accelerating pathological progression.^256,257^ Neurons may depend on lactate from neighboring astrocytes *via* the astrocyte–neuron lactate shuttle to meet their energy needs. Recent research indicates a significant inhibition of lactate production in astrocytes in AD, leading to impaired neuronal glucose metabolism. Inhibiting IDO1 activity can promote glycolysis and lactate production in astrocytes, thereby improving the energy metabolism state of neurons.^258^ Reduced glucose metabolism often precedes clinical AD symptoms, initially affecting the parietal-temporal lobes and posterior cingulate cortex before spreading to other regions, such as the frontal lobes.^259,260^ In the frontal lobe, glycolytic activity significantly increases in the inner, outer, and prefrontal areas but remains relatively low in the cerebellum and medial temporal lobe.^261^ Typically, the locus coeruleus activates astrocytes by releasing norepinephrine, which induces calcium influx, increases cAMP levels, and enhances aerobic glycolysis in astrocytes.^262,263^ However, as AD progresses, degeneration of the locus coeruleus shifts the energy source of the brain from glucose metabolism to a greater dependence on ketone bodies.^264^ Ketone bodies help compensate for ATP deficiencies and stabilize synaptic function by promoting mitochondrial biogenesis.^265,266^ Consequently, ketogenic diets may mitigate AD symptoms^267,268^ and have been shown to be effective in treating epilepsy.^269^

Declines in glucose metabolism in neuronal cells are often linked to changes in the expression of transport proteins and enzymes. Neurons rely on GLUTs to transport glucose from the bloodstream to critical brain regions, such as the hippocampus, as they cannot produce or store glucose. In AD patients, GLUT1 expression is often reduced in the hippocampus and frontal cortex,^270–272^ which may limit glucose entry and affect cellular energy supply. Research has also shown decreased GLUT3 expression in the hippocampus and parietal cortex, potentially further impairing glucose metabolism in AD.^272,273^ Conversely, GLUT4 expression increases in the hippocampus of AD patients, possibly as a compensatory mechanism to address impaired glucose uptake.^274,275^ Notably, β-amyloid peptides interact with these GLUTs, promoting their internalization and reducing glucose transport efficiency,^276,277^ which is considered a key factor in metabolic dysregulation. Additionally, key glycolytic enzymes such as aldolase, triosephosphate isomerase, GAPDH, phosphoglycerate mutase 1, and enolase become functionally impaired due to oxidative stress in AD, reducing glucose metabolism efficiency.^272^

A hallmark of AD is the abnormal accumulation of Aβ peptides and the associated inflammatory responses in brain tissue. Under physiological conditions, microglia are essential for maintaining neuronal energy, with the expression of Trem2 playing a crucial role in microglia-mediated synaptic refinement.^278^ However, in disease progression, microglia play a critical role in this process by exhibiting metabolic changes distinct from those in neuronal cells. Stimulation by LPS and Aβ induces microglial polarization towards the M1 phenotype, which involves significant reprogramming of glucose metabolism from OXPHOS to glycolysis, which is dependent on the mTOR-HIF-1α pathway.^279^ Activated M1 microglia demonstrate show enhanced glucose uptake and increased activities of HK, G6P dehydrogenase (G6PDH), PFK1, and LDH, leading to elevated lactate release.^280^ This increased glucose uptake is due to the upregulation of GLUTs, with GLUT1, GLUT4, and GLUT6 being critical for M1 microglia activation.^281,282^ Furthermore, the glycolytic pathway leads to G6P accumulation, which can enhance ROS production *via* the pentose phosphate pathway.^283^ This metabolic shift mirrors the “Warburg effect” observed in tumor cells.^284^ Under glucose scarcity, microglia utilize lactate as an alternative energy source to meet their metabolic needs.^285^ In contrast, M2 microglia maintain a functional TCA cycle and higher OXPHOS levels, supporting their phagocytic activity and potentially mitigating AD. In the future, conducting more metabolism-related polarization studies on microglia to delineate their roles and mechanisms may be a crucial research direction.^286^ Furthermore, due to the complexity of microglia in immune metabolism regulation, further research on potential therapeutic drugs and treatment methods involving metabolic remodeling regulation is warranted. Given the close relationship between microglial cells and neurons, targeting different cell types simultaneously for treatment will also be a future research focus.

In AD, multiple signaling molecules regulate glycolysis, with inflammation and oxidative stress playing significant roles. The inflammatory environment in AD triggers the release of cytokines such as interleukin-1β (IL-1β) and tumor necrosis factor-α (TNF-α), which can reduce GLUT expression in brain cells.^287,288^ Oxidative stress damages cell membranes and disrupts proteins, including GLUTs, crucial for energy metabolism.^289^ Throughout AD progression, a shift from OXPHOS to glycolysis occurs, driven by the mTOR-HIF-1α signaling pathway. Activation of this pathway induces metabolic changes that may impair normal microglial function.^290,291^ Furthermore, genetic risk factors for AD, such as the loss of TREM2, reduce the expression of GLUTs, glycolytic enzymes, and HIF-1α. However, interferon-γ (IFN-γ) can reverse glycolytic defects and inflammatory dysfunctions in microglia, potentially alleviating AD-related symptoms.^292^ The activation of STAT3 and STAT6 is also crucial for energy metabolism and microglial polarization, which are essential for maintaining cellular homeostasis and function.^293,294^

AMPK regulates GLUT3 translocation to the membrane; however, Aβ inhibits AMPK activity, affecting glucose transport.^295,296^ Additionally, studies have shown a correlation between PGC-1α expression levels and amyloid protein generation.^297^ In vitro experiments have found that oligomeric Aβ increases PGC-1α and SIRT1 levels, disrupting their interaction, which is crucial for maintaining metabolic balance. Insulin is vital in the hippocampus for promoting GLUT4 translocation to the plasma membrane, enhancing glycolysis, and improving spatial memory in rats.^298^ Insulin stimulates glucose uptake and glycogen accumulation in astrocytes by activating GLUT4.^299^ The pathogenesis of AD is often linked to insulin resistance, with PI3K playing a central role in insulin signaling, including GLUT translocation, glycogen synthesis, lipid and protein synthesis, anti-lipolytic effects, and hepatic gluconeogenesis.^300^ PI3K exerts effects through the Akt/PKB and protein kinase C cascades,^300^ with Akt activation inhibiting GSK3 *via* mTOR and downstream elements, promoting glycogen synthesis.^301^ ApoE2 protects against AD, whereas ApoE4 is associated with impaired glucose metabolism.^302^ This difference may be due to insufficient expression of insulin signaling-related genes, such as insulin receptor substrate, peroxisome proliferator-activated receptor γ (PPAR-γ), and insulin-degrading enzyme.^303^ Under normal conditions, SIRT3 levels are high in the brain but decrease in the frontal cortex of AD patients. SIRT3, located in the inner mitochondrial membrane, plays a crucial role in maintaining mitochondrial function.^304^ As a deacetylase, SIRT3 regulates p53-induced mitochondrial damage, preventing ROS accumulation and protecting neurons. Dysregulation of SIRT3 may serve as a biomarker for mitochondrial damage in AD, suggesting that modulating SIRT3 activity could be a novel therapeutic strategy.^305^ However, the efficacy of the aforementioned experimental animal model studies in transitioning to clinical trials still needs improvement, emphasizing the complexity of molecular regulatory signals. Research on the regulatory mechanisms of AD remains an enduring focal point.

##### Aerobic oxidation

In AD, cerebral features extend beyond impaired glucose metabolism to include microglia-driven inflammatory responses, mitochondrial dysfunction and heightened oxidative stress.^306–308^ Compared with controls, AD patients show elevated lactate and pyruvate levels and decreased succinate, fumarate, and glutamine levels, suggesting disrupted mitochondrial glucose metabolism.^309^ Pyruvate, a glycolysis product, typically enters mitochondria to participate in the TCA cycle. As a critical substrate for the pyruvate dehydrogenase (PDH) complex, pyruvate links glycolysis to the TCA cycle; however, PDH flux is reduced in AD.^306^ The activity of mitochondrial respiratory chain complexes I-IV is significantly diminished in AD, likely due to Aβ-mediated inhibition. Mitochondrial energy metabolism disorders, such as decreased TCA cycle enzyme activity, impaired respiratory chain function, reduced ATP production, and increased free radical and ROS levels, support the “mitochondrial cascade hypothesis” in AD pathogenesis.^310^

Pro-inflammatory activation further disrupts the TCA cycle, leading to substrate accumulation (e.g., citrate, succinate, and aconitate). This exacerbates intracellular inflammation, inhibits OXPHOS, reduces ATP production efficiency, and increases saturated fatty acid synthesis. The accumulation of fatty acids can affect membrane phospholipids, increasing cell susceptibility to lipid peroxidation and inflammatory signaling, thus enhancing neuroinflammation.^311,312^ NADPH is crucial for energy supply and redox balance. Mitochondrial NADPH production depends on nicotinamide nucleotide transhydrogenase (NNT), IDH2, and malic enzymes, with NNT contributing up to 50% of total NADPH output.^313^ In AD mouse models, NNT expression is reduced,^314^ and the activities of key metabolic enzymes, such as aconitase, α-KG dehydrogenase, and malic dehydrogenase (MDH), are inhibited,^315,316^ further impairing energy metabolism and antioxidant capacity. Under pathological conditions, NNT no longer catalyzes NADPH production but promotes NADH generation in reverse mode, leading to a reduced clearance of hydrogen ions by the redox system.^317^ However, further in-depth research is needed to understand the specific reverse mode, particularly its role in the brain.

Oxidative stress is crucial in neurodegenerative diseases, especially in the early stages of AD, where it is closely linked to mitochondrial dysfunction.^318^ Dysfunctional mitochondria produce less ATP and excessive ROS, contributing to the oxidative imbalance observed in AD. Enzymes essential for glycolysis, the TCA cycle, and the ETC are oxidized in the AD brain, leading to decreased activity. This inhibition disrupts glucose metabolism, reduces ATP synthesis, impairs neuronal function, causes synaptic loss, and promotes neurodegeneration.^319^ Variations in TREM2 are associated with several neurodegenerative diseases, including AD. Cells lacking TREM2 show impaired energy metabolism reduced mitochondrial quality, and abnormal organelle structure.^278^ Tau protein-activated microglia can cause metabolic abnormalities, such as abnormal succinate accumulation and TCA cycle disruption, which exacerbate neuroinflammation.^320^ The SIRT family of proteins regulates processes related to AD pathogenesis, including tau protein aggregation, mitochondrial dysfunction, oxidative stress, and neuroinflammation.^321^ Additionally, Aβ peptides and tau proteins can activate the PI3K/Akt/mTOR signaling pathway in microglia, which is crucial for regulating energy metabolism and ATP production.^322,323^ In light of these findings, current research is also exploring the therapeutic potential of antioxidants in neurodegenerative diseases. However, the efficacy of individual antioxidants is quite limited, suggesting that antioxidants are more suitable as adjunctive treatments, further prompting us to consider whether oxidative stress imbalance is a cause or a consequence of neurodegenerative diseases.

##### FAO

In AD, lipid metabolism dysregulation is closely linked to disease onset and progression. Long-chain fatty acids in the brain are synthesized de novo from acetyl-CoA.^324^ Fatty acid synthase (FASN) levels are elevated in AD patients, particularly around amyloid plaques.^325^ ACC activity is increased in the brains of AD mice.^326^ Elevated free fatty acids in the cerebrospinal fluid of AD patients can be neurotoxic, causing mitochondrial uncoupling and bioenergetic dysfunction.^327^ β-Oxidation of fatty acids is essential for their entry into the TCA cycle for energy production. This pathway helps meet the increased energy demands of the AD brain.^328^ Therefore, the timely degradation of free fatty acids, especially peroxidized fatty acids, is crucial for maintaining brain health because of their potential negative impact on cellular function.^327^ In patients with AD and elderly individuals, β-oxidation is increased to address increased energy demands.^329^ Increased FAO reduces triglyceride accumulation and lipid-induced inflammatory responses.^330,331^ There is controversy regarding the role of fatty acids as fuel substrates in meeting the brain’s energy demands.^328^ This discrepancy may stem from mitochondrial FAO and its association with oxidative stress and ROS generation compared to the oxidative breakdown of glucose. Neurons are particularly susceptible to the oxidative-redox environment due to their limited antioxidant capacity. In this scenario, transferring fatty acids from neurons to astrocytes, thereby utilizing the capacity of the cells for fatty acid degradation, may be an effective therapeutic approach.^332^ In this process, APOE4 exerts inhibitory regulatory effects, reducing transport efficiency, whereas APOE3 acts as a facilitator.^333^ Apart from ApoE, other AD risk factor genes like ABCA1, ABCA7, and PICALM are involved in fatty acid clearance.^334^ Further research on the regulatory molecules involved in fatty acid clearance in AD, specifically inhibitory factors, may mitigate the pathological progression of AD by alleviating oxidative stress in neurons.

FAO is also linked to insulin levels. In AD patients, cerebrospinal fluid insulin levels are significantly lower than plasma insulin levels. This insulin deficiency may cause substantial lipolysis in adipose tissue, releasing large amounts of fatty acids that, if not efficiently utilized, could impair cellular functions. In mouse models, neuron-specific insulin receptor knockout resulted in increased body weight, insulin resistance, and impaired glucose tolerance.^335^ Furthermore, pro-inflammatory factors such as LPS or IFN-γ may suppress genes involved in FAO.^336^ The PI3K/Akt signaling pathway regulates carbohydrate and lipid metabolism, affecting glucose uptake and utilization. PPAR-γ agonists have been shown to improve lipid and glucose metabolism and may reduce the pathological burden of Aβ plaques. These findings highlight the importance of maintaining proper insulin levels and regulating fatty acid metabolism in the prevention and treatment of AD.

AD is a complex pathological and physiological cascade disorder primarily caused by disrupted glucose metabolism. While research on neurodegenerative diseases has advanced significantly, with increasing insights from cell lines and murine animal models highlighting the bidirectional link between energy metabolism and brain inflammatory responses, the specificity of human specimens is limited to postmortem brain tissues. This limitation contributes to discrepancies often observed between clinical trial outcomes and animal models. The specific metabolic processes, such as the role of glucose and lipid metabolism disorders in driving neurodegenerative disease progression, and whether neuroinflammation is the cause of energy metabolism disorders, or merely a consequence, remain subjects of debate. Currently, the focus tends towards oxidative stress disturbances, inflammation, and other mechanisms as common pathological factors. Research on the multifactorial nature of the disease has made some progress over the past few decades, but the true etiology remains intricate, and involves genetic and lifestyle factors, necessitating further exploration.

#### Energy metabolism in PD

PD is a chronic, progressive neurodegenerative disorder primarily characterized by the loss of dopaminergic (DA) neurons in the substantia nigra (SN), resulting in reduced DA activity in the nigrostriatal pathway. Aging is a major risk factor for the development of PD and leads to significant alterations in energy metabolism,^337,338^ particularly manifested as mitochondrial dysfunction and impairments in the TCA cycle and ETC.^339^

##### Glycolysis

Disruption of glucose metabolism is closely linked to the progression of PD, potentially because of the negative impact of impaired glucose metabolism on the DA system.^340^ Although aerobic glycolysis is less efficient at producing ATP than OXPHOS is, rapid ATP production is critical for neuronal synapse formation.^341^ However, in the pathological state of PD, neurons exhibit significantly reduced glucose utilization efficiency.^342^ Studies suggest that activating glycolysis to supplement energy can provide neurons with the necessary ATP, thereby alleviating the symptoms of PD.^343^

Similar to observations in AD, the reprogramming of microglial energy metabolism is implicated in the pathogenesis of PD.^344^ The neuroinflammation induced by M1-type microglia has been closely linked to the progression of PD.^345^ Inhibiting specific metabolic pathways in M1-type microglia or promoting their shift to the M2 phenotype can significantly reduce neuroinflammation, thereby protecting DA neurons from damage. In M1-type microglia, increased pentose phosphate pathway activity and aerobic glycolysis are associated with excessive production of ROS. The accumulation of ROS can activate the nuclear factor κB (NF-κB) signaling pathway, a critical inflammatory signaling pathway that promotes neuroinflammation and may facilitate the onset and progression of PD.^346–348^ Nr1h4 inhibits the activation of NF-κB, thereby suppressing neuroinflammation in PD.^349^ In PD animal models and brain tissues of PD patients, the activation of microglia along with the infiltration of inflammatory factors such as TNFα, IL-6, Nos2, and COX2 can be observed, attracting T cells to cross the blood-brain barrier.^350,351^ This phenomenon may be associated with the activation of the JAK/STAT pathway,^352^ providing in vivo evidence for the involvement of inflammation and immune responses in the progression of PD.

In PD, the expression of GLUTs in neurons significantly changes. Although there are differing viewpoints within the scientific community, the prevailing research suggests that GLUT expression levels are downregulated.^353^ Thus, increasing GLUT expression levels may help mitigate the progression of PD.^354^ Critical glycolytic enzymes, such as PGK1, HK2, and PFK, exhibit insufficient activity in the pathogenesis of PD.^355^ Notably, the overexpression of HK2 has been shown to alleviate PD symptoms by enhancing glycolysis.^356^ Furthermore, pharmacological agents such as meclizine, which activates the glycolytic enzyme PFK, can increase glycolytic rates, suggesting a potential therapeutic strategy against PD.^357^

PARK7, a protein closely associated with PD, harbors mutations in familial PD cases that may reduce protection against damage induced by 1,3-BPG. 1,3-BPG can form reactive intermediates and react with amino groups, but PARK7 disrupts these intermediates, preventing improper modifications of proteins and metabolites.^358^ The Wnt/β-catenin signaling pathway is a critical factor in the development of many neurodegenerative diseases.^359^ In PD, downregulation of this pathway is a hallmark of the disease.^359^ Impaired function of the Wnt/β-catenin pathway can lead to restricted expression of its target genes, such as pyruvate dehydrogenase kinase 1 (PDK1), MCT1, c-Myc, cyclin D1, and LDHA, which play important roles in enhancing glucose metabolism.^360^ Additionally, studies have shown that the expression of PGC-1α-responsive genes is downregulated in the brains of PD patients, possibly due to hypermethylation of PGC-1α transcriptional elements, leading to bioenergetic dysregulation.^361^ Disruption of glycolytic metabolism is a significant energy metabolic alteration in PD. However, current research on its regulation remains limited. Future studies should integrate metabolomics, spatial transcriptomics, CRISPR screening, and other technologies to identify more potential regulatory factors, thereby offering novel diagnostic and therapeutic targets.

##### Aerobic oxidation

Current research widely acknowledges that impaired mitochondrial energy production plays a crucial role in promoting the progression of PD. Multiple factors can lead to mitochondrial dysfunction in PD, ultimately affecting ATP synthesis. Genes associated with PD, such as PTEN-induced kinase 1 (PINK1), Parkin, DJ-1, LRRK2, and ATP13A2, are directly or indirectly involved in maintaining mitochondrial integrity and function. Mutations in these genes are linked to familial PD and are closely associated with mitochondrial dysfunction.^362–365^ PD-related gene mutations may inactivate PINK1/Parkin, inhibit mitophagy, and result in the accumulation of dysfunctional mitochondria, thereby triggering apoptosis.^366^ Given the high energy demands of DA neurons and the reliance on OXPHOS for energy, impairments in mitochondrial OXPHOS are particularly critical in the progression of PD.^367,368^ Sustained mitochondrial OXPHOS increases the risk of oxidative damage, and impaired mitochondria in PD release more ROS. Excessive ROS accumulation damages cellular components, including proteins, lipids, and DNA, exacerbating mitochondrial dysfunction and potentially initiating a cascade of neurodegenerative processes.^369–371^ The abnormal aggregation of α-synuclein (α-Syn) into Lewy bodies is closely associated with DA neuron dysfunction. Mechanistically, α-Syn oligomers interact with mitochondrial respiratory chain complex I, impairing its function, leading to mitochondrial membrane potential depolarization, and promoting the release of proapoptotic factors into the cytoplasm. Once released, these factors can interact with intracellular antiapoptotic and survival-promoting proteins, inducing mitochondria-mediated apoptosis.^372,373^ Recent clinical studies suggest that the initiation of deformation in DA neurons is mediated by the TAU, rather than the aggregation of α-Syn.^374^ The pathological similarities observed in these neurodegenerative diseases imply the existence of potential common pathogenic mechanisms that may impact the progression of each disease. In the pathogenesis of α-Syn, HSPB6 inhibits the aggregation of α-Syn through a lipid-dependent mechanism.^375^ Additionally, environmental toxins can contribute to PD by disrupting the mitochondrial ETC, thereby affecting OXPHOS.^376,377^ The opening of the mitochondrial permeability transition pore (MPT) can lead to the collapse of the mitochondrial membrane potential, impaired OXPHOS, and eventual cell death.^378^

In DA neurons, reduced expression of PGC-1α has been observed, suggesting that PGC-1α dysfunction may play a role in the clinical pathogenesis of PD.^379^ In PGC-1α knockout mouse models, DA neurons exhibit increased sensitivity to the neurotoxin MPTP, potentially due to the lack of PGC-1α-mediated induction of antioxidant responses.^380^ The sirtuin protein family plays crucial regulatory roles in neurodegenerative diseases. Specifically, SIRT2 is involved primarily in the development of the nervous system, whereas SIRT1 is closely linked to cellular energy metabolism and survival. Moreover, SIRT3 and SIRT4 are central to the regulation of mitochondrial metabolism.^381,382^ SIRT1 has been shown to activate antioxidant pathways through the deacetylation of NRF2, thereby exerting neuroprotective effects in various PD models.^383^ In MPP^+^-induced PD cell models and primary DA neurons, SIRT1 enhances mitochondrial biogenesis through the deacetylation of PGC-1α, providing neuroprotection.^384^ Activation of AMPK can increase SIRT activity or directly phosphorylate PGC-1α. This is attributed to the elevation in NAD^+^ levels. Mitochondrial SIRT is also capable of activating the AMPK signaling pathway, increasing autophagic function, and accelerating the clearance of the α-Syn protein.^385^ SIRT3 can reduce cell death, inhibit the generation of inflammatory cytokines, and eliminate mitochondrial ROS through mitochondrial pathways, thereby exerting neuroprotective effects. Additionally, reduced levels of dynamin-related protein 1 (DRP1) have been observed in the astrocytes of PD patients. A decrease in DRP1 levels alters mitochondrial morphology, indicating that mitochondrial dynamics may be impaired in the astrocytes of PD patients.^386^ Mutations in the mitochondrial inner membrane protein CHCHD2 result in severe mitochondrial damage and a subsequent metabolic shift towards glycolysis, leading to the destruction of DA neurons.^387^ Recent studies have further emphasized the role of mitochondrial dysfunction in promoting the pathology of PD, with various regulatory mechanisms being uncovered. However, in-depth exploration of these mechanisms is still crucial for unraveling the pathogenesis of PD.

##### FAO

Dysregulated lipid metabolism is closely associated with PD.^388,389^ Intracellular lipid accumulation can lead to mitochondrial dysfunction, which not only reduces the number of mitochondria but also exacerbates lipid accumulation, creating a vicious cycle.^390^ The abnormal aggregation of α-syn is a hallmark of PD, and the accumulation of lipids, including polyunsaturated fatty acids (PUFAs) and cholesterol, plays a crucial role in this process.^391^ In PD, hyperactive neurons may produce toxic fatty acids, which are transported to astrocyte lipid droplets *via* APOE-positive lipid particles for detoxification through mitochondrial β-oxidation.^392^ However, impaired mitochondrial function in astrocytes reduces their capacity to metabolize fatty acids, leading to neurotoxicity from fatty acids, which could further exacerbate the pathological process of PD. In the brains of PD patients, decreased activity of SIRT6 has been observed, which may be associated with mitochondrial dysfunction.^393^ Studies indicate that a lack of SIRT6 leads to reduced mitochondrial gene expression, increased production of ROS, and accelerated mitochondrial decay, collectively triggering mitochondrial dysfunction.^394^ ACSL4 promotes lipid peroxidation, whereas the inhibition of ACSL4 can prevent the death of DA neurons.^395^ Recent studies have underscored the potential of the Lrrk2 gene as a diagnostic marker.^396^ However, to date, there are no biomarkers that can predict which individuals are more likely to develop PD. Over the past few years, research has increasingly recognized the critical role of fatty acid metabolism in energy disturbances in PD, with fatty acid-binding proteins such as FAP3 being targeted for therapy and serving as alternative treatment strategies for PD.^397^

##### Electrolytes

In the nervous system, ions such as Mg^2+^ and K^+^ play pivotal roles in establishing and conducting neuronal membrane potentials. These electrolyte ions maintain ion balance in neurons through ion channels and are pumped on the neuronal cell membrane, supporting normal neuronal function and neural signal transmission. Abnormal potassium ion channels may accelerate pathological α-Syn accumulation, increasing the risk of PD.^398^ Magnesium ions can inhibit neuroinflammation mediated by neuroglial cells by downregulating pro-inflammatory cytokines and oxidative stress, a phenomenon closely associated with age-related chronic disease development.^399^

Due to the high clinical, pathological, and genetic heterogeneity of PD, the disease can last for several decades, significantly increasing the difficulty in studying its etiology and treatment. Despite some progress, the exact causes and pathogenic mechanisms of PD remain incompletely understood. Clinical treatments only offer symptomatic relief and not a halt to disease progression. Therefore, there is a growing emphasis on personalized diagnosis and treatment where the prodromal phase of the disease can provide a therapeutic window for early intervention. Subsequent crucial research involves the development of improved non-invasive biomarkers to detect and monitor pathological progression, including pathways related to genetics (such as mitochondrial or synaptic dysfunction). Studying how factors like exercise, diet, and sleep affect energy metabolism in PD patients can lead to effective interventions through lifestyle modifications. Additionally, combining drug screening technologies to explore new applications for existing drugs may provide feasible short-term improvements in PD treatment. The emergence of new technologies like spatial transcriptomics and spatial metabolomics offers hope for identifying more potential metabolites in animal models, although effectively applying these methods to human samples remains a significant challenge.

### Energy metabolism in cardiovascular diseases

The heart, which is essential for life as a powerful pump, constantly requires ATP to maintain continuous beating.^400,401^ Fatty acids are the primary energy source, contributing 60–90% of the heart’s energy, and are transported into cardiomyocytes by CD36 and FATP, where they undergo β-oxidation to produce acetyl-CoA for the TCA cycle and ATP generation.^9,402^ In cardiovascular diseases, an increase in fatty acids is associated with abnormal lipid metabolism changes, leading to mitochondrial oxidative stress and the accumulation of toxic lipids. This lipid oxidation-reduction imbalance can promote ferroptosis. Glucose also plays a key role, as it enters cells through GLUT1 and GLUT4, with insulin enhancing glucose uptake by promoting GLUT4 translocation to the cell membrane.^403^ Additionally, ketone bodies, which constitute only 15–20% of the energy supply, are crucial for heart protection because they improve heart failure and hypertrophy and are easily converted to acetyl-CoA for energy.^378^ Ketone supplementation can mitigate negative heart remodeling and enhance cardiovascular function.^404,405^ The high mitochondrial content of the heart is pivotal for energy production, but it also generates significant amounts of ROS during OXPHOS.^406^ Mitochondrial dysfunction contributes to cardiac damage, leading to impaired mitophagy, altered enzyme activity in the ETC, and reduced ATP production.^407^ In the early and middle stages of cardiovascular diseases such as heart failure, myocardial energy metabolism remains relatively normal, with little or slight increase in fatty acid utilization; however, in the end-stage, myocardial cells transition from predominantly relying on fatty acid metabolism to predominantly relying on glucose metabolism for energy, yet glucose utilization rates are low. Moreover, decreased fatty acid utilization leads to the accumulation of lipid peroxides, resulting in myocardial cell apoptosis. As heart failure progresses to the end stage, the levels of metabolic products related to ketone oxidation and metabolic enzymes in the body usually increase.

In the cardiovascular system, Mg^2+^ promotes the release of NF-κB through its antioxidant effect by eliminating ROS, impacting downstream inflammatory signaling pathways, and regulating inflammation.^408^ Inflammation triggered by hypomagnesemia may affect lipid profiles by altering lipid peroxidation, leading to increased triglycerides in lipoproteins, accumulation of apolipoprotein B, and decreased high-density lipoprotein cholesterol, resulting in disrupted lipid metabolism. Recent studies have indicated that potassium regulation can counteract detrimental ion accumulation induced by hypoxia and ischemia (such as Ca^2+^ and Na^+^), maintain stable myocardial membrane potentials, and prevent mitochondrial dysfunction, which is crucial.^409^

Brown adipose tissue (BAT), a crucial thermogenic organ, plays a key role in promoting glucose and lipid metabolism, enhancing energy expenditure, and maintaining cardiac metabolic health. The presence of BAT can reduce the incidence of dyslipidemia and cardiovascular diseases.^410^ BAT with multilocular lipid droplets is rich in mitochondria and significantly expresses uncoupling protein 1 (UCP1), PGC-1α, and PRDM16.^411^ Active BAT promotes cardiac metabolism health by burning triglycerides and free fatty acids derived from glucose, thereby preventing lipid metabolism abnormalities, obesity, and insulin resistance.^412^ BAT increases glucose uptake and utilization and lowers blood glucose levels, thus exerting a positive effect on T2DM and cardiovascular diseases. Through the SIRT1-PGC1α-PPAR-γ pathway, BAT inhibits NF-κB to suppress inflammation.^413^ Factors secreted by BAT, such as NRG4, contribute to improving atherosclerosis by inhibiting endothelial inflammation and macrophage accumulation in plaques, protecting endothelial vessels from damage.^414^ Furthermore, BAT and its secreted protein BMP3b can alleviate cardiac ischemia-reperfusion injury.^415^ However, besides promoting energy expenditure and thermogenesis, BAT also inhibits adipose tissue inflammation and promotes mitochondrial amino acid catabolism, providing protective nutrients for crucial glucose metabolism.^416^ Beige adipose tissue, which is primarily distributed in epicardial fat tissue, is characterized by both brown and white adipose tissue. Like brown adipocytes, beige adipocytes possess multiple small lipid droplets, high mitochondrial content, and the expression of thermogenic genes such as UCP1 and Pgc1a.^417^ Under specific environmental, genetic, or pharmacological stimuli, beige adipocytes can develop in white adipose tissue (WAT). Upon stimulus withdrawal, the gene expression profile of beige adipocytes is altered, reverting to WAT characteristics.^418^ Brown and beige adipose tissues play significant roles in regulating energy balance and providing protection against cardiovascular diseases. Research into their potential roles and mechanisms holds crucial importance for disease treatment and prevention.

Multiple signaling molecules regulate cardiovascular energy metabolism, playing key roles in the transcription of metabolism-related genes, including AMPK, PGC-1α, SIRT, and PPARs. When the energy demand increases, AMPK is activated, increasing CD36 translocation to the membrane and promoting FAO.^400^ AMPK also induces mitochondrial biogenesis *via* PGC-1α phosphorylation and SIRT1 activation.^419^ In mouse models, PGC-1α regulates lipid metabolism by upregulating genes involved in the TCA cycle and mitochondrial FAO.^420,421^ PPARs facilitate cardiac fatty acid uptake and oxidation by binding FAs in the cytoplasm, transferring them to the nucleus, and interacting with the retinoid X receptor (RXR) to regulate FA metabolism genes.^422^ In addition to upregulating FA uptake proteins, PPAR also activates genes involved in OXPHOS, thus modulating mitochondrial functions.^423^ Angiopoietin-like proteins (ANGPTL) influence the clearance of FAs by inhibiting lipoprotein lipase activity, a process regulated by transcription factors such as PPAR and HIF-1α.^424^ Suppressing the expression of ANGPTL4 can improve abnormal glucose metabolism by enhancing insulin sensitivity; however, the specific regulatory pathways involved in cardiac energy metabolism still require exploration. In the heart, SIRT3 enhances glucose transport by activating GLUT1 and GLUT4 while regulating PFK activity, reducing TP53-induced glycolysis and apoptosis regulator (TIGAR) expression to increase glucose metabolism.^425,426^ SIRT3 deficiency impairs the mitochondrial membrane potential, induces ROS accumulation, and triggers NLRP3 inflammasome activation, exacerbating cardiac damage.^427^ Cardiac energy metabolism is further regulated by hormones. Insulin increases glucose uptake in cardiomyocytes by promoting GLUT4 translocation, whereas thyroid hormones increase energy production *via* mitochondrial OXPHOS.^400^ Adrenaline responds to increased energy demands under physiological or pathological conditions by activating the oxidation of various energy substrates in the heart.^400^

Owing to advancements in metabolomics, research on metabolic pathways in cardiovascular diseases is currently extensive, leading to the discovery of more biomarkers. Research on the regeneration of cardiac tissue based on energy metabolism has become feasible. However, translating findings from basic research into clinical applications still poses challenges.

#### Energy metabolism in ischemic heart disease

**Fig. 4:**
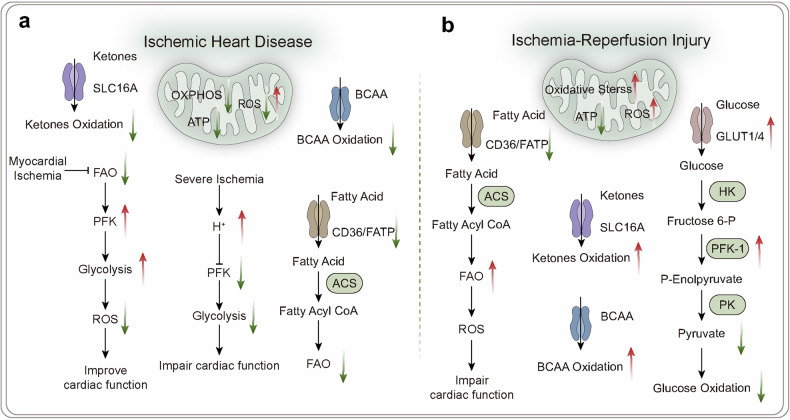
Energy metabolism in ischemic heart disease and ischemia-reperfusion injury. Under physiological conditions, the heart primarily relies on the oxidation of fatty acids entering the TCA cycle for energy supply. In various disease states (**a** ischemic heart disease and **b** ischemia-reperfusion injury), changes in cardiac energy metabolism led to increased production of ROS, ultimately affecting mitochondrial function and resulting in decreased ATP production, which impacts the heart’s ability to perform its functions. FAO fatty acid oxidation, PFK phosphofructokinase, BCAA branched-chain amino acids, HK hexokinase, PK pyruvate kinase, ACS acyl-CoA synthetase

Ischemic heart disease, caused by reduced coronary blood flow, profoundly affects cardiac energy metabolism (Fig. 4a). These effects include altered substrate utilization, OXPHOS dysfunction, impaired ATP synthesis, and mitochondrial dysfunction. These metabolic disturbances not only contribute to the onset of the disease but also accelerate its progression, leading to significant impairment of cardiomyocyte function. When blood flow to the heart is restricted, cardiomyocytes shift toward glycolysis to maintain their energy supply. This shift is driven by increased glucose uptake, GLUT translocation, reduced levels of glycolytic intermediates to stimulate glycogen breakdown, and elevated AMP levels, which activate AMPK. These changes increase GLUT4 translocation and PFK activation, increasing fructose 2,6-bisphosphate production.^428,429^ AMPK activation further promotes glycolysis and increases fatty acid oxidation, although fatty acid oxidation levels remain below normal under low blood flow. Despite this, FAs continue to be a primary energy source. Under conditions of low or absent cardiac blood flow, CD36 expression on the sarcolemma is downregulated,^430^ reducing or halting fatty acid β-oxidation, and glucose becomes the predominant substrate for ATP production.^431^ The diminished capacity of the heart to oxidize fatty acids results in lower citrate levels, which in turn enhances glucose uptake and glycolysis.^432^ Citrate, an allosteric regulator, typically inhibits PFK1 and PFK2, thereby reducing glycolysis.^433^

Under normal oxygen conditions, glycolysis accounts for approximately 5% of the energy requirements of the heart.^434^ In contrast, during hypoxia, glycolysis and lactate metabolism predominate.^435^ Reduced coronary blood flow exacerbates ischemia, where increased glycolysis temporarily supports ATP production and cardiac ion balance.^433^ However, severe ischemia leads to the accumulation of harmful waste, such as intracellular protons, negating the benefits of glycolysis. The accumulation of long-chain fatty acids within mitochondria impairs PDH activity, reducing glucose oxidation and increasing lactate and proton levels, which worsens myocardial injury.^436^ This also induces mitochondrial membrane hyperpolarization and increases ROS generation, further damaging cardiac tissue.^437^ In ischemic heart disease, mitochondrial function is hindered by depleted TCA cycle intermediates and free CoA.^438^ This dysfunction elevates succinate, a hallmark of ischemia, which is a primary source of ROS during reperfusion.^439^

Multiple signaling pathways regulate energy metabolism during heart ischemia. During heart ischemia, the AMPK/SIRT1/PGC-1α pathway exhibits abnormalities. Therefore, this pathway may serve as a crucial target for treating cardiovascular diseases. The ALDH2/SIRT3/HIF-1α axis plays a myocardial protective role, reducing 4-HNE levels post myocardial ischemia/reperfusion (I/R), resulting in decreased apoptosis, a reduced myocardial infarct size, and decreased ROS levels. Depletion of ALDH2 eliminates these beneficial effects.^440^

#### Energy metabolism during ischemia‒reperfusion injury

Ischemia‒reperfusion injury occurs when blood flow restoration to the heart exacerbates dysfunction and structural damage instead of recovery (Fig. 4b). During ischemia, oxygen deprivation suppresses critical metabolic pathways such as those involving fatty acids, ketone bodies, branched-chain amino acids (BCAAs), and glucose oxidation, drastically reducing ATP production. To compensate, cardiomyocytes shift to glycolysis for basic energy needs, although this source is insufficient for normal cardiac activity.^9^ In response to energy scarcity, 5’AMP accumulation activates the AMPK pathway, increasing phosphoinositide-dependent kinase-2 (PDK2) activity and GLUT1/4 expression to increase glycolysis. However, AMPK also suppresses the fatty acid transporter CD36, limiting fatty acid uptake and oxidation and thus aggravating metabolic imbalance.^400^ Furthermore, impaired BCAA metabolism triggers intermediate metabolite buildup, inhibiting PDH and disrupting glucose oxidation, potentially leading to increased reliance on glycolysis.^441^

During myocardial reperfusion, although reoxygenation and nutrient supply should theoretically restore OXPHOS, the glycolytic pathway initiated during ischemia provides only short-term energy and is unsustainable. This shift results in NAD^+^ depletion, lactate buildup, and a decrease in the intracellular pH, negatively impacting cellular function.^442^ The rapid reintroduction of oxygen and normalization of pH during reperfusion can lead to increased ROS production, causing mitochondrial damage and impeding ATP synthesis. The generation of ROS after ischemia‒reperfusion activates multiple pathways, such as the ATF4-CHOP, TLR4/TRIF, and USP7/p53/TfR1 pathways, triggering ferroptosis and promoting immune cell recruitment, further exacerbating inflammation.^443–445^ Moreover, calcium overload, mitochondrial dysfunction, and disrupted signaling pathways during reperfusion exacerbate cardiac damage. The activation of Nrf2 plays a role in protecting the heart from ischemia/reperfusion injury. Notably, OXPHOS impairment during ischemia limits oxygen utilization, decreasing glucose oxidation. Research highlights the importance of enhancing glucose oxidation to reduce ischemia‒reperfusion injury.^446^ Despite sustained high glycolytic rates, impaired glucose oxidation decreases ATP production, reducing cardiac efficiency.^447^

In ischemia-reperfusion injury, high-density lipoprotein (HDL) inhibits BID-mediated mitochondrial apoptosis activation by regulating the expression and phosphorylation of the anti-apoptotic factor BCL-XL. This is primarily mediated by the anti-apoptotic properties of sphingosine-1-phosphate (S1P) in HDL, which prevents the opening of mitochondrial permeability transition pores by activating the SAFE (Survivor Activating Factor Enhancement) and RISK (Reperfusion Injury Salvage Kinase) pathways.^448,449^ HDL may serve as a potential therapeutic strategy for ischemia-reperfusion injury.

#### Energy metabolism in heart failure

**Fig. 5:**
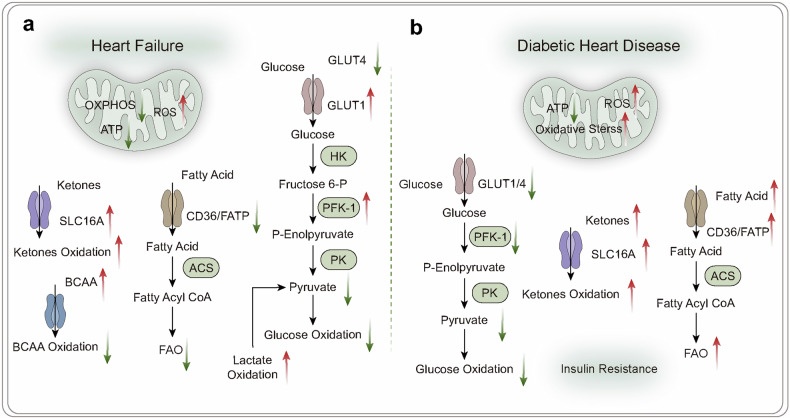
Energy metabolism in heart failure and diabetic heart disease. Under physiological conditions, the heart primarily relies on the oxidation of fatty acids entering the TCA cycle for energy supply. In various disease states (**a** heart failure and **b** diabetic heart disease), changes in cardiac energy metabolism led to increased production of ROS, ultimately affecting mitochondrial function and resulting in decreased ATP production, which impacts the heart’s ability to perform its functions. FAO fatty acid oxidation, PFK phosphofructokinase, BCAA branched-chain amino acids, HK hexokinase, PK pyruvate kinase, ACS acyl-CoA synthetase

Heart failure is a critical clinical syndrome characterized by a reduced ability of the heart to pump blood and failure to meet the body’s metabolic demands (Fig. 5a). This condition involves multiple disruptions in cardiac energy metabolism, including substrate utilization, mitochondrial function, and ATP synthesis. Chronic illnesses such as hypertension, diabetes, and obesity can trigger adverse metabolic shifts, resulting in Na^+^, H^+^, and Ca^2+^ overload, leading to cellular acidosis and damage, and increasing energy consumption in cardiac myocytes, leading to inadequate energy supply and potentially progressing to heart failure.^450^ A hallmark of heart failure is the reprogramming of myocardial energy metabolism, which includes decreased oxidation of fatty acids, glucose, and BCAAs, with a compensatory increase in glycolysis. Despite increased glycolysis attempting to offset reduced FAO, this approach yields approximately 30% less ATP than a healthy heart does, which is insufficient to meet energy needs.^451,452^ Moreover, impaired OXPHOS in mitochondria, often uncoupled from glucose oxidation, further compromises ATP production and cardiac efficiency in heart failure.^450^ This mitochondrial dysfunction, characterized by increased ROS and abnormal dynamics, is closely linked to the progression of heart failure.^453^ Elevated free fatty acid levels in the heart correlate with heart failure severity.^400^ The inhibition of PGC-1α transcription and deactivation of the PPAR/PGC-1α pathway results in increased activity of the crucial regulatory factor CPT-1 for fatty acid uptake, while the synthesis of the FAO regulator β-oxidase is inhibited.^454,455^ This leads to reduced FAO and increased lipid accumulation in cardiac myocytes. Aberrant lipid peroxides, through TLR4/NOX4 involvement, contribute to ferroptosis, mediating myocardial cell death and exacerbating heart failure. Inhibitors of ferroptosis can significantly improve the survival rate in a heart failure mouse model.^456^ Inadequate oxygen supply in failing hearts shunts some free fatty acids into nonoxidative pathways, producing toxic lipids such as ceramides and diacylglycerol, which further damage mitochondrial function and exacerbate heart failure progression.^457^ Disturbances in BCAA metabolism also play a critical role, with elevated BCAA levels detected in failing hearts, likely due to impaired BCAA oxidation during heart failure.^458,459^ Impaired BCAA catabolism/oxidation is associated with contractile dysfunction and heart failure development,^433^ and these metabolic disturbances are linked not only to cardiac dysfunction but also to cardiac remodeling and insulin resistance in heart failure patients.^433^ In heart failure, metabolic defects in fatty acid and glucose metabolism shift cardiac myocytes towards ketone body metabolism. Ketone bodies could serve as a potential alternative fuel for failing hearts, bypassing disrupted metabolic pathways such as β-oxidation and converting acetoacetate to acetyl-CoA. Recent studies have indicated that elevated levels of ketone bodies can increase the myocardial energy supply and alleviate cytokine-induced mitochondrial dysfunction and fibrosis.^460^ Enhancing ketone metabolism in heart failure patients is a feasible strategy for regulating cardiac function and improving prognosis, but further exploration is needed on the regulatory mechanisms governing the interconversion of ketone bodies and glucose‒lipid metabolism.

During heart failure, cardiac energy metabolism undergoes reprogramming to meet shifting metabolic demands. The expression of GLUT1 increases, paralleling enhanced glycolysis, indicating that GLUT1 may drive this increased glycolytic activity.^400^ Conversely, GLUT4 expression decreases, potentially reducing myocardial glucose utilization efficiency. In a normal adult heart, PKM1 levels are high, whereas PKM2 levels are low. PKM1 is crucial for maintaining a stable hemodynamic response, regulating mitochondrial energy production through pyruvate generation.^461^ However, during heart failure, decreased PKM1 and increased PKM2 levels are observed. The lack of PKM1 diminishes pyruvate production, inhibiting PDH activity. Since pyruvate is pivotal for entering the TCA cycle, its reduction decreases TCA flux, impairing mitochondrial energy output. This metabolic disruption exacerbates heart dysfunction and fibrosis caused by pressure overload. In contrast, overexpression of PKM1 offers cardioprotection against contractile dysfunction, which is essential for maintaining glucose uptake and glycolysis, thereby supporting ATP production and biosynthesis for cardiac function.^461^ In sharp contrast, PKM2 elevation is associated with pathological cardiac remodeling, acting as a detrimental factor in heart failure progression. Increased PKM2 activity may drive harmful metabolic reprogramming, leading to adverse structural and functional cardiac changes.^461^

In heart failure, energy metabolism changes involve complex regulatory pathways. The uncoupling of glycolysis from glucose oxidation is mediated by mitochondrial uncoupling protein-2 (UCP-2), which disrupts the entry of pyruvate into the mitochondria for oxidation. On the other hand, FTO maintains cellular glucose uptake and stabilizes glycolysis. Metabolic disturbances cause BCAA accumulation, activating mTOR signaling and promoting cardiac hypertrophy.^462^ Targeting BCAA metabolism or using mTOR inhibitors such as rapamycin may improve heart function.^463^ AMPK is pivotal in heart failure and is activated under stress to increase cardioprotection by enhancing GLUT4 translocation and glucose uptake.^464,465^ It also increases fatty acid uptake by moving CD36 to the membrane, inhibits ACC, reduces malonyl-CoA, and increases CPT1 activity.^466^ PGC-1α is key for mitochondrial biogenesis,^467^ with AMPK enhancing activity through direct phosphorylation or SIRT1-mediated deacetylation.^468^ SIRT3, which is crucial in cardiovascular health, is downregulated in heart failure, increasing PDH and ATP synthase acetylation and reducing mitochondrial activity.^469,470^ SIRT3 overexpression enhances SOD2 activity, lowering ROS,^471^ and suppresses fibrosis pathways such as the TGF-β/Smad pathway.^472,473^ PPAR-α, which is upstream of SIRT3, influences fatty acid metabolism. Its suppressed signaling in heart failure affects FAO, leading to functional impairment. Activating PPAR-α increases metabolic balance, reduces ischemia/reperfusion damage, and improves heart function and antioxidant capacity.^474,475^ Nrf2 inhibits oxidative stress sensitivity and cardiac damage in heart failure, thereby attenuating the progression of heart failure. The activation of Nrf2 is regulated by the Akt/GSK-3β/Fyn signaling pathway.^476^

Improving myocardial energy metabolism provides a new perspective for the prevention and treatment of heart failure. Metabolic therapies that lower fatty acid intake and oxidation, enhance glucose oxidation, and increase ketone and branched-chain amino acid oxidation offer hope for improving the prognosis of heart failure patients. However, the regulation of metabolic pathways in heart failure is diverse, while increased glycolysis may increase cardiac workload, it also serves as an energy source in the state of heart failure. The alterations in substrate metabolism during heart failure, whether they result from the disease or represent residual compensatory responses within the body, remain controversial. The debate continues regarding whether the reduction in FAO in heart failure is protective or maladaptive. There is still much work to be done in fully elucidating the changes in energy metabolism signals and their role in heart failure, advancing the development of relevant metabolic therapeutic drugs.

#### Energy in diabetic cardiomyopathy

Diabetic cardiomyopathy is a severe diabetes complication linked to profound cardiac metabolic alterations (Fig. 5b). In diabetic cardiomyopathy, an increase in lipid metabolism and a decrease in glucose metabolism disrupt the fatty acid and glucose balance. This shift elevates the oxygen demand and leads to mitochondrial dysfunction, resulting in cardiomyocyte death and ventricular dysfunction.^477^ Insulin resistance or impaired signaling in diabetes activates FOXO-1 and PDK4, reducing mitochondrial glucose oxidation. FOXO-1 also increases CD36 expression, enhancing lipid uptake and shifting energy reliance from glucose to lipids. This shift increases metabolic stress and dependence on fatty acids, resulting in excessive oxygen use and metabolic imbalance, with the accumulation of lipid intermediates and ROS. ROS are critical factors in the progression of diabetic cardiomyopathy.^478^ ROS accumulation causes lipotoxicity and mitochondrial dysfunction, impairing signaling pathways and leading to myocardial damage and contractile dysfunction. TGR5, a bile acid receptor, may limit fatty acid influx into cardiomyocytes, maintaining energy balance and mitigating diabetic cardiomyopathy risk.^479^

Under normal physiological conditions, insulin signaling through Akt and PKA phosphorylates PFK2, increasing glycolysis and balancing fatty acid and glucose metabolism. In diabetes, impaired insulin signaling decreases glucose uptake. Type 1 diabetes, which is characterized by insulin deficiency, results in reduced cardiac glucose uptake and oxidation, as the heart’s glucose regulation is primarily insulin dependent. In T2DM, hyperglycemia and hyperinsulinemia, coupled with decreased GLUT4 expression,^480^ weaken insulin signaling and diminish glucose metabolism. Without insulin signaling, PFK2 is degraded *via* lysosomes,^481^ further disrupting cardiac energy metabolism. In circulation, Klotho inhibits insulin and insulin-like growth factor-1 (IGF-1) intracellular signaling by blocking the tyrosine phosphorylation of the insulin and IGF-1 receptors.^482^ Mice lacking Klotho exhibit improved glucose tolerance and insulin sensitivity, along with decreased energy expenditure and storage. PPAR-γ plays a pivotal role in glucose and lipid metabolism and overall homeostasis; Klotho, being a target gene of PPAR-γ, suggests its involvement in the partial metabolic regulation mediated by PPAR-γ. Recombinant Klotho can enhance cholesterol efflux by inhibiting the Wnt/β-catenin signaling pathway, thereby reducing lipid accumulation in foam cells.^483^

Despite the increasing depth of research on cardiovascular diseases in recent years, there are still numerous controversies and challenges. The debate on whether energy metabolism overload or deprivation plays a more crucial role in the occurrence and progression of cardiovascular diseases continues. The aberrations in energy metabolism in cardiovascular diseases are influenced by various factors such as genetics, lifestyle, and environmental factors, integrating these elements into metabolic studies poses a challenge. Alterations in energy metabolism in cardiovascular diseases often involve systemic pathophysiological changes across multiple systems, while the key energy metabolic targets (AMPK, SIRT) exhibit functional variances in different tissues, suggesting the need for in-depth mechanistic exploration using tissue-specific animal models and expanding clinical research in the future. Despite advancements in metabolomics, which reveal changes in metabolites, a deeper understanding of the biological mechanisms behind these changes and metabolic regulatory processes is still lacking. Metabolomic studies need to consider the dynamic changes in metabolism during the development of cardiovascular diseases; addressing how comprehensive monitoring and analysis of the metabolome can be achieved at different time points is crucial. Despite the numerous challenges associated with the evolution of multi-omics technologies, the maturation of 3D culture models, and advancements in biosensor technologies, exploring metabolic alterations and regulatory signals under pathological cardiac conditions in vitro holds promise for further elucidating the molecular mechanisms of diseases.

### Energy metabolism and metabolic diseases

#### Energy metabolism in obesity

Obesity, a chronic condition, is fundamentally tied to energy imbalance, where intake surpasses expenditure, leading to excess fat storage. In obesity, disrupted free fatty acid metabolism is vital, with increased synthesis and storage expanding adipose tissue. Although β-oxidation converts fatty acids to energy, this process is often underactive in obese individuals, resulting in insufficient energy release.

Hormones play a pivotal role in energy metabolism regulation in obesity. Obesity is often accompanied by insulin resistance, a state in which insulin signaling is impaired, hindering glucose uptake and utilization. This not only disrupts energy balance but also accelerates fatty acid metabolism, intensifying metabolic disorders in individuals with obesity. Mitochondrial function, which is essential for energy metabolism, is compromised in the skeletal muscle and adipose tissue of obese individuals, contributing to insulin resistance, T2DM, and obesity.^484,485^ In individuals with obesity, elevated glucose and free fatty acid levels directly induce mitochondrial dysfunction.^486^ This dysfunction is driven by ROS, oxidative lipids, endoplasmic reticulum (ER) stress, and genetic predispositions, all of which are worsened by caloric excess.^485^ Oxidative stress damages insulin signaling and incites inflammation, leading to adipocyte dysfunction, whereas inflammation further impairs mitochondrial function in white adipose tissue.^487–489^ Consequently, mitochondrial dysfunction hampers lipid processing and ATP production in adipocytes, contributing to adipocyte hypertrophy.^490^

Leptin, which is secreted by adipose tissue, regulates energy homeostasis, with increased fat reserves typically leading to increased circulating leptin levels.^491^ It influences lipid metabolism not only by regulating food intake but also by reducing triglyceride storage in white adipocytes and the liver, thereby increasing FAO.^492^ Leptin further stimulates FAO in skeletal muscle by activating AMPK, preventing the accumulation of lipotoxic metabolites,^493^ which suppresses appetite and supports efficient energy use. However, in individuals with obesity, this regulatory mechanism is often impaired.^494^ Studies have shown that obese individuals have increased fatty acid uptake in skeletal muscle, yet the ability of leptin to promote FAO is limited, potentially leading to intramuscular triglyceride accumulation.^495^ This metabolic shift suggests potential leptin signaling resistance in obesity, likely contributing to energy metabolism imbalances in muscle tissue.

In obesity, energy metabolism regulation is complex and involves many signaling pathways. The cAMP-dependent protein kinase (PKA) pathway is critical for controlling fatty acid synthesis and breakdown *via* metabolic enzymes such as ACC. Disruption of PKA signaling is linked to altered fat and glucose metabolism in obesity.^496,497^ PGC-1α is essential for mitochondrial biogenesis and function, driving DNA replication and protein expression through TFAM and NRF1. In obesity, decreased PGC-1α activity reduces mitochondrial function, impairing FAO and energy output.^484^ SIRT1 and AMPK are vital for lipid metabolism. SIRT1 promotes mitochondrial function by deacetylating targets such as PGC-1α, whereas AMPK adjusts fatty acid processes according to energy needs. Obesity-induced inflammation decreases SIRT1 and AMPK activity,^498,499^ diminishing the role of PGC-1α and affecting mitochondrial efficiency. SIRT1 enhances insulin secretion through NAD^+^-dependent deacetylation and counteracts inflammatory signals to prevent pancreatic β-cell apoptosis.^500^ Inflammation driven by excess lipids is crucial in metabolic disorders associated with obesity. It triggers the release of cytokines such as TNF-α and IL-6 from macrophages.^501^ These cytokines inhibit insulin signaling, causing insulin resistance,^486,502^ which disrupts fatty acid handling and accelerates obesity.

Epigenetic modifications influence energy metabolism processes in obese patients. Histone deacetylases (HDACs) regulate two main transcription factors, PRDM16 and PGC-1α, through acetylation and deacetylation, playing crucial roles in the pathophysiology of obesity. In mice, the absence of HDAC6 contributes to the onset of obesity.^503^ HDAC3 enhances the expression of PPAR-γ target genes such as adiponectin and AP2, thereby ameliorating obesity.^504^ Inhibiting HDAC5 and HDAC11 can alter adipocyte phenotypes, reducing obesity.^505,506^ Additionally, HDAC5 regulates energy metabolism by affecting BAT activity and UCP1 levels through the inhibition of the hypothalamic STAT5b-TH axis.^507^ HDAC1 influences energy expenditure, obesity, and glucose tolerance by suppressing Pgc1α/Ucp1 transcription.^508^ Inhibiting HDAC8 enhances mitochondrial biosynthesis and function through increased PGC1α, preventing obesity.^509^ HDAC inhibitors improve the intestinal epithelial integrity of obese patients by increasing SCFA levels and activating the Notch signaling pathway.^510^ High methylation levels of FAO -related genes (such as Acaa2, Acsl1, and Cox7a1) are closely associated with abnormal brown fat metabolism.^511^ Enhanced DNA demethylation in the Prdm16 gene promoter may be a significant factor in obesity, and supplementing with α-ketoglutarate can combat obesity in mice.^512^ Folic acid supplementation lowers overall methylation levels by influencing the distribution of differentially methylated regions in adipocytes of obese mice, thereby improving insulin resistance and obesity-related metabolic disruptions.^504^ Recently, substantial progress has been made in understanding the role and mechanisms of epigenetics in regulating thermogenesis in adipose tissue. However, challenges remain: most studies are conducted in vitro or in animals, lacking sufficient clinical trial support for the regulation of adipose tissue thermogenesis, transitioning from laboratory research to clinical applications presents ongoing challenges. Furthermore, different HDAC types have varying effects on obesity regulation, and even within the same class, HDACs exhibit diverse impacts on obesity, necessitating further exploration into the underlying molecular mechanisms.

Adjusting diet and lifestyle is crucial for managing obesity, as excessive energy intake can lead to weight gain. Western high-fat diets (HFDs) can irreversibly disrupt the diversity of the microbiota, disturbing the host’s circadian rhythm and metabolism, thereby promoting obesity.^513^ Compared with the Mediterranean diet and Jiangnan dietary patterns, increasing protein intake in the diet can enhance the abundance of folate-producing bacteria, upregulating folate-mediated one-carbon metabolism and FAO pathways.^514^ Although the ketogenic diet aids in obesity control, it can lead to dyslipidemia in obese mice.^515^ Studies, both in vivo and clinically, have demonstrated that supplementing with prebiotics along with physical exercise can lead to reduced BMI and plasma cholesterol levels in obese subjects and high-fat diet mice, ultimately improving glucose tolerance. Capsaicin regulates fatty acid and glucose metabolism, and its intake promotes thermogenic fat oxidation and aids in weight management.^516^ Currently, intermittent fasting helps improve compliance and represents a promising treatment method for obese patients.^517^

#### Energy metabolism in T2DM patients

In T2DM, energy metabolism is disrupted, which is characterized by chronic hyperglycemia and impaired carbohydrate, fat, and protein metabolism.^518^ A fundamental issue is insulin resistance, which reduces the efficacy of insulin in lowering blood glucose. This resistance is prominent in skeletal muscle, which handles more than 80% of insulin-stimulated glucose uptake. When insulin resistance occurs, glucose uptake and glycogen synthesis in muscle are compromised.^519–521^ This metabolic dysfunction stems from factors such as decreased GLUTs,^522^ diminished insulin-induced ATP production,^523^ and reduced expression of mitochondrial genes.^524,525^ In the liver, insulin suppresses the gluconeogenic enzymes PEPCK and G6 Pase *via* the Akt and FOXO pathways,^526,527^ but resistance impairs this process, increasing glucose output. T2DM progression further weakens β-cell insulin secretion, worsening insulin scarcity. Persistent hyperglycemia catalyzes ROS production through glucose autoxidation and pathways such as the polyol route, as well as AGE formation.^528^ High ROS levels induce oxidative stress and potentially trigger β-cell apoptosis,^529^ undermining insulin secretion and blood sugar control. The hallmark of T2DM is reduced mitochondrial OXPHOS capacity and decreased mitochondrial content in skeletal muscle cells and liver cells. Activation of PGC-1α has been shown to increase mitochondrial OXPHOS capacity, restore mitochondrial ATP production, promote insulin secretion in pancreatic β-cells, enhance insulin sensitivity in skeletal muscle and liver.^530^ Metrnl, an adipokine, has been identified as a key modulator in the insulin signaling pathway, improving impaired insulin responses in myotubes and skeletal muscle through AMPK/PPAR-δ-mediated signaling.^531^ In type 2 diabetic mice, Metrnl has been found to enhance β-cell function by inhibiting β-cell apoptosis and activating β-cell proliferation through the Wnt/β-catenin pathway.^532^ Treatment of C2C12 myotubes with Metrnl increases glucose uptake through the calcium-dependent p38 MAPK and AMPKα2 pathways and regulates the binding of HDAC5 to the GLUT4 promoter in an AMPKα2-dependent manner, promoting GLUT4 transcriptional activation.^533^ These findings underscore the role of Metrnl in regulating energy homeostasis in diabetes.

Epigenetic modifications influence the energy metabolism processes of T2DM patients. T2DM is closely associated with HDACs. HDAC inhibitors promote the differentiation of pancreatic β-cells, increase the levels of glycolytic and gluconeogenic enzymes, and improve insulin resistance.^534^ HDAC-3 promotes FOXO1 deacetylation leading to insulin resistance in T2DM.^535^ HDAC-3 negatively regulates PPAR-γ signaling, causing signaling disruption, altering glucose homeostasis, and increasing hepatic glucose and lipid metabolism.^536^ SREBP1 recruits HDAC-8 to activate the Wnt signaling pathway resulting in insulin resistance and hyperglycemia.^537^ HDAC-1 recruited by ATF6 inhibits SREBP2-mediated gene transcription, contributing to glucose homeostasis.^538^ Activation of CaMK signaling leads to the release of HDAC-5, which in turn activates GLUT4 in skeletal muscle to stimulate glucose uptake; any dysregulation can lead to insulin resistance.^539^ The intestinal microbiota composition differs in T2DM patients, and these microbial changes may lead to decreased methylation of CpG sites in the TLR4 exon and TLR2 promoter, affecting genetic epigenetic regulation.^540^ Currently, HDAC inhibitors are primarily used in clinical cancer treatment. Although laboratory studies suggest a potential role in alleviating diabetes, the development and translation of HDAC inhibitors for diabetes therapy are still in the early stages, highlighting the importance of future research in unraveling their mechanisms, optimizing drug delivery processes, and evaluating long-term safety through clinical trials to assess their efficacy in combating T2DM effectively.

Pharmacological strategies for treating T2DM aim to increase insulin effectiveness and secretion. These include oral hypoglycemic agents and injectables such as insulin and GLP-1 receptor agonists, which are designed to improve insulin sensitivity or supplement deficient insulin secretion. Among oral options, metformin is popular because of its antihyperglycemic effects. Metformin primarily reduces hepatic gluconeogenesis, thereby lowering glucose output.^541^ It also modestly enhances the skeletal muscle insulin response, increasing glucose uptake.^542^ Metformin acts by activating insulin receptors and IRS-2, increasing the activity of GLUTs, such as GLUT1, which facilitates glucose uptake.^543^ This action intensifies the inhibitory effect of insulin on gluconeogenesis.^544^ Additionally, metformin increases glucose uptake in muscle by activating insulin receptor tyrosine kinase, partly by promoting GLUT4 translocation to the cell membrane.^545^

The efficacy of metformin largely arises from the activation of AMPK, which is pivotal for maintaining the energy equilibrium of the cell. The activation of AMPK increases insulin receptor and IRS-2 activity, facilitating GLUT movement to the cell surface and thereby increasing glucose uptake.^543^ Furthermore, AMPK is vital for regulating lipid metabolism by curbing fatty acid synthesis and enhancing β-oxidation, which impacts metabolic balance.^546,547^ This action reduces the amount of intracellular free fatty acids that hinder insulin signaling and glucose transport^548^ and can impair insulin secretion in β-cells.^549^ Additionally, β-oxidation byproducts such as acetyl-CoA and citrate can inhibit glycolytic enzymes.^550^ Thus, metformin helps lower glucose and free fatty acids in the blood, preventing lipid buildup in insulin-sensitive tissues and enhancing insulin secretion and sensitivity.

Restoring mitochondrial functionality is becoming a strategic focus in T2DM treatment. By targeting ETC activity, certain compounds can increase mitochondrial energy output, improving metabolic states in T2DM patients. Imeglimin, a novel antidiabetic agent, positively impacts organs such as skeletal muscle, the liver, and the pancreas by restoring mitochondrial function.^518^ Mitochondrial biogenesis, which involves the renewal and replication of mitochondria, is vital for cellular energy. SIRT1, an NAD^+^-dependent deacetylase, activates PGC-1α to promote this process.^551^ Increased SIRT1 activity increases mitochondrial function and insulin sensitivity, which are crucial for better metabolic control in T2DM patients. Mitochondria are primary sources of ROS, which can lead to damage if they accumulate excessively. Antioxidants and radical scavengers help reduce oxidative damage, safeguarding mitochondrial integrity and ensuring proper function. In addition to pharmacological control, recent studies have revealed that, owing to the pathogenic role of mitochondrial abnormalities in diabetes, transferring healthy mitochondria to pancreatic β-cells to increase their insulin secretion function has emerged as a therapeutic strategy^552^ and has garnered significant attention. However, several key challenges persist, with mitochondrial storage remaining a hurdle.^553^ Isolated mitochondria can maintain activity on ice for 1–2 h, but the efficiency of mitochondrial transplantation is often suboptimal. Although various methods have been developed to increase transplantation efficiency, mitochondrial function is still affected. Mitochondrial DNA can be inherited through cytoplasmic genetics, raising safety concerns. The use of cross-species mitochondrial transplantation has raised concerns regarding the immune response. Future research needs to address these issues further to facilitate the clinical application of these therapies.

Physical activity combined with dietary interventions may represent a promising strategy for improving metabolic disorders. High-sugar diets can lead to insulin resistance and disrupted glucose metabolism. Among individuals with metabolic syndrome, interventions combining exercise training with dietary supplementation of polyunsaturated fatty acids have shown significant improvements in insulin sensitivity, serum C-reactive protein levels, and high-density lipoprotein concentrations.^554^ Dietary fiber can promote the growth of acetate- and butyrate-producing bacteria; increased production of short-chain fatty acids (SCFAs) can stimulate glucagon-like peptide 1 (GLP-1) secretion, thereby enhancing insulin secretion, improving glucose homeostasis, reducing inflammation, and alleviating T2DM.^555^ Due to its high content of unsaturated fatty acids, fiber, and antioxidants, the Mediterranean diet has a significant positive impact on the treatment of T2DM, improving the release of inflammatory factors and insulin resistance.^556^ However, further research is needed to elucidate the potential molecular mechanisms underlying how dietary components impact insulin sensitivity to better understand their effects. Intermittent fasting helps T2DM patients break down and utilize endogenous fats, regulating blood glucose levels, and thus enabling the body to regain energy balance.^557^

In metabolic disorders, magnesium deficiency is prevalent in individuals with obesity, diabetes, or metabolic syndrome, with magnesium supplementation shown to improve insulin sensitivity and blood glucose control in T2DM patients.^558,559^ Magnesium acts as a cofactor for enzymes like creatine kinase, adenylate cyclase, and Na^+^-K^+^-ATPase, directly participating in blood glucose regulation. Additionally, magnesium enhances insulin sensitivity, ameliorates insulin resistance and glucose metabolism, mediates beta cell insulin release, and modulates intracellular signal transduction downstream of the insulin receptor. However, hyperinsulinemia and insulin resistance in diabetic patients can impair insulin-stimulated cellular uptake of magnesium, leading to decreased magnesium levels in the body. Elevated blood glucose levels increase insulin secretion, affecting the transport of Na^+^ and K^+^, and influencing cellular energy metabolism status.

In metabolic disorders, the intricate interplay within the gut–brain–adipose axis, which includes neural and endocrine regulation, has garnered substantial attention. The influence of the gut microbiota on energy metabolism is highly important and warrants further exploration in future research directions. Given the complexity of pathogenic mechanisms involving multiple organs and systems, current studies face challenges in pinpointing singular therapeutic targets, posing a significant hurdle to drug development. Future endeavors should concentrate on merging advancements in multi-omics technologies to monitor interindividual metabolic variabilities, emphasizing early diagnosis and personalized therapeutic strategies.

### Energy metabolism in autoimmune diseases

**Fig. 6:**
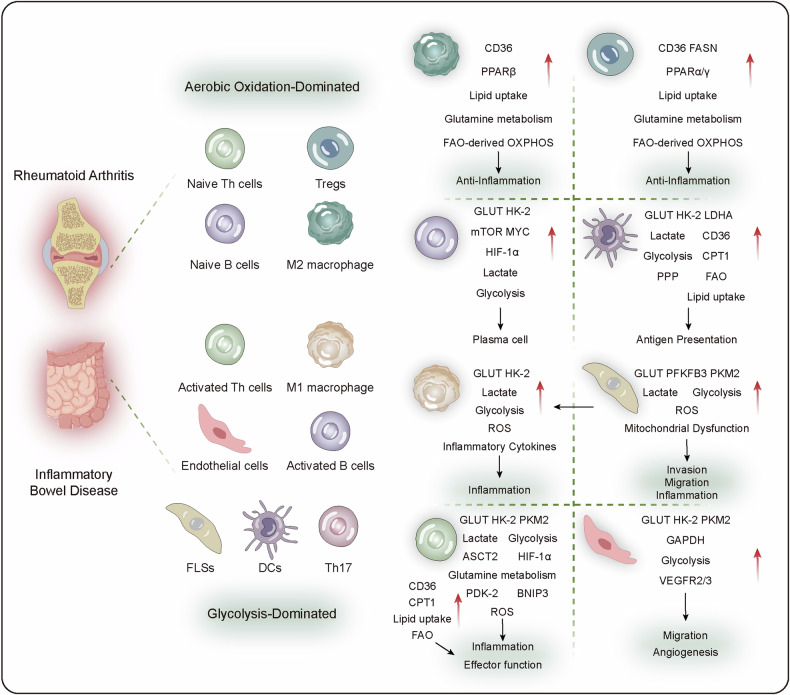
Energy metabolism in autoimmune diseases. In autoimmune diseases, metabolic abnormalities in immune cells are a key factor. In some situations, CD4^+^ T cells enhance glycolysis and mitochondrial OXPHOS, while in rheumatoid arthritis (RA), T cells suffer from impaired mitochondrial OXPHOS, turning to the pentose phosphate pathway. Naive Th cells and B cells tend to undergo aerobic oxidation, while activated Th cells and B cells tend to undergo glycolysis. Treg cells can utilize glycolysis and lactate to maintain their functions in the TME. M1 macrophages tend to have aerobic glycolysis, while M2 macrophages rely more on OXPHOS. FLSs undergo metabolic reprogramming, shifting towards enhanced glycolysis. As antigen-presenting cells, DCs significantly alter their metabolic pathways, such as OXPHOS and glycolysis, during activation. These changes in energy metabolism promote the abnormal proliferation of fibers and blood vessels and exacerbate the inflammatory process. DC dendritic cells, FLS fibroblast-like synoviocytes, Treg regulatory T cells, MDSC myeloid-derived suppressor cells

Autoimmune diseases arise when the immune system mistakenly attacks the body’s own tissues, producing pathological reactions to self-antigens. Altered energy metabolism plays a crucial role in these conditions, particularly affecting the activation, differentiation, and function of macrophages, T cells, and B cells. For example, metabolic reprogramming in T and B cells during autoimmune diseases results in heightened activation and distinct energy profiles compared with those of normal cells.^560^ Rheumatoid arthritis (RA) is a chronic autoimmune disorder characterized by inflammation of the synovial tissue and the presence of autoantibodies.^561^ Typically, the synovium is infiltrated by T cells, B cells, and macrophages, triggering a damaging response in fibroblast-like synoviocytes (FLSs), leading to excessive proliferation and destruction of cartilage and bone.^562^ Inflammatory bowel disease (IBD), which includes Crohn’s disease and ulcerative colitis, is characterized by chronic relapsing inflammation and epithelial injury in the gastrointestinal tract.^563^ Systemic lupus erythematosus (SLE) is an autoimmune disorder involving multiple organ systems and is characterized by clinical and serological heterogeneity and dysregulated interferon responses.^564^ Although the specific etiology of these diseases remains unclear, altered energy metabolism and the resulting immune dysregulation are believed to significantly contribute to their development and persistence. In the early stages, the body is in an inflammatory state, leading to an increase in metabolic rate to support the energy required for inflammation and immune responses. During this time, activation of the glucose metabolism pathways is typically observed. As the disease progresses, the body may experience impaired mitochondrial function, increased production of thiol proteins and free radicals, leading to disruptions in energy metabolism. With the continued development of the disease, exacerbation of inflammation further affects energy metabolism. Some autoimmune diseases such as RA may lead to joint destruction and tissue damage, consuming more energy to meet the demands of repair and recovery. Understanding these dynamic changes in energy metabolism is crucial for initiating early treatment or preventive measures during the window period of autoimmune diseases (Fig. 6).

Autoimmune disease-related autoinflammatory responses require significant amounts of energy, leading to increased metabolism of fats, glucose, and glutamine and a shift from OXPHOS to glycolysis. Inflammation linked to autoimmunity involves diverse immune cell subsets within disease-specific environments, each with unique metabolic needs.^565^ Before encountering antigens, naive Th cells rely primarily on FAO and OXPHOS for energy. In contrast, activated Th cells depend more on glycolysis, increasing glucose uptake *via* transporters to support proliferation. Enhanced glucose uptake and glycolysis are critical in Th17 cell differentiation.^566,567^ Effector T cells rely on glycolytic metabolism for growth and function, whereas regulatory T cells (Tregs) use lipids through mitochondrial β-oxidation and generate ATP *via* OXPHOS.^568^ In various autoimmune diseases, proinflammatory T cells have higher lipid contents than healthy individuals do.^569^ T cells utilize lipids for membrane biosynthesis, cell division, and migration rather than energy production through β-oxidation.^570,571^ Fatty acid and cholesterol biosynthesis mediate Th17 cell formation, whereas FAO supports Treg differentiation.^572^ Amino acid metabolism, particularly glutaminolysis, is pivotal in Th17 cell development.^573^ B cells also exhibit altered energy metabolism under autoimmune conditions. Naïve B cells maintain a low metabolic state, whereas their activation relies on OXPHOS-driven metabolic programming.^574^ Autoimmune diseases feature diverse macrophage subsets with various functions.^575^ Generally, inflammatory macrophages depend on glucose as an energy source, whereas anti-inflammatory macrophages require less glucose and rely on mitochondrial OXPHOS for their bioenergetic and biosynthetic demands.^576^ Specifically, M1 macrophages utilize glycolysis, whereas anti-inflammatory M2 macrophages typically rely on β-oxidation.^577^

#### Glycolysis

In autoimmune diseases, the upregulation of GLUT1, which facilitates glucose transport, has been reported.^578^ Key glycolytic enzymes, such as HK, PKM2, GAPDH, and LDHA, exhibit increased activity in inflamed joints.^579,580^ HK catalyzes the initial step in glucose metabolism, enhancing the migration and invasion capabilities of FLSs.^579^ Elevated HK2 levels in Th17 cells, dendritic cells, and FLSs promote glycolysis. This enhanced glycolysis results in high lactate and low glucose concentrations in inflamed joints,^581^ disrupting immune cell balance. Specific inhibition of HK2 with 3-bromopyruvate reduces joint swelling and histological damage in SKG mice, a RA model, by suppressing glycolysis-dependent dendritic cell activation and Th17/Treg imbalance.^582^ Overactive HK2 promotes synovial cell proliferation and secretion by mediating AMPK activation of NF-κB signaling.^583^ Inhibitors of HK2 effectively suppress the production of inflammatory factors.^584^

Studies have shown that increased glucose uptake and glycolysis in the lymph nodes and thymus of patients with autoimmune diseases such as RA are correlated with disease severity and therapeutic response.^585^ Inadequate vascularization often creates hypoxic conditions in local tissues, which play crucial regulatory roles in cellular processes.^586^ Hypoxia can extensively alter mitochondrial structure, dynamics, and mtDNA stability, leading to impaired respiration, excessive ROS production, increased oxidative damage, and reduced ATP production.^587^ Driven by the hypoxic joint environment, Tregs can quickly transform into pathogenic Th17 cells,^588^ exacerbating inflammation.

Under normal oxygen conditions, HIF-1α is degraded, but under hypoxia, it accumulates and promotes the expression of glycolytic enzymes. HIF-1α acts as a key regulator of the anaerobic metabolic switch, enhancing processes that increase the production of glycolytic energy, including GLUTs and glycolytic enzymes.^589^ It also induces synovial fibroblasts to produce proinflammatory cytokines such as IL-1β, IL-6, IL-8, and TNF-α and cell adhesion molecules such as VCAM-1, TSP1, and CXCL12, thereby promoting inflammation.^590^ HIF-1α increases glycolytic flux and glucose consumption to produce energy and synthesize biosynthetic precursors.^591^ By deriving more energy from anaerobic glycolysis *via* HIF-1α, in addition to generating biomass, cells become inflammatory effectors.^586,592^ Mitochondrial dysfunction in T cells causes increased glucose flow into the pentose phosphate pathway, a process that is exacerbated under hypoxic synovial conditions.

The activation of the PI3K/Akt/mTOR signaling pathway is crucial during immune cell activation. CD28 costimulation triggers the PI3K/Akt axis in T cells, inducing GLUT1 expression, which enhances glucose uptake and utilization.^593^ This metabolic shift provides the energy and biosynthetic precursors necessary for T-cell proliferation and activation. mTORC1 and mTORC2 play distinct but complementary roles in regulating T and B-cell metabolism. Activated by the PI3K/Akt pathway, mTORC1 is closely linked to the expansion of proinflammatory T cells, supporting glycolysis and differentiation into Th1 and Th17 cells while limiting their differentiation into Treg cells.^593^ In contrast, mTORC2 is associated with cell survival and metabolic homeostasis.^560^ In SLE patients, B cells exhibit increased mTORC1 activity, which is correlated with increased lactate production, indicating a metabolic shift toward glycolysis.^593^

Owing to inflammation, macrophages, T cells, B cells, and stromal cells within tissues remain activated and under high metabolic stress, creating a microenvironment low in oxygen and glucose but rich in metabolic intermediates such as lactate. Lactate regulates T-cell differentiation, a process critical for sustaining chronic inflammation.^594,595^ It reprograms T cells, promoting proinflammatory Th17 phenotypes and aggressive CD8^+^ T-cell transformation.^596,597^ Th17 cells, a subset of helper T cells, secrete IL-17, which plays a central role in driving inflammation and autoimmune disease progression. Research indicates that lactate enhances IL-17 production through lactate-dependent metabolic pathways in CD4^+^ T cells, involving the activation of PKM2 and STAT3, which together facilitate Th17 differentiation and functionality.^598^ Tissue-resident T cells exhibit metabolic profiles similar to those of proinflammatory macrophages, which utilize nuclear PKM2/STAT3 signaling to maintain persistent IL-1 and IL-6 production.^582^ These findings suggest that lactate not only affects T-cell differentiation but also may modulate T-cell effector functions, particularly in tissue microenvironments. Furthermore, tissue lactate levels impact the migration of CD4^+^ and CD8^+^ effector T cells. The accumulation of extracellular lactate and its metabolites can inhibit T-cell motility, potentially limiting their infiltration into sites of inflammation and modulating immune responses.^582^ By sensing tissue lactate, synovial T cells become immobilized and trapped within the tissue microenvironment.^599^ These findings highlight the multifaceted role of lactate in immune cell behavior.

#### OXPHOS

OXPHOS, as a critical energy supply mechanism, involves metabolic reprogramming in autoimmune diseases. In patients with SLE and experimental animal models, CD4^+^ T cells exhibit increased mTOR signaling activation alongside increased glycolysis and mitochondrial OXPHOS. Pathogenic Th17 cells exhibit increased aerobic glycolysis and TCA activity, as suggested by single-cell RNA sequencing data, which can be suppressed through inhibition of CaMK4.^578^ Blocking the ETC and inhibiting OXPHOS can disrupt the effector functions of Th17 cells at sites of colitis inflammation, indicating that the secretion of cytokines by Th17 cells relies on ETC-mediated OXPHOS to induce inflammation in IBD and psoriasis models.^600^ Untreated RA patients show significant changes in OXPHOS transcriptional regulation in CD8^+^ TEM cells.^601^ Furthermore, Treg cells have been demonstrated to rely on OXPHOS for survival and functionality, serving as crucial regulatory cells in autoimmune diseases.

#### FA metabolism

Aberrant fatty acid metabolism exerts significant influence on the activation of immune cells, including Treg cells, naïve Th cells, and anti-inflammatory macrophages. Unlike effector T cells, Treg cells predominantly rely on FAO for energy production. Foxp3 suppresses Myc and glycolysis while enhancing OXPHOS, enabling Treg cells to survive in low-glucose environments.^602^ Alterations in fatty acid levels or composition can impact Treg function, potentially leading to immune dysregulation in autoimmune diseases.^603^ In RA, cells exhibit mitochondrial dysfunction and enhanced fatty acid metabolism, contributing to tissue invasiveness and pro-inflammatory characteristics. In an IBD mouse model, the upregulation of serum butyric acid, which enhances CPT1A activity, is considered a crucial pathway for reactivating FAO and subsequently inducing Treg differentiation.^604^ The activation of FAO and Treg cell differentiation through AhR activation alleviateS colitis and arthritic symptoms in mice.^605^ There is a potential association between abnormal fatty acid metabolism and the pathogenic activation of Th cells in autoimmune diseases. During Th17 cell differentiation, fatty acid synthesis is significantly upregulated. TRM cells play a central role in arthritis recurrence, resulting in increased fatty acid consumption and CCL5 production, aiding in recruiting immune effector cells during arthritis flare-ups.^606^ In RA patients, CD8^+^ TEM and CD8^+^ T cells show increased fatty acid uptake, characterized by the upregulation of fatty acid transport and sensing-related genes (FABP and CD36) as well as FAO-related genes (ACADVL).^607^ Studies suggest that inhibiting lipid metabolism can suppress the effector functions of CD8^+^ T cells in RA.^606^ However, the metabolic reprogramming of TRM cells in autoimmune diseases remains unclear.

#### Amino acid metabolism

The glutamine supply is essential for immune cell proliferation and is a key driver of angiogenesis.^608^ Amino acid transporters such as SLC1A5 enable efficient glutamine uptake in T cells, which is crucial for responding to T-cell receptor and CD28 costimulation signals. The activity of SLC1A5 links these signals to the mTORC1 signaling cascade, promoting T-cell expansion and differentiation into effector Th1 and Th17 cells, which are central to RA pathogenesis.^609^ When glutamine is limited, T-cell proliferation and cytokine secretion are inhibited, potentially shifting differentiation toward a Treg cell phenotype.^610,611^ Deficiency in glutamine inhibits the activation of mTORC1, subsequently downregulating NKT cell proliferation through mTORC1-c-Myc signaling.^612^ Elevated levels of glutamine metabolism have been observed in splenic mononuclear cells of MRL/lpr mice prone to lupus as well as in peripheral blood mononuclear cells of individuals with SLE, promoting differentiation of Th17 cells.^613^ The promotion of Th1, Th17 functions, and cytokine secretion play crucial roles in the pathogenesis of Crohn’s disease.^614^ Conversely, arginine deficiency promotes ATF4-mediated SLC7A11 transcription and Treg cell differentiation.^615^ Recent studies suggest that glutamine metabolism may control Th cell differentiation through epigenetic regulation. Aberrant activation of GLS-mediated glutamine breakdown triggers H3K9Ac and H3K27Ac epigenetic modifications within the IL17A gene promoter region of Th17 cells, enhancing chromatin accessibility of RORC, thereby exacerbating IL-17A expression.^616^ Blocking GLS1 can promote Th2 cell differentiation, suppress Th17 cell differentiation *via* mTORC1 pathway inactivation, while maintaining Th1 cell differentiation unaffected.^617^ The metabolite α-KG from glutamine breakdown has been identified as a crucial player involved in epigenetic reshaping mediated by glutamine breakdown, acting as a co-factor for peroxidases and regulating histone and DNA methylation levels in glutamine breakdown-mediated epigenetic reshaping.^618^ Currently, alterations in amino acid metabolism are considered potential biomarkers and therapeutic targets for autoimmune diseases. However, the complexity of regulatory mechanisms due to multiple metabolic changes poses challenges in interpreting and validating these metabolic alterations.

#### Interplay between epigenetic modifications and energy metabolism

Immune cells undergo energy metabolism reprogramming upon activation, and metabolic‒epigenetic changes play crucial roles in autoimmune diseases. The conversion of glutamine to α-KG, a substrate for the TCA cycle intermediate and histone demethylases, can influence T-cell differentiation by regulating chromatin accessibility.^619^ Overall, DNA methylation levels decrease in SLE patients, and disease activity and progression in lupus patients are negatively correlated with methylation patterns,^620,621^ primarily due to the reduced expression/activity of DNMTs.^622^ Global hypomethylation in RA may lead to immune dysregulation.^623^ Treg cells in RA patients exhibit an aberrant DNA methylation pattern, particularly in the promoter region of the CTLA-4 gene. Elevated methylation in this region reduces CTLA-4 expression, causing dysfunctional Tregs to be unable to activate immune regulatory pathways.^624^ The MEK-ERK kinase pathway primarily regulates methylation, and the inhibition of any protein in the ERK pathway could ultimately lead to the downregulation of DNMTs. T cells from active SLE patients show reduced phosphorylation of all three signaling molecules: ERK, MEK, and RAF.^625^ Intermediate products of energy metabolism, such as acetyl-CoA, are mediated by LDHA and promote histone acetylation, enhancing translation of Ifng during Th1 differentiation.^111^ GLUT3 is highly expressed in Th17 cells and converts citrate in the cytoplasm to acetyl-CoA, promoting histone acetylation around the Il-17 gene.^626^ SCFA butyrate inhibits histone deacetylases, promoting high histone acetylation in the promoter region of follicular regulatory T cell (Tfr) signature genes, leading to Tfr expansion;^627^ in RA patients, decreased butyrate levels may lead to Tfr suppression and synovial inflammation.^628^ Aberrant histone acetylation has been observed in MRL/lpr mice prone to lupus, where increased HDAC9 activity is noted; thus, defects in HDAC9 in MRL/lpr mice can mitigate autoimmune responses.^629^ Disruption of HAT/HDAC in the synovial tissue of RA patients ultimately leads to an imbalance in histone acetylation and deacetylation.^630^ HDAC inhibitors can serve as a therapeutic strategy to control pathological conditions characterized by functional expansion of Th1 and Th17 cells.^622^ The future challenge lies in discovering new, safe immunomodulatory drugs that act specifically on epigenetics, as epigenetic modifications are highly subject to variations influenced by micronutrients, diet, and/or physical activity.

Reprogramming of energy metabolism in autoimmune diseases serves as both a pathogenic factor and a potential therapeutic target. The conceptual framework of disease classification, intervention, and biomarker discovery suggests that reprogramming metabolism through epigenetics could be a novel strategy for developing protection against inflammation in autoimmune diseases. Furthermore, dietary management, recognized as a promising source for the accumulation or elimination of metabolic substrates, has been identified as a promising origin for new drug targets. Current research has focused primarily on key metabolic pathways and molecules such as mTOR, AMPK, and HIF-1α, with many targets for metabolic therapy under early stages of investigation in preclinical studies or clinical trials. However, the translation from research to clinical application has a long journey. Future studies should delve deeper into the potential mechanisms of immune cell metabolic dysregulation and identify potential therapeutic targets, with particular attention to the limited research on tissue-resident memory T (TRM) cells and their regulation in diseases such as RA, SLE, and IBD.

### Energy metabolism in cancer

During cancer initiation and progression, tumor cells develop a distinct and aberrant metabolic phenotype known as metabolic reprogramming. This specialized energy metabolism encompasses several intracellular pathways, prominently featuring increased glycolysis, increased glutamine metabolism, alterations in the TCA cycle and OXPHOS, and aberrant FAO. Collectively, these metabolic adaptations form the foundation of metabolic reprogramming in tumor cells, which not only drives their rapid proliferation and metastatic potential but also significantly impacts their stemness, plasticity, and other critical biological properties.^27,631–635^ During the early stages of tumor growth, metabolic activity of tumor cells increases to support their rapid growth and proliferation demands. At this stage, tumor cells tend to generate energy through the glycolytic pathway, known as “aerobic” metabolism. As the tumor progresses, tumor cells may enhance glucose uptake and utilization while also relying on other metabolic pathways for energy production, such as fatty acid oxidation and protein metabolism. This mixed mode of “aerobic” and “anaerobic” metabolism may help tumor cells adapt to various microenvironmental conditions, such as low oxygen levels and nutrient deficiencies. With tumor growth and metastasis, internal tumor tissues might experience uneven blood supply and oxygen distribution, leading to localized hypoxia. This hypoxic environment can induce tumor cells to adjust their metabolic pathways, favoring a more anaerobic-dependent energy production method, facilitating tumor growth and invasion.

#### The impact of alterations in energy metabolism on tumor proliferation, stemness, and other facets of tumor cell biology

##### Glycolysis

**Fig. 7:**
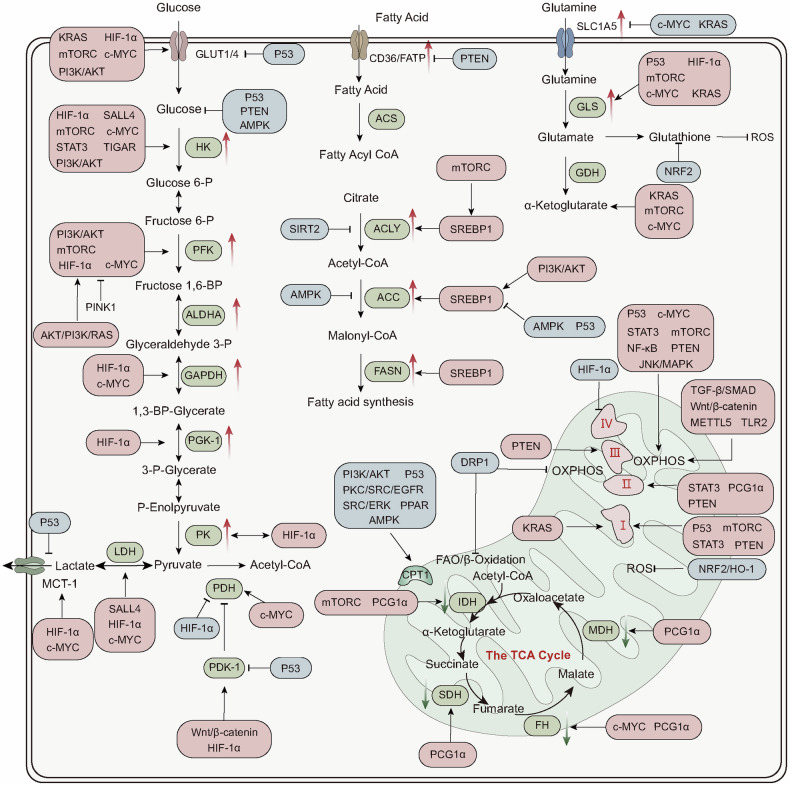
Energy metabolism in cancer. Cancer cells undergo metabolic reprogramming in their energy metabolism, characterized by enhanced glycolysis, glutamine metabolism, and FAO, but the TCA cycle and OXPHOS are suppressed. The activity of glycolysis-related transport proteins and enzymes such as GLUT, HK, PFK, PK, and LDH is increased. The activity of glutamine transporters and GLS is increased, catalyzing the production of glutamate for biosynthesis or energy synthesis. The expression of fatty acid transport proteins (CD36) and synthetic proteins (ACLY, ACC, FASN) is increased. However, the activity of key enzymes in the TCA cycle, such as IDH, SDH, FH, and MDH, is suppressed. A variety of signaling molecules undergo changes in expression during this process and regulate the energy metabolism of cancer cells. Overall, HIF1-α, KRAS, SALL4, c-MYC, PI3K/AKT, and mTOR, among others, play pro-oncogenic roles mainly by promoting glycolysis and glutaminolysis while inhibiting the TCA cycle and oxidative phosphorylation. P53, PTEN, AMPK, NRF2, PCG1α, and others play tumor-suppressive roles, inhibiting glycolysis and fatty acid transport and synthesis while promoting the mitochondrial TCA cycle and oxidative phosphorylation processes (red boxes: promoting signaling molecules; blue boxes: inhibitory signaling molecules). HK hexokinase, PFK phosphofructokinase, PK pyruvate kinase, ALDHO aldehyde dehydrogenase, GAPDH glyceraldehyde-3-phosphate dehydrogenase, PGK1 phosphoglycerate kinase 1, LDH lactate dehydrogenase, PDH pyruvate dehydrogenase, PDK1 pyruvate dehydrogenase kinase 1, ACLY ATP citrate lyase, ACC acetyl-CoA carboxylase, FASN fatty acid synthase, ACS acetyl-CoA synthase, GLS glutaminase, GDH glutamate dehydrogenase, IDH isocitrate dehydrogenase, SDH succinate dehydrogenase, FH fumarate hydratase, MDH malate dehydrogenase, CPT1 carnitine palmitoyltransferase 1

In various cancers, dysregulated energy metabolism, particularly the activation of glycolysis, is a prominent characteristic. This phenomenon, known as the Warburg effect,^636^ indicates that cancer cells preferentially utilize glycolysis instead of OXPHOS to meet their energy demands, even under aerobic conditions (Fig. 7). This metabolic shift is crucial for the growth and maintenance of cancer cells, especially in tumor stem cells (CSCs) or cells with stem-like properties, which often exhibit increased glycolytic activity.^637,638^

Compared with physiological aerobic oxidation, glycolytic alterations in tumor cells promote crucial biological processes such as proliferation, stemness, and metastasis. First, glycolysis provides a swift method for ATP production, potentially surpassing the traditional efficiency of the TCA cycle.^639^ This pathway also endows tumor cells with abundant precursors,^640,641^ which are essential for the biosynthesis of macromolecules such as lipids, nucleic acids, and proteins, thereby supporting rapid tumor growth and proliferation and inducing mesenchymal transformation.^642^ Additionally, lactate secretion during glycolysis lowers the pH of the TME,^643^ increasing invasive capacity.^644^ This local acidification increases the expression of vascular endothelial growth factor A, further promoting cancer cell proliferation^645^ and modifying other metabolic pathways.^646,647^ Furthermore, the preferential conversion of pyruvate to lactate not only reduces oxidative stress in mitochondria but also regenerates NAD^+^ from NADH, helping to alleviate the electron load and preventing disruption of the ETC caused by excess electrons.^27,36^

In tumor cells, key enzymes and transport proteins within the glycolytic pathway, including GLUTs, HK2, PFK1, and PKM2, are markedly overexpressed.^648^ This upregulation enhances glycolytic activity, providing the essential energy and biosynthetic precursors required for the rapid growth and proliferation of tumors.^642^ In contrast to that in normal cells, the increased expression of GLUTs in cancer cells leads to increased glucose uptake.^649^ HK2 is consistently upregulated across various cancers, driving the glycolytic process.^650–653^ Similarly, ALDOA is overexpressed in numerous tumors and is linked to cytoskeletal integrity and epithelial‒mesenchymal transition (EMT).^654^ PKM2 is particularly pivotal in metabolic reprogramming, modulating the ATP production necessary for tumor cell proliferation and activating the HIF-1 transcription factor, which further encourages glycolytic metabolism.^655^ In addition to its role in glycolysis and ATP production, PKM2 also regulates the redirection of glycolytic intermediates to the pentose phosphate pathway, thereby generating NADPH, which is critical for cellular redox homeostasis,^656^ thus shielding cancer cells from oxidative stress.^657^

The glycolytic process in tumor cells is intricately regulated by various signaling molecules. HIF-1α is pivotal in this regulation, promoting the glycolytic pathway by activating the transcription of genes encoding GLUTs and glycolytic enzymes.^658,659^ Under normal conditions, HIF-1α stability and activity are tightly controlled by oxygen levels^660^ and inhibited by PINK1,^661^ making it rarely detectable.^662^ However, in tumor cells, PINK1 is often deficient,^661^ and HIF-1α can be activated through nonhypoxic mechanisms even in oxygen-rich conditions.^663^ This is partly due to mutations in the von Hippel‒Lindau protein in some aggressive tumors, which impairs the degradation of HIF-1α. Additionally, oncogenes such as Akt, PI3K, and Ras can induce the nonhypoxic expression of HIF-1α.^663^ When metastatic cells enter the circulatory system, they encounter a more intense oxygen environment, potentially increasing oxidative stress. To counter this, circulating tumor cells (CTCs) exhibit unique adaptations: they tend to cluster together, forming cell aggregates.^664^ This clustering creates a hypoxic core where HIF-1α accumulates, thereby providing a survival advantage under high oxidative stress.^665^ HIF-1α enhances the expression of the GLUTs GLUT1, LDH, PKM2, and PDH kinase (PDK1).^666,667^ PDK1 inhibits the activity of the PDH complex through phosphorylation, reducing the oxidation of pyruvate to acetyl-CoA in the Krebs cycle and increasing lactate production.^668,669^ Furthermore, glycolytic enzymes themselves can influence HIF-1α; for example, PKM2 activates the HIF-1 transcription factor by modulating ATP levels required for tumor cell proliferation, thereby promoting glycolysis.^655^ pSTAT3 enhances the expression of HIF-1, GLUT1, and HK2, thereby increasing glucose consumption and lactate production, likely through the upregulation of HIF-1-mediated glycolytic genes. PGC-1α is overexpressed in many tumors, increasing GLUT4 and HK2 protein levels.^670^ Additionally, the overexpression of mTORC1 in numerous tumors increases GLUT1 and PFK1 mRNA levels and glucose consumption. The mTOR-induced activation of glycolysis is also associated with HIF-1 activation.^188^

The PI3K/Akt signaling pathway is crucial for regulating key cellular processes in cancer, including cell survival, proliferation, angiogenesis, and metabolic reprogramming.^671,672^ Upon activation, Akt enhances glucose uptake in cancer cells by promoting the surface localization of GLUT1 and inhibiting its internalization.^666^ Additionally, Akt stimulates mTORC1, which upregulates HK2 expression.^673^ Through increasing GLUT1 and PFK1 levels, mTORC1 facilitates increased glucose uptake and glycolysis, which is significantly tied to HIF-1α activation.^188^ Several other molecular players also modulate this metabolic shift. Krüppel-like factor 8 (KLF8) and insulin gene enhancer protein 1 (ISL1) increase GLUT4 expression.^674,675^ The stem cell factor SALL4 augments the expression of HK2 and LDH.^676^ The oncogene c-Myc directly upregulates a suite of glycolytic genes (HK2, PFK1, TPI, GAPDH, ENO, LDHA, and MCT1), intensifies glycolytic flux in cancer cells,^663,677,678^ and stabilizes HIF-1α under normoxic conditions by inhibiting its proteasomal degradation through interaction with the von Hippel–Lindau protein.^679^ KRAS, a prominent oncogene frequently mutated in cancers, characteristically leads to decreased OXPHOS, elevated glycolysis, and increased ROS production.^680,681^ This effect is mediated through the Raf/MAPK/ERK/c-Myc and PI3K/Akt pathways, which increase GLUT1 and HK expression.^682^ PTEN serves as a metabolic gatekeeper by inhibiting HK2 expression *via* inactivation of the Akt/mTORC1/4E-BP1 signaling pathways.^683^ Consequently, the loss of PTEN can reprogram glucose metabolism in cancer cells. Elevated PTEN levels, on the other hand, diminish glucose uptake while promoting mitochondrial biogenesis and multidrug resistance.^684^

The tumor suppressor p53 counteracts the Warburg effect and favors mitochondrial OXPHOS through multiple sophisticated mechanisms. A notable mechanism involves the direct interaction between p53 and HIF-1, where p53 sequesters HIF-1, thereby abrogating its metabolic functions. Under normoxic conditions, p53 directly limits glucose uptake by repressing the transcription of GLUT1 and GLUT4, and induces TIGAR expression, thereby reducing PFK1 activity.^685^ In hypoxic environments, p53 overexpression leads to an increase in glycolytic proteins (GLUT1 and GLUT3) while keeping HK2 levels unchanged, thereby markedly reducing glycolytic flux.^686^ When p53 is mutated, increased glycolysis is associated with increased protein levels of GLUT1, GLUT3, HK1, and HK2. The nutrient-deprived state of tumor cells can directly activate AMPK, a critical cellular energy sensor that helps restore energy homeostasis.^687^ AMPK, in turn, stabilizes and activates p53, creating a positive feedback loop that enhances its tumor-suppressive functions.^666^ Moreover, tumor suppressors such as PTEN inhibit metabolic adaptation by negatively regulating the PI3K/Akt and MAPK/ERK pathways.^688^

However, owing to the significant differences between intra- and extracellular energy metabolic processes, genetic, environmental, and cellular phenotypic dynamics influence metabolism during tumor development and metastasis.^3^ The metabolic plasticity in this context poses challenges for cancer therapies targeting the Warburg effect, as complete blockade of glucose uptake often proves to be unfeasible. In the quest for novel metabolic targets, approaches such as CRISPR–Cas9-mediated synthetic lethality screens targeting metabolic genes offer a pathway for identifying specific metabolic targets of interest, particularly in vivo. A genome-scale external library of sgRNAs revealed the critical role of the PBAF complex—involved in regulating HIF-1α metabolism markers—in resisting T-cell-mediated killing of B16F10 melanoma cells.^689^

##### Aerobic oxidation

The Warburg theory posits that for many tumor types, cancer cells preferentially generate energy through aerobic glycolysis rather than mitochondrial OXPHOS. However, this notion has been widely contested. Pioneering researchers such as Weinhouse, utilizing isotopic labeling experiments, have demonstrated that the OXPHOS rates of normal and tumor cells are comparable, indicating that mitochondrial functionality in cancer cells is largely preserved.^660,690^ In the presence of oxygen, tumor cells employ both aerobic glycolysis and OXPHOS to support their rapid proliferation.^691^ Glycolysis surpasses OXPHOS to become the dominant energy source only under hypoxic conditions, such as within the tumor core.^660^ This finding underscores that aerobic oxidation remains a vital ATP-generating process in cancer cells. The ability of cancer cells to toggle between glycolysis and oxidation on the basis of their microenvironmental conditions enables them to sustain high proliferation rates.

The process of aerobic oxidation in tumor cells is a complex metabolic pathway that involves the expression of various enzymes and is finely regulated by multiple signaling molecules. During aerobic oxidation, pyruvate produced from glycolysis is converted into acetyl-CoA by the PDH, which then enters the TCA cycle. However, in several cancers, the expression and activity of PDH are disrupted, impairing the normal aerobic oxidation process.^692,693^ Mutations in key TCA cycle enzymes, such as SDH, fumarate hydratase, and IDH, lead to TCA cycle dysfunction and mitochondrial metabolic defects across various human cancers.^694,695^ SDH plays a critical role in tumor suppression. Heterozygous mutations in SDH genes result in complete loss of protein function and are associated with hereditary paragangliomas and pheochromocytomas.^696^ Compared with normal tumors, tumors harboring SDH mutations typically exhibit increased aggressiveness and a significantly faster proliferation rate.^697^ Moreover, mutations in SDH subunits are also linked to other tumor types, including renal cell carcinoma (RCC), neuroblastoma, gastrointestinal stromal tumors, thyroid cancer, and seminomas.^698^ In many cancers, FH expression is downregulated or its function is lost, undermining its role as a tumor suppressor. Reduced FH expression leads to the accumulation of HIF-1α^699^ and high levels of fumarate, a cancer-associated oncometabolite, often resulting in cellular dysfunction in SDH- or FH-deficient cells.^700^ This can also trigger EMT.^701^ IDH is another critical enzyme in the TCA cycle, with mutations observed in multiple solid tumors.^702^ Mutations in IDH impair its ability to catalyze the conversion of isocitrate to α-KG and instead confer neomorphic activity, whereby it reduces α-KG to D-2-hydroxyglutarate (D-2HG) in an NADPH-dependent manner. The excessive accumulation of D-2HG, a tumor-specific metabolite, contributes to the formation of malignant gliomas.^703^

During the progression of malignant tumors, aberrant activation of multiple signaling pathways plays a crucial role in the process of aerobic oxidation. A prominent example is hyperactivation of the Wnt/β-catenin pathway, which promotes tumor angiogenesis by acting on PDK1.^704^ Within this pathway, lipoprotein receptor-related protein 5 (LRP5) serves as a significant coreceptor for signal transduction, facilitating Wnt signaling and further promoting cancer progression by increasing aerobic glycolysis.^705^ Under hypoxic conditions, the expression of HIF-1α is upregulated, promoting glycolysis and adapting to the low-oxygen environment by inhibiting mitochondrial function. Specifically, in Burkitt lymphoma, HIF-1α increases PDK mRNA levels, downregulates OXPHOS,^706^ and induces a reduction in COX4-1 protein levels, thereby decreasing mitochondrial stability. In contrast, the tumor suppressor p53 enhances mitochondrial metabolism and OXPHOS by promoting the assembly of cytochrome oxidase (COX) and glycolytic enzymes.^707^ p53 maintains mitochondrial integrity and positively regulates OXPHOS by upregulating the expression of cytochrome c oxidase 2 (SCO2) and apoptosis-inducing factor (AIF), both of which are essential for the assembly of ETC complexes.^708^

Although cancer cells can generate ATP through aerobic glycolysis, many cancer types, particularly in advanced stages, rely more heavily on mitochondrial OXPHOS to meet their energy demands.^709^ Mitochondria play a critical role in aerobic oxidation, converting various forms of cellular energy into ATP, which is essential for sustaining the metabolic needs of cancer cells.^710^ The overexpression of SALL4 promotes cancer cell metastasis by activating the TGF-β/SMAD signaling pathway and increasing mitochondrial OXPHOS levels.^711^ Cancer cells depend on these metabolic hubs to fulfill their heightened energy requirements and maintain ROS levels, which are crucial for their proliferation, migration, invasion, and metastasis. The overexpression of PTEN in cancer cells increases the protein levels of all respiratory chain complexes and OXPHOS flux while also increasing the levels of mitochondrial transcription factors such as PGC1 and p53.^712^ Activation of the NRF2/HO-1 signaling pathway suppresses ROS expression levels.^713^ Through the TCA cycle, cancer cells can secrete succinate into the extracellular environment, inducing the polarization of tumor-associated macrophages (TAMs), which facilitates EMT.^714^ Specific mitochondrial energy metabolism regulators, such as L-carnitine, BP, PA-2, and DOX, as well as active compounds such as PAB, have shown efficacy in inhibiting tumor cell growth and metastasis.^661^ These molecules function by inhibiting the PI3K/Akt signaling pathway and activating mitochondrial apoptotic pathways. Notably, melatonin increases p53 expression through the PI3K/Akt/mTOR signaling pathway, which plays a significant role in tumor suppression.^715^ Conversely, the oncogene MST1 promotes mitochondrial fission and apoptosis by inhibiting the AMPK-SIRT3 pathway, further impacting cancer cell survival.^661^ Within mitochondria, STAT3 directly interacts with and increases the activity of ND1 and SDH, enhancing OXPHOS flux.^670^ STAT3 protects ND1 and SDH activity from ischemic damage, acting as a ROS scavenger by reducing ROS production from respiratory chain complexes I and II. Moreover, c-Myc upregulates OXPHOS in various cancer cell lines, with high levels of endogenous c-Myc observed under normoxic conditions;^716^ however, its mitochondrial function is severely impaired under hypoxic conditions.

##### Fatty acid metabolism

Cancer cells frequently exhibit significant alterations in lipid metabolism, particularly with increased lipogenesis and fatty acid uptake. Fatty acid synthesis primarily depends on key rate-limiting enzymes: ATP-citrate lyase (ACLY), ACC, and FASN. Studies indicate that reduced expression of ACLY impairs the ability of cells to metabolize glucose into lipids, thereby inhibiting tumor growth.^717^ Elevated expression of FASN is observed in various cancers, including breast and prostate cancers.^718^ Fatty acid uptake is equally crucial in cancer cells and is facilitated by fatty acid transport proteins (FATP) and CD36, two key fatty acid transporters.^719^ In PTEN-deficient prostate cancer, CD36 enhances fatty acid uptake, accelerating cancer progression and suggesting a reliance on exogenous lipid intake.^720^ The increased expression of CD36 is correlated with fatty acid uptake, which supports the energy and structural demands of cancer cells. SREBP1, a primary transcriptional regulator of lipogenesis, is overexpressed in many cancer types. Its activation elevates the expression of key lipogenic genes, such as FASN, ACLY, and ACC.^721–723^ The activation of SREBPs is stimulated by the PI3K/Akt/mTOR signaling pathway, one of the most frequently activated oncogenic pathways in cancer.^724^ ACLY can be directly activated by binding to Akt, and its phosphorylation and acetylation increase its stability, promoting the production of acetyl-CoA, while SIRT2 deacetylates ACLY.^725^ FASN activity is increased through the enhancement of epidermal growth factor signaling *via* the MAPK and PI3K signaling cascades.^726^

When cellular energy demand increases, fatty acids are transported into the mitochondria for β-oxidation, where they are converted into acetyl-CoA and subsequently enter the TCA cycle to generate ATP. CPT1 facilitates this critical and rate-limiting step of FA transport into mitochondria. The role of augmented FAO oxidation in cancer cell proliferation is a subject of debate, with different cancers responding variably to alterations in FAO levels. In certain cancers, enhanced FAO might exert an inhibitory effect by depleting FA availability, thereby restraining tumor growth. Conversely, increased FAO may increase ATP production, supplying cancer cells with surplus energy and potentially supporting their proliferation. Consequently, targeting FAO represents a viable strategy to prevent cancer progression. Pharmacological agents such as etomoxir, a CPT1 inhibitor, and ranolazine, which indirectly inhibits FAO, have been shown to effectively induce apoptosis in cancer cells.^690,727,728^

In cancer cells, the cytosolic NADPH generated from FAO is crucial for mitigating oxidative stress. Under metabolic stress, FAO not only sustains ATP levels but also enhances NADPH production, which is vital for cancer cell survival and proliferation. Studies indicate that inhibiting FAO in glioma cells leads to a marked decrease in NADPH levels and increased accumulation of ROS, ultimately resulting in cell death.^728^ This process is regulated by AMPK, which promotes FAO by inhibiting the phosphorylation of ACC,^729^ thereby maintaining a balance between NADPH consumption in fatty acid synthesis and NADPH production *via* FAO. Additionally, PPAR activation can enhance the FAO pathway.^730^

##### Glutamine metabolism

In cancer, a prevalent metabolic alteration is the upregulation of glutamine metabolism. This amino acid is indispensable for cell proliferation, a fundamental discovery made by Eagle in 1955, demonstrating that proliferative capability is curtailed in its absence. Glutamine is integral to mitochondrial oxidative metabolism, facilitating ROS generation. Its conversion into glutathione (GSH) *via* the enzyme GCL provides antioxidative properties, which are crucial for maintaining cellular redox homeostasis. In mammalian cells, glutamine is a principal energy substrate. Through its catabolism, α-KG is generated, subsequently entering the TCA cycle, thus furnishing tumor cells with critical energy and biosynthetic precursors.^731^ Elevated glutamine levels are frequently observed in cancer patients. During glutaminolysis, glutamine is converted to glutamate by GLS.^732^ Wnt2 signaling pathway activation further augments glutamine metabolism, thereby underpinning the energetic demands of cancer cell proliferation and metastasis.^733^ Glutamate undergoes further degradation through the TCA cycle (*via* conversion to α-KG) or acts as a substrate for glutathione synthesis. Glutathione, a pivotal antioxidant,^734^ is instrumental in mitigating oxidative stress. Increased glutamine metabolism bolsters mitochondrial NADPH production through glutathione synthesis, efficiently quenching ROS.^735,736^

Research indicates that cancer cells exhibit elevated levels of glutamine transporters, particularly ASCT2 (SLC1A5). High ASCT2 expression is associated with increased disease aggressiveness and reduced patient survival.^737^ Under conditions of low glutamine availability, the transcription factor p53 plays a crucial role in prosurvival signaling by stimulating the expression of transporters to increase the uptake of other amino acids, thereby assisting cancer cells in overcoming nutrient scarcity. With p53 influence, aspartate and arginine uptake is increased, with the latter activating mTORC1 to promote tumor growth.^738,739^ Additionally, KRAS can enhance the expression of glutamine metabolism-related genes, including GLS, GLUD, SLC1A5, and transaminases, through the action of Myc.^740^ Myc further activates the expression of genes involved in glutamine uptake and catabolism. Specifically, Myc acts on the promoter regions of glutamine transporters at the transcriptional level, such as SLC1A5 and SLC38A5, facilitating increased glutamine uptake.^741^ Furthermore, PI3K/Akt pathway activation can stimulate NRF2, which in turn upregulates the expression of glutathione synthetase and glutamate-cysteine ligases, which are essential for glutathione production.^742^

##### Electrolytes

Electrolytes such as K^+^ and Mg^2+^ play crucial roles in the metabolic reprogramming of tumor cells, impacting cell proliferation and survival.^743,744^ Magnesium facilitates the killing of target cells by promoting CD8^+^ T cell activation-induced glycolysis through conformational changes in LFA-1. The activity of HK2 is influenced by intracellular K^+^ levels; severe K^+^ depletion disrupts HK2-dependent glycolysis, triggering an energy stress response. In tumors, elevated extracellular K^+^ is a characteristic of the TME, which may lead to a significant reduction in glycolysis intermediates and essential amino acids inside T cells, inhibiting T cell effector function while maintaining cell survival. However, studies suggest that when tumor-infiltrating lymphocytes exhibit higher intracellular K^+^ levels, they can suppress the AKT-mTOR signaling pathway driven by T-cell receptors (TCRs), enhancing their antitumor capabilities. Conversely, excessively high intracellular K^+^ in tumor cells may inhibit the antitumor abilities of tumor-associated macrophages, a process possibly achieved through the readjustment of OXPHOS and glycolysis.^745^

##### Telomere-telomerase system

The link between the telomeres-telomerase system and cancer development is close. Although research on how the telomeres-telomerase system regulates energy metabolism is still insufficient, evidence suggests that telomeres can directly or indirectly regulate the metabolic processes of cancer cells, which, in turn, are influenced by cancer metabolism. The tight connection between telomeres and mitochondrial function is evident in the activation of p53 when telomere function is impaired, leading to the suppression of PGC1α and PGC1β expression.^746^ Reduced expression of PGC1α/β results in mitochondrial biogenesis and functional decline, reducing the expression of genes involved in oxidative defense and thereby affecting the TCA and OXPHOS processes. Additionally, the expression of nuclear respiratory factor 1 and estrogen-related receptor α (NRF-1 and ERRα), which are downstream of PGC, is also inhibited, further exacerbating mitochondrial dysfunction and impeding gluconeogenesis.^747^ Enhancing the expression of TERT or PGC1α or gene knockout of p53 can increase the expression levels of PGC1α/β, G-6-P, and PEPCK, restoring gluconeogenesis.^746^ Elevated TERT expression levels significantly enhance the activity of genes in the glycolysis pathway.^748^ Therefore, telomerase activity may impact cancer cell uptake and utilization of glucose, consequently influencing the glycolysis process. The regulatory role of metabolic enzymes in telomerase function is also crucial. When the expression level of FBP1 increases, FBP1 can interact directly with TERT and induce dephosphorylation of the TERT S227 site. Dephosphorylated TERT cannot translocate to the nucleus, inhibiting telomerase activity in mice and shortening telomere length, thereby suppressing tumor cell proliferation and growth.^749^ These findings provide potential targets for the development of novel anticancer treatment strategies.

##### Interplay between energy metabolism and epigenetic modifications

The intermediate products of energy metabolism impact the process of epigenetic modifications; conversely, epigenetic alterations regulate energy metabolic processes. In cancer research, a recent focus lies on the interplay between energy metabolism and histone modifications, particularly those involving lactylation, palmitoylation, succinylation, and others.^750^ Another crucial aspect is how energy metabolism regulates nucleic acid modifications, including their influence on non-coding RNAs. Genome-wide analyses in cancer have unveiled widespread hypomethylation patterns in DNA, which when present, accompany the activation of transcription, repetitive sequences, transposable elements, and oncogenes, potentially leading to aneuploidy and genomic instability, hallmark features of cancer.^751^ Conversely, tumor suppressor genes undergo significant methylation alterations, which suppress their expression.^752^ Acetyl-CoA, a critical product of energy metabolism pathways, undergoes modulation in cancer cells, impacting gene expression by altering the acetylation status. For instance, KRAS mutations promote acetyl-CoA production, enhancing histone acetylation and boosting glucose uptake *via* an AKT-dependent mechanism.^753^ The role of lactate in chromatin modifications has been historically overlooked, but recent discoveries suggest that lactate promotes histone lactylation, potentially aiding cancer cell invasion.^754^ Despite ongoing research, significant unresolved questions persist. A key challenge is understanding how to coordinate these epigenetic processes to promote tumor development through metabolic regulation. Additionally, the efficacy of epigenetic drugs has been limited primarily to hematological malignancies, proving largely ineffective in solid tumors, possibly due to metabolic and epigenetic heterogeneity within tumors. Despite these challenges, high-throughput technologies exploring the bidirectional crosstalk between energy metabolism and epigenetic regulation, to identify specific epigenetic or metabolic vulnerabilities, remain promising endeavors.

#### Effects of changes in energy metabolism on the immune response within the TME

**Fig. 8:**
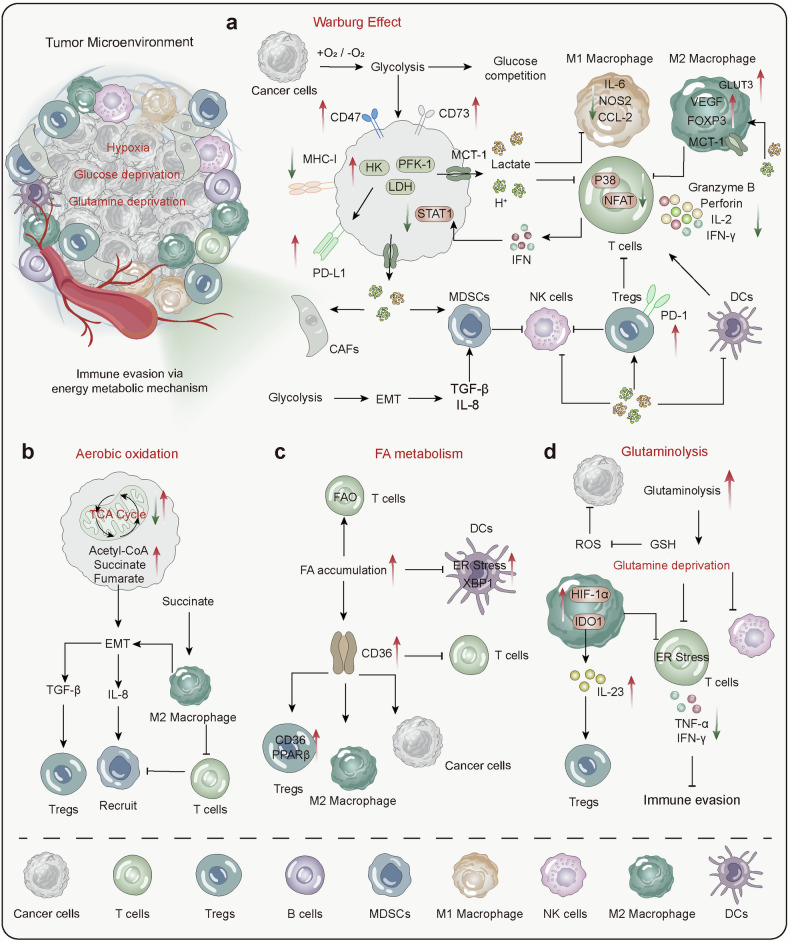
Energy metabolism-driven alterations in the TME. **a** The enhancement of the Warburg effect in cancer cells leads to glucose scarcity in the microenvironment, triggering competition for glucose between immune and cancer cells, which suppresses energy production in immune cells. Increased glycolysis results in the accumulation of lactate and a decrease in pH within the microenvironment. Lactic acid and low pH inhibit the function of M1 macrophages, activated T cells, and NK cells, reducing the secretion of inflammatory cytokines, perforin, and granzymes, thereby diminishing their cytotoxic capabilities. However, lactate promotes the growth of M2 macrophages, MDSCs, Tregs, and CAFs, potentially due to increased expression of glucose transporters and MCT-1, thus facilitating adaptation to the microenvironment. **b** Metabolic shifts in mitochondrial OXPHOS lead to the accumulation of intermediates such as acetyl-CoA, succinate, and fumarate. These intermediates promote epithelial-mesenchymal transition (EMT) in cancer cells and further recruit suppressive cells like MDSCs and Tregs through the release of TGF-β and IL-8. **c** Enhanced FAO promotes the expression of CD36, which facilitates energy production in Tregs, M2 macrophages, and cancer cells but exerts an inhibitory effect on activated T cells and DCs. **d** Increased glutaminolysis leads to glutamine depletion, thereby inhibiting the function of activated T cells and NK cells, reducing the release of pro-inflammatory cytokines such as TNF-α and IFN-γ, and promoting immune evasion. HK hexokinase, PFK phosphofructokinase, LDH lactate dehydrogenase, DC dendritic cells, Treg regulatory T cells, MDSC myeloid-derived suppressor cells, GSH glutathione

The TME is a complex ecosystem consisting of tumor cells, surrounding nontumor cells, and stromal components.^755^ Changes in the energy metabolism of tumor cells significantly impact various immune cells within the microenvironment (Fig. 8). In the TME, tumor cells, effector T cells, and M1 macrophages tend to upregulate glycolysis and glutaminolysis, whereas memory T cells, Tregs, and M2 macrophages primarily rely on FAO.^756^ This metabolic configuration gives Tregs a survival advantage within the TME.^757^ The similarity in energy metabolism demands between tumor and immune cells results in competition for essential energy substrates. However, immune cells fundamentally may not possess the same robust metabolic flexibility as tumor cells. Cytotoxic immune cells are impeded in the TME, leading to an overall skew towards immunosuppressive conditions within the TME, thereby promoting malignancy.

##### Glycolysis

Tumor cells increase glycolysis to meet their proliferative energy needs, facilitating EMT.^758^ This metabolic adaptation also affects stromal cancer-associated fibroblasts (CAFs), influencing their oxidative stress and aerobic glycolysis. Tumor cells then utilize lactate and pyruvate secreted by CAFs to sustain increased proliferation.^759,760^ Mutations, such as those in KRAS, enable cancer cells to withstand nutrient deprivation under hypoxic conditions, allowing them to evade immune surveillance through CD47 regulation.^761^ High glycolytic activity in tumor cells reduces glucose concentrations within the TME. Such glucose and oxygen deprivation can lead to decreased MHC class I antigen presentation in cancer cells, rendering them unresponsive to IFN-mediated cytotoxic effects due to STAT1 dysfunction.^762,763^ On the other hand, upon antigen stimulation, T lymphocytes shift from a quiescent state to a highly anabolic state, increasing their proliferation and differentiation into tumor-killing effector cells. However, glucose scarcity can impair the metabolic fitness and functionality of tumor-infiltrating lymphocytes (TILs) within the TME^764^ and diminish the effector function and viability of NK cells,^765^ thereby constraining the energy metabolism and antitumor capabilities of these immune cells.^766–768^ Effector T cells require substantial amounts of glutamine during activation and proliferation, and deprivation of glutamine in the TME may also impair T-cell functionality.^757,768,769^

In addition to energy competition, key glycolytic enzymes, such as HK, PFK, and LDH, play crucial roles in tumor immune evasion. The increased activity of HK and PFK not only enhances glycolysis in tumor cells but is also associated with the expression of immune checkpoint molecules, such as the upregulation of PD-L1.^770,771^ LDHA is highly expressed in glycolytic tumor cells, and its elevated expression is linked to increased tumor aggressiveness and poor prognosis. Inhibition of LDHA can enhance T-cell-mediated immune surveillance against tumors.^772^

Glycolytic byproducts directly suppress antitumor immunity by influencing inflammation, immune evasion, and tumor angiogenesis. High glycolysis in tumor cells causes lactate accumulation, which is known to suppress all antitumor immune cells. External lactate exposure reduces cytotoxic T-cell proliferation and decreases IL-2, IFN-γ, perforin, and granzyme B levels.^773^ Owing to concentration gradients, excess tumor-derived lactate in the TME inhibits lactate secretion by activated T cells,^774^ leading to endogenous lactate buildup that impairs effector T-cell function.^775^ An acidic pH in the TME can reduce the antigen-presenting capability of dendritic cells, impairing T-cell activation.^756,776^ The accumulation of lactate lowers the intracellular pH and inhibits effector T cells by reducing NFAT nuclear translocation and the activity of p38 and c-JNK/c-JUN.^666^ High lactate levels directly limit NK cell function and indirectly suppress it by increasing myeloid-derived suppressor cell numbers.^777^ Lactate reduces M1 macrophage activity by lowering IL-6, iNOS, and CCL2 expression.^778^ It also shifts M2 macrophages and increases PD-L1 expression to aid in immune evasion.^661^ Lactate induces a tumor-promoting phenotype in M2 macrophages.^779^ Lactate sustains Treg suppressive function by increasing FOXP3 and MCT1 expression.^567^ Increased FOXP3 expression reprograms Treg metabolism by inhibiting c-Myc and glycolysis, increasing OXPHOS, and increasing NAD^+^ oxidation, enabling Tregs to adapt to low-glucose, high-lactate TMEs,^602^ thus suppressing antitumor immunity.^602,780,781^ Another crucial focus lies in F-1,6-BP, which is converted into F-6-P by FBP1, impacting the balance between glycolysis and gluconeogenesis and thereby regulating the equilibrium of these processes. Furthermore, F-1,6-BP serves as a vital precursor in the pentose phosphate pathway and is crucial for maintaining cellular redox balance and synthesizing precursor molecules for nucleic acids. In the realm of tumor biology, F-1,6-BP plays a pivotal role. It can enhance immune responses and bolster the cytotoxic effects on tumor cells. F-1,6-BP activates a positive feedback loop of key metabolic enzymes like PFK1, PI3K/Akt, and PFK2/PFKFB3, enhancing aerobic glycolysis to sustain effector T cell activity while inhibiting oxidative metabolism.^782^ Conversely, studies suggest that F-1,6-BP inhibits AMPK-mediated SENP1-SIRT3 axis activation, leading to reduced T cell memory development.^783^ Moreover, intracellularly accumulated F-1,6-BP can bind with HMGB1, decreasing the affinity of HMGB1 for DNA and DNA adducts and sensitizing cancer cells to DNA replication stress and damage induced by chemotherapeutic agents, thereby promoting cancer cell apoptosis.^784^ F-1,6-BP also induces the expression of apoptotic proteins, facilitating apoptosis in liver cancer cells.^785^ These findings underscore the multifunctionality of F-1,6-BP in tumor metabolism and immune regulation, suggesting novel potential targets for cancer therapy.

##### Lipid metabolism

Lipid metabolism is pivotal in modulating immune responses within the TME. The accumulation of lipids enhances the expression of the scavenger receptor CD36, which has been shown to suppress the effector functions of CD8^+^ T cells, playing a critical role in tumor immune evasion.^786,787^ In dendritic cells (DCs), lipid buildup induces ER stress and activates the transcription factor XBP1, potentially leading to failed presentation of tumor-associated antigens and weakening antitumor immune responses.^788^ Unlike CD8^+^ T cells, Tregs increase fatty acid utilization through CD36, aiding their function and adaptation in the TME and thereby maintaining survival and immunosuppressive capacities.^567,789,790^ CD36 is selectively upregulated in Tregs within tumors, enhancing mitochondrial adaptability *via* PPAR-β signaling. This metabolic reprogramming enables Tregs to thrive in the lactate-rich TME.^789^ Additionally, the availability and utilization of fatty acids by Tregs in the TME are linked to resistance to anti-PD-1 therapies.^791^ In addition to Tregs, TAMs also utilize CD36 to support their tumor-promoting activity by engulfing long-chain fatty acids derived from tumor cells.^792^

##### Amino acid metabolism

Excessive glutamine uptake by cancer cells results in a shortage of glutamine in the TME, tipping the immune balance toward suppression. This lack of glutamine is essential for inducing Treg cell differentiation.^793^ Under glutamine starvation conditions, TAMs activate HIF-1α to produce IL-23, which then increases the number of immunosuppressive Tregs.^794^ These Tregs inhibit cytotoxic T cells through the release of immunosuppressive agents such as IL-10 and TGF-β, aiding in tumor immune evasion.^794^ Studies have demonstrated that CD8^+^ cytotoxic T cells generate significantly less IFN-γ and TNF-α when stimulated in glutamine-deprived environments than when stimulated in glutamine-replete conditions.^795^ This decrease in cytokine production is linked to reduced effector functions of CD8^+^ T cells, highlighting the adverse effects of glutamine deprivation. In ovarian cancer, a lack of glutamine may induce endoplasmic reticulum stress in cytotoxic T cells, depleting their glutamine transporters and impairing their function.^796^ This mechanism may enable cancer cells to evade immune attack, contributing to tumor progression.

Cancer cells and other cells within the TME exhibit extensive intercellular crosstalk in energy metabolism. While the phenomenon of energy transfer between cells has been studied, the intricate connections between these metabolic and nutrient-sensing signaling pathways require further exploration. Additionally, the specific roles played by intermediate products of energy metabolism, such as succinate, fumarate, in carcinogenesis in certain tissues, and their deeper connections with epigenetic modifications, demand further investigation.

Although research on the mechanisms of tumor energy metabolism has been ongoing for several decades and efforts have been made to treat tumors through targeting energy metabolism, the current challenges persist, leading to limited therapeutic outcomes. One aspect of this challenge stems from the inherent characteristics of cancer cells, which exhibit high metabolic flexibility and plasticity. This allows cancer cells to rapidly adapt to unfavorable local environments^797^ and acquire nutrients from the environment and other cells to fuel their growth.^798^ On the other hand, the diverse metabolic products and complex intercellular interactions within the TME lead to significant crosstalk among signaling pathways, making it difficult to predict the efficacy of targeting a specific pathway.^799^ Additionally, due to variations among patients in genetics, environment, diet, and lifestyle, the heterogeneity of cancer microenvironments makes it challenging to identify universal metabolic targets. This shift towards personalized medicine is becoming increasingly emphasized in current research. Despite the numerous challenges, advancements in technology allow for a more comprehensive analysis of energy metabolism changes in the microenvironment through integrating transcriptomics, proteomics, metabolomics, and other omics approaches. Future developments combining molecular probes and biosensors for real-time monitoring of dynamic changes in energy metabolism may help identify appropriate therapeutic windows.

### Integrated interactions among diverse metabolic pathways

The intracellular metabolic network is responsible for maintaining metabolic homeostasis through intricate and precise interactions, thereby ensuring a stable energy supply. In various pathological conditions, such as neurodegenerative and metabolic diseases, the interplay among these metabolic pathways becomes increasingly complex. This complexity involves alterations in molecular regulatory mechanisms, the fine-tuning of enzyme activities, the reorganization of metabolite supply and demand, and substantial restructuring of cellular signaling pathways. These changes are crucial for cellular adaptation to pathological environments, enabling survival and the maintenance of physiological functions. For example, cancer cells exhibit significant metabolic plasticity, switching between glycolysis and OXPHOS *via* complex metabolic networks mediated by HIF-1 and AMPK.^800^ This metabolic flexibility not only supports their proliferation and survival in diverse environments but also contributes to drug resistance, emphasizing the value of understanding and potentially targeting these metabolic pathways in cancer therapy.

To elucidate these sophisticated metabolic regulatory networks and signaling mechanisms, the concept of “metabolic checkpoints” has emerged.^801^ Metabolic checkpoints are molecular mechanisms that sense cellular metabolic states and modulate cellular function in response. This concept advances our comprehension of how cells recalibrate their metabolic pathways to maintain homeostasis across various disease contexts. AMPK serves as a prototypical metabolic checkpoint; in response to decreased energy levels from inhibited glucose and amino acid metabolism, it activates lipolysis and suppresses fatty acid synthesis, thus regulating the balance of glucose and lipid metabolism.^802^ AMPK influences lipid metabolism through several pathways, including the AMPK-PPAR-α-CPT1A axis.^803,804^ Furthermore, PRMT6 has been identified as a novel metabolic checkpoint, facilitating the metabolic shift between FAO and glycolysis. The absence of *Prmt6* enhances FAO-related gene expression *via* reduced H3R2 asymmetric dimethylation on promoters.^805^ Similarly, GCN2 functions as a metabolic checkpoint by phosphorylating eIF2α during amino acid deprivation, inhibiting protein synthesis while promoting the expression of genes involved in amino acid biosynthesis, thereby modulating T-cell metabolism and immune response.^801^

Furthermore, elucidating the metabolic adaptability of bacteria in various environments remains a critical focus for future research. Examining the interactions among diverse metabolic pathways can deepen our understanding of metabolic adaptation mechanisms under pathological conditions and inform the development of innovative therapeutic strategies. This approach holds promise for the design of personalized treatment regimens, offering significant potential in addressing complex metabolic disorders and cancer.

## Advancements for disease detection on the basis of energy metabolism

**Fig. 9:**
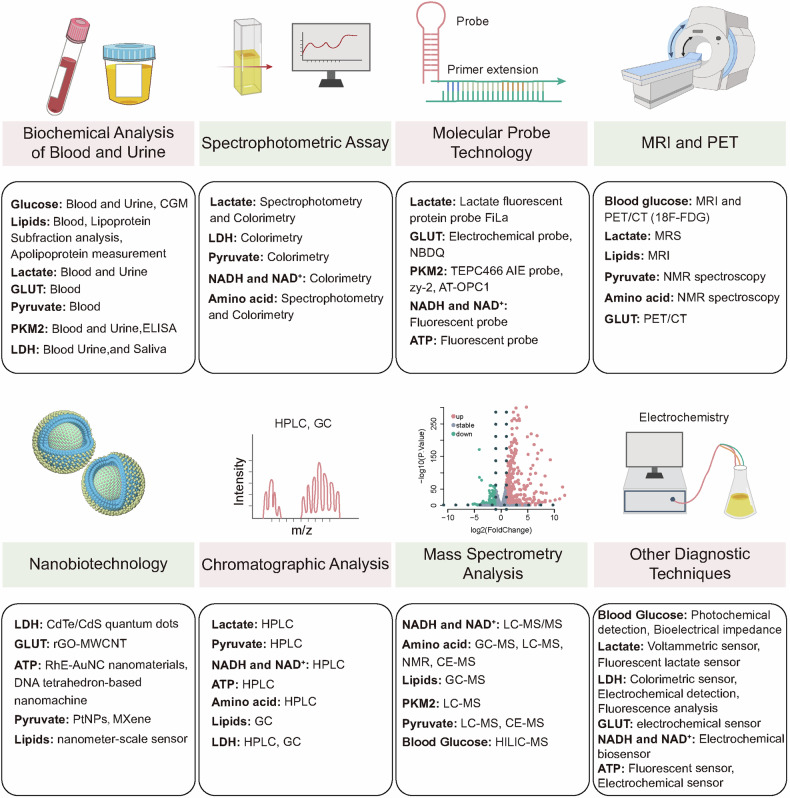
Methods for detecting energy metabolism. MRI magnetic resonance imaging, PET positron emission tomography, HPLC high-performance liquid chromatography, GC gas chromatography, MS mass spectrometry, CE-MS capillary electrophoresis-mass spectrometry

Cellular energy conversion is an intricate, multidimensional process characterized by the dynamic interplay of ions, metabolites, and biochemical products. Disruptions in energy metabolism, or imbalances and dysfunctions in these metabolites, are frequently associated with the pathogenesis of diseases such as diabetes, malignancies, neurodegenerative disorders, and cardiovascular conditions.^806,807^ Alterations in key metabolic intermediates, particularly those involved in glycolysis and mitochondrial oxidative pathways, such as glucose, lactate, NADH, ROS, glutamate, and ATP, hold potential as biomarkers for disease diagnosis.^806^ A range of advanced technologies are employed to investigate energy metabolism for diagnostic purposes, including blood and urine biochemical assays, spectrometry, bioprobes, magnetic resonance imaging (MRI) and positron emission tomography (PET) imaging, nanotechnology, metabolomics, and techniques such as electrochemistry and sensing (Fig. 9). These methods play a vital role in identifying metabolic abnormalities and elucidating disease mechanisms.

However, each of these techniques has its own advantages and limitations (Table 1). NMR and MS are the most commonly used techniques in metabolomics research. NMR is non-destructive for sample or disease detection, with better reproducibility of results. However, it requires higher concentrations of metabolites, affecting sensitivity.^808^ On the other hand, mass spectrometry has the advantage of detecting very low concentrations of metabolites but often lacks reproducibility due to the influence of various ions or proteins in the samples and cannot be directly used for in vivo detection.^808^ Mass spectrometry focuses on metabolite levels, and stable isotope labeling can non-destructively provide dynamic information about metabolites and can be used for in vivo monitoring, but safety issues regarding some radioactive isotopes must be considered.^809^ Compared with mass spectrometry techniques, fluorescence analysis offers the advantages of convenience, sensitivity, and selectivity. Despite these benefits, the use of basic fluorescence analysis remains limited. This is because fluorescence probes are employed mainly for individual metabolic reactions and metabolites, limiting the ability to conduct flux analysis across entire metabolic pathways.^810^ On the other hand, research on biologically nanostructured materials such as biosensors is currently a hot topic, as they can continuously and non-destructively monitor specific changes in various circulating metabolites, with improved sensitivity and accuracy, enabling personalized prevention, diagnosis, and treatment.^811^ Spatial metabolomics based on mass spectrometry imaging provides subcellular resolution (2 μm), offering precise spatial distribution information of metabolites in tissues. However, it can only be used for ex vivo tissue detection, and challenges in slicing adipose and bone tissues may reduce accuracy, while the complexity of the data adds to the difficulty of integration and analysis. Therefore, integrating these techniques in current research will be beneficial for improving the accuracy and sensitivity of studies. The following section introduces the applications of these techniques in detecting specific energy metabolic pathways.

**Table 1:** Principles, advantages, and disadvantages of different detection techniques

	MS	HRMS	NMR	SERS	Spatial Metabolomics	Biosensors	Fluorescent probe	Isotope Tracing	Optical/Electrochemical Techniques
Sensitivity	High Sensitivity	Higher Sensitivity	Medium Sensitivity	High Sensitivity	High Sensitivity	High Sensitivity	High Sensitivity	High Sensitivity	Exist Differences
Detection limit	Nanomole	Femtomole	Micromole	Nanomole	Nanomole	Nanomole	Nanomole	Picomole	Nanomole
Selectivity	Selective or Non-Selective Analysis	Selective or Non-Selective Analysis	Commonly used for Non-Selective Analysis	Selective or Non-Selective Analysis	Selective or Non-Selective Analysis	Selective Analysis	Selective Analysis	Selective Analysis	Selective Analysis
Destruction	Destructive	Destructive	Non-Destructive	Non-Destructive	Non-Destructive	Non-Destructive	Non-Destructive	Non-Destructive	Non-Destructive
Reproducibility	Moderate	High	High	Moderate	High	High	High	High	High
Dynamic monitoring	No	No	No	No	Yes	Yes	Yes	Yes	Yes
Spatial information	No	No	Yes	Yes	Yes	No	Yes	Yes	No
Sample preparation	Required	Required	Optional	Optional	demanding	Not Required	Not Required	demanding	Not Required
Time consumption	Lengthy	Lengthy	Rapid	Rapid	Lengthy	Rapid	Rapid	Rapid	Rapid
In vivo studies	No	No	No	No	No	Yes	Yes	Yes	Yes

*MS* Mass spectrometry, *HRMS* High-resolution mass spectrometry, *NMR* Nuclear magnetic resonance, *SERS* Surface-enhanced raman spectroscopy

### Glucose and lipid detection

Glucose is a fundamental energy source for cells, and its regulation is intricately linked to numerous pathological states, most notably diabetes. Conventional methods for glucose monitoring include capillary blood glucose testing, venous blood glucose analysis, and urine glucose testing. Each method presents limitations: venous blood testing demands frequent sampling and lacks immediacy, whereas urine tests are heavily influenced by renal function, rendering them unsuitable for severe cases. In light of these limitations, innovative glucose monitoring technologies have been developed. Continuous glucose monitoring (CGM) systems have emerged as formidable tools that deliver real-time, continuous glucose data, which enables proactive management of diabetes and the prevention of complications by detecting glucose fluctuations promptly.^812^ Recent technological advances have introduced noninvasive glucose monitoring techniques such as photochemical, microwave, and bioimpedance methods. The photochemical approach measures glucose changes on the surface of the skin, whereas bioimpedance assesses the electrical resistivity of biological tissues. Despite their noninvasive nature and potential for real-time accuracy, factors such as skin thickness and moisture can affect precision. As technology evolves, these novel glucose detection methods promise more comprehensive, timely glucose data, offering increased support for managing diabetes.

Abnormal lipid metabolism, including dyslipidemia, is a significant risk factor for cardiovascular diseases. Traditional lipid assessments measure total cholesterol, LDL, HDL, and triglycerides and are extensively employed to evaluate cardiovascular risk and inform treatment strategies.^813^ Advances in the analysis of lipoprotein subfraction and apolipoprotein levels have provided nuanced insights into lipoprotein particle size and concentration, enabling the identification of individuals at increased risk of atherosclerosis.^814^ These advancements pave the way for individualized approaches to lipid management, aiding in the prevention of cardiovascular complications.

### Lactate detection

Lactate is essential for numerous cellular functions and plays a significant role in regulating energy metabolism and signaling. Its buildup in tissue microenvironments is characteristic of many inflammatory and cancerous conditions.^778^ The Warburg effect, often linked to cancer, is also observed in noncancer diseases such as pulmonary hypertension, fibrosis, heart failure, atherosclerosis, and polycystic kidney disease.^815,816^ Under stress conditions such as trauma or infection, the body produces lactate through aerobic glycolysis. Thus, lactate serves as a biomarker for various conditions: urinary lactate indicates diabetic nephropathy,^817^ whereas cerebrospinal fluid lactate provides prognostic insights into cryptococcal meningitis.^818^

In diagnostics, lactate levels are typically measured *via* enzyme-based spectrophotometric and colorimetric methods. In clinical settings, especially intensive care, lactate is quantified *via* LDH-catalyzed reactions, producing NADH, which is measurable by spectrophotometry at 340 nm and correlates with plasma lactate levels.^819^ Other techniques include voltammetric sensors using ferrocene boronic acid^820^ and NADH-based fluorescence methods.^821^ Moreover, high-performance liquid chromatography (HPLC) with fluorescence or mass spectrometry detection is used to analyze lactate in biological samples such as saliva, plasma, and urine.^822,823^

Traditional detection methods, such as blood, breath, and urine tests, lack the capability for in vivo and live-cell spatiotemporal monitoring, leaving several aspects of lactate metabolism unexamined. Recent innovations include FiLa probes developed by Zhao and collaborators. These advanced fluorescent probes allow high-resolution, real-time lactate monitoring at the subcellular level, surpassing traditional methods such as Seahorse analysis.^754,824,825^ FiLa technology offers in situ, quantitative tracking of lactate dynamics, an enhanced understanding of lactate distribution, regulatory networks, drug screening, and clinical diagnostics. The team also pioneered dynamic imaging with cell encapsulation systems and portable, high-throughput techniques for rapid lactate detection in small fluid samples, providing a versatile solution.^826^ Additionally, magnetic resonance spectroscopy (MRS) offers a valuable alternative for assessing lactate metabolism, capturing data unattainable by blood tests alone, as shown in studies on the impact of dichloroacetate in neurological and cancer situations.^827^

### LDH detection

LDH is key in converting lactate to pyruvate.^828,829^ Subtypes such as LDHA convert pyruvate to lactate, whereas LDHB prefers lactate to pyruvate.^828,830,831^ Normal LDH levels are under 200 U/L; higher levels can indicate conditions such as malaria,^832–834^ myocardial infarction, and more.^835^ Persistently high levels postsurgery may suggest residual tumors, whereas decreases indicate successful removal.^836^ LDH serves as a biomarker for cancers, including colorectal^837^ and lung cancer,^838^ and it aids in pleural effusion and chemotherapy guidance.^839^ This underscores the demand for reliable, simple LDH detection methods.

Colorimetric assays are popular because of their affordability and simplicity, allowing easy concentration measurement.^840–842^ Researchers have crafted a high-stability colorimetric biosensor for LDH, detecting levels as low as 13 U/L in under 5 min, using minimal reagents and imaging technology for quantification.^843^ A paper-based sensor employing a magnetic immunoassay reached a 0.39 U/L detection limit within 20 min.^844^ Moreover, a rapid, cost-effective paper-based prototype offers LDH level assessments in less than 4 min, which is ideal for resource-constrained environments, utilizing minimal blood volume and smartphone technology for analysis.^843,845^

Despite the simplicity of colorimetric methods, their limited sensitivity can hinder broader application.^841,846^ To address this, a straightforward spectrophotometric assay for salivary LDH detection has been developed, employing NADH oxidation measured at 340 nm. Additionally, a microfluidic microplate-based immunoassay offers rapid detection with reduced antibody use compared with traditional ELISA, reaching limits as low as 6.25 × 10^−3^ U/L. The challenge of external influences on UV spectrophotometry, such as sample coloration, is mitigated by electrochemical methods, which rely on electrical signal changes and offer advantages in cost and speed *via* techniques such as cyclic voltammetry, differential pulse voltammetry, and square wave voltammetry.^847,848^

Widely used in detecting LDH, immunoassays have increased the use of gold nanoparticle-based sensors, which are crucial for detecting elevated LDH in malaria patients.^849^ These sensors offer heightened sensitivity and reproducibility over commercial kits and are suitable for integration with mobile technology.^850^ Fluorescent analysis, which uses approaches such as CdTe/CdS quantum dots and SiQDs, provides high sensitivity and a broad linear range.^851,852^

While current LDH detection methods meet basic needs, innovations are essential for better early screening, with a focus on sensitivity, cost reduction, and improved point-of-care solutions.

### GLUT detection

GLUTs are specialized proteins embedded in cell membranes that facilitate glucose movement through diffusion or secondary active transport.^853,854^ Humans have 14 identified GLUTs categorized into three classes (I, II, III) on the basis of their sequence similarity.^855^ Research has linked GLUTs with various diseases, suggesting their potential as biomarkers. For example, lower serum levels of GLUT1 and GLUT4 are found in patients with both hypothyroidism and heart failure.^856^ Neurodegenerative diseases involve decreased GLUT1 and GLUT3, impairing glucose metabolism.^857^ Furthermore, low GLUT1 expression in the blood is a predictor of severe COVID-19 outcomes.^858^ The number of cases of gestational diabetes is increasing, with studies reporting higher GLUT1 and GLUT3 levels in the placenta than in healthy individuals.^859^ In T2DM, increased intestinal GLUT2 expression is observed.^860^ Early AD is characterized by reduced neuronal GLUT1 and GLUT3, leading to lower D-glucose levels.^281^ GLUTs also play roles in cancer, where high GLUT1 is linked to poor prognosis in lung, bladder, and oral cancers.^861–863^ Elevated GLUT3 is related to unfavorable outcomes in cancers of the lung, larynx, and oral cavity;^861,863,864^ thus, GLUTs could serve as diagnostic biomarkers.

Advancements in detecting GLUTs show promise. A study used an rGO-MWCNT composite with a TBO-graphene-gold nanoparticle-GLUT1 antibody as an electrochemical probe, creating a detection platform for live cells. This sensor provides a linear range of 10^^5^ cells/mL and shows stability and selectivity in detecting GLUT1 across tumor cell types, matching conventional methods such as flow cytometry and Western blotting. It can assess tumor malignancy and differentiate glucose uptake paths, offering cost-effective healthcare solutions.^865^ Another innovation, is that the NBDQ probe acts as a nonantibody GLUT1 inhibitor and is 30 times more sensitive than traditional tracers in cancer imaging because of the Warburg effect. It shows superior tumor selectivity and biocompatibility in vivo, especially in triple-negative breast cancer models.^866^

### Pyruvate detection

Pyruvate, a key glycolysis product, is essential in cellular energy processes. It is generated in the cytoplasm and fuels the Krebs cycle and OXPHOS in mitochondria.^867^ Under aerobic conditions, pyruvate is oxidized to produce ATP. Under anaerobic conditions, it is converted to lactate. Interestingly, some cells opt for pyruvate reduction even with oxygen, a process known as the Warburg effect, which maintains energy equilibrium.^868^ Irregular pyruvate levels are associated with conditions such as diabetes, liver cirrhosis, cardiovascular issues, and neurological disorders.^819,869^ Its measurement is gaining importance in cancer diagnostics.^870,871^

Despite its diagnostic potential, pyruvate detection in emergency settings is limited by selectivity issues, as its levels are lower than those of lactate. Proton nuclear magnetic resonance (NMR) facilitates quantitative and qualitative pyruvate analysis.^872,873^ HPLC combined with fluorescence or mass spectrometry is used to measure pyruvate in body fluids such as saliva and plasma.^822,823^

Traditional methods, such as ELISA, face challenges such as time consumption and specificity issues. In contrast, biosensors offer rapid, sensitive, and simple detection. A recent biosensor employing platinum nanoparticles on 2D MXenes was developed to detect pyruvate in serum efficiently, with a wide detection range and a low detection limit of 0.7 μM, which was validated in human serum analysis.^874^ The potential of MXenes as biosensing materials is promising for diverse sensor applications. Additionally, sensors that use Lewis acid/base interactions with diphenylboronic esters for pyruvate detection are being explored for bioimaging purposes.^875^

### PKM2 detection

PKM2, a key glycolytic enzyme, is a prominent PK isoform in mammalian cells because of its vital role in metabolic reprogramming in cancer and active immune cells.^876^ It serves as a promising marker and therapeutic target in various conditions. Notably, elevated PKM2 levels in the urine of diabetic patients, which are absent in healthy individuals, suggest its potential as a biomarker for early diabetic nephropathy.^877^ PKM2 is also an early marker for acute kidney injury.^878^ Its utility as a rapid, noninvasive biomarker for the early detection of structural colon diseases could reduce unnecessary endoscopies.^879^ PKM2 overexpression in cancers aids in tumor growth and spread,^880^ reinforcing its role as both a diagnostic marker and a therapeutic target, with significant diagnostic implications.

Recent advancements in PKM2 detection include the TEPP-46-based AIE probe TEPC466, which shows high selectivity and sensitivity for PKM2 *via* the AIE effect and is particularly useful in imaging colorectal cancer cells for diagnosis and treatment.^881^ Additionally, the fluorescent probe zy-2 was developed for specific imaging of PKM2 and is able to track it in real time on the basis of concentration and time in PKM2-positive cells, making it ideal for cancer detection.^882^ Furthermore, the AT-OPC1 probe is designed to label PKM2 at the Lys305 site and uses electrophilic reactivity for precise protein detection through gel-based proteome imaging and real-time cell imaging, offering significant potential in cancer diagnostics.^883^

### NADH and NAD^+^ detection

NADH and NAD^+^ play pivotal roles in metabolic processes such as OXPHOS, the TCA cycle, and glycolysis.^884^ They are integral to cellular oxidation, signal transduction, and safeguarding DNA repair mechanisms.^885^ Imbalances in the NAD^+^/NADH ratio are closely associated with conditions such as PD and are associated with elevated NADH concentrations in breast cancer cells relative to those in normal cells. Thus, sensitive and selective real-time monitoring of NADH is essential for diagnosing diverse pathological states and monitoring therapeutic efficacy.

In recent advancements, electrochemical biosensors have emerged as promising alternatives to conventional optical assays such as absorbance or fluorescence-based measurements. Researchers have developed sophisticated sensing technologies, including the synthesis of silver nanoparticles with various morphologies, such as nanorods, nanoprisms, and nanospheres. These nanoparticles are utilized in NADH sensors that are both simple and highly sensitive.

Further innovations involving genetically engineered biosensors such as SoNar, which are designed to monitor NAD^+^/NADH ratios in live cells, specifically targeting mitochondrial SoNar or the cytosolic SoNar, have been reported. These biosensors provide fluorescence signals that linearly correlate with in situ physiological NAD^+^/NADH ratios. The differing responses of the cytosolic and mitochondrial NAD^+^/NADH ratios to acute metabolic perturbations highlight distinct NAD pools. These ratios are modulated by NAD^+^ precursor availability and are significantly altered under pathophysiological conditions. The deployment of compartment-targeted biosensors alongside real-time imaging offers profound insights into subcellular NAD^+^/NADH redox dynamics, enhancing future research into the mechanistic roles of NAD^+^/NADH redox in cellular physiology and disease progression.^886^

### ATP detection

ATP is the principal energy currency in human cells and is critical for various physiological and pathological processes.^887^ It supports energy balance, metabolic regulation, and cellular communication, playing a key role in conditions such as neurodegenerative and cardiovascular diseases, immune disorders, diabetes, cancer, and obesity.^888–895^ Precise ATP measurement is essential for understanding disease mechanisms and enhancing diagnostic capabilities.

Despite its importance, efficient and equipment-free ATP monitoring is challenging. Researchers have developed innovative hydrogel microneedles embedded with ATP-specific dual-emission gold nanoclusters. These microneedles allow quick ATP sampling and detection, providing visual identification with high sensitivity, marking a significant advancement in ATP-sensing technologies.^896^ Additionally, a biosensor with a two-dimensional DNA structure and multiple ATP aptamers enables rapid and sensitive ATP detection. This system capitalizes on aptamer-induced conformational changes, cutting the detection time to 30 min and achieving a detection limit as low as 0.3 pM.^897^

Multiplexed detection systems have significant implications for disease diagnostics. A comprehensive sensor platform using enzyme-coupled reactions simultaneously detects ATP and lactate. It features dual electrodes on a microcontroller-driven potentiostat chip, incorporating enzymes such as adenylate kinase and PK to generate hydrogen peroxide, resulting in resistance to interference from blood components such as ascorbate and urate.^898^

Emerging ATP detection technologies, including ATP-targeted fluorescent probes^899^ and nanomaterial-based signal amplification methods,^900^ continue to progress. These advancements offer unprecedented sensitivity and speed, enhancing biomedical applications and disease diagnostics.^901^

### Amino acid metabolism detection

Detection methods for amino acid metabolism are crucial for diagnosing and monitoring diseases such as diabetes, neurological disorders, cardiovascular conditions, and cancer, as disruptions in this metabolism are common across these ailments. In diabetes, abnormal amino acid metabolism is linked to insulin resistance, with increased blood amino acid levels. Neurological disorders often involve altered amino acid metabolism, which affects neurotransmitter processes. In cancer, amino acid metabolism dysregulation is tied to tumor growth and metabolic changes. Therapeutically, targeting amino acids, such as asparaginase, in leukemia treatment has demonstrated clinical potential through the disruption of specific amino acid pathways.

Amino acid metabolism detection employs two main methodologies: biochemical analyses and advanced metabolomics. Techniques such as HPLC, gas chromatography‒mass spectrometry (GC‒MS), and liquid chromatography‒tandem mass spectrometry (LC‒MS/MS) are vital for measuring amino acids in samples such as blood and urine.^902–904^ HPLC separates amino acids and applies specific detection methods.^905^ GC‒MS combines chromatography and mass spectrometry and uses spectral profiles to identify and quantify amino acids.^904^ LC‒MS/MS combines liquid chromatography with mass spectrometry, which is sensitive, making it effective for analyzing amino acids in biological matrices.^906^ Nuclear magnetic resonance spectroscopy can be used to analyze several metabolites, including amino acids, simultaneously.^907,908^

Metabolomics leverages high-throughput sequencing and bioinformatics to offer comprehensive insights into amino acid metabolic pathways.^909^ These advanced technologies allow the study of global alterations in amino acid metabolism, the identification of metabolic products, and the understanding of their roles in disease progression, providing critical insights into the biochemical foundations of health and disease.

### Platelet mitochondrial detection and disease diagnosis on the basis of energy metabolism

Mitochondria are crucial for cellular energy metabolism, and their dysfunction is intricately associated with a variety of diseases.^910^ Mitochondrial impairment serves as an important biomarker for a range of conditions, including neurological, cardiovascular, infectious, cancerous, and metabolic disorders.^911–916^ Innovative blood-based bioenergetic tests offer a promising noninvasive alternative to traditional tissue biopsies, providing new avenues for assessing mitochondrial function.^910,917^ Platelets have been validated as indicators of both systemic and tissue-specific bioenergetic shifts in various diseases owing to their abundance, accessibility, and active metabolic role.^918–920^

In neurodegenerative disorders such as AD and PD, mitochondrial dysfunction in platelets may serve as a biomarker for early diagnosis and tracking of therapeutic efficacy, reflecting bioenergetic changes directly linked to these diseases.^921–925^ In cardiovascular diseases, examining platelet mitochondrial function can provide insights into conditions such as peripheral artery disease, where exercise interventions have been shown to enhance mitochondrial performance, thereby improving patients’ physical capabilities and quality of life.^926,927^ Moreover, platelet bioenergetics can serve as a prognostic tool in acute infections such as sepsis and chronic infections such as HIV, indicating mitochondrial dysfunction and informing treatment strategies.^928–932^ In metabolic disorders, such as T2DM and cardiovascular disease, changes in platelet metabolism can mirror the bioenergetic states critical for disease tracking and predicting treatment responses.^933,934^

### Noninvasive metabolic imaging and disease diagnosis

Noninvasive imaging technologies have revolutionized the assessment of metabolic processes within the body. Techniques such as PET and MRI are crucial for visualizing tissue metabolism. PET, particularly with the use of the glucose analog 18F-fluorodeoxyglucose (FDG), is an effective tool for detecting metabolically active tumors, assessing treatment responses, and monitoring disease progression.^935^ The advent of hybrid PET/MRI systems has advanced metabolic imaging by combining functional and structural data, greatly improving the diagnosis and management of cancer and metabolic disorders.

New metabolic pathways and molecules are increasingly recognized as potential biomarkers. Enzymes and metabolites from the one-carbon metabolism pathway, such as 10-formyltetrahydrofolate dehydrogenase (FDH) and hydroxyprostaglandin dehydrogenase, are often dysregulated in cancers, contributing to tumor development. Additionally, genes related to OXPHOS, such as UQCRQ, NDUFB7, and UQCRC2, are frequently downregulated in gastric cancer, suggesting their potential as prognostic markers.^936^ Investigating these pathways offers new biomarkers that can enhance early disease detection. Although the discovery and validation of these biomarkers demand extensive collaborative research, their potential impact on diagnostics and therapy is significant.

Advancements in metabolism-focused disease diagnosis have immense clinical importance. Continuous glucose and lipid metabolism monitoring has greatly improved diabetes and cardiovascular disease management, enabling early interventions and reducing complications. Noninvasive imaging techniques have transformed cancer care by allowing precise detection of active tumors and assessing treatment efficacy. Additionally, metabolomics and biomarker discovery offer new insights into disease mechanisms, supporting the development of personalized treatment strategies. Genetic testing has revolutionized the diagnosis of hereditary metabolic disorders, significantly advancing precision medicine. The integration of these diagnostic advancements with emerging technologies such as artificial intelligence and big data analytics holds great promise. The wealth of data from metabolic monitoring and omics methodologies can be used to develop predictive models and decision support systems, fostering early detection and customized treatment plans. Moreover, the discovery of novel metabolic pathways and therapeutic targets could result in innovative treatments specifically designed for metabolic abnormalities, heralding a new era in precision healthcare.

In the field of energy metabolism detection technologies, current challenges primarily include the sensitivity and specificity of the techniques. Owing to significant differences in metabolite concentrations, the detection of low-concentration metabolites demands increased sensitivity from these technologies. Additionally, sample complexity poses a challenge, particularly when dealing with tissue samples or in vivo detection, as the crossover of different cell types and metabolic pathways may affect the accuracy of data interpretation. Moreover, the complexity of data integration and analysis serves as a bottleneck; further research is needed to effectively integrate and accurately interpret data from various technologies.

## Advancements for targeting energy metabolism for disease intervention

Energy metabolism is essential for maintaining cell viability, converting nutrients into energy, and ensuring their efficient use within the cell. Balancing this process is vital for cellular health. Disruption of this gene can lead to diseases such as neurodegenerative disorders, cardiovascular issues, and cancer. Therefore, managing and targeting energy metabolism presents significant therapeutic opportunities. Researchers are developing strategies to address metabolic imbalances. One method involves targeting key enzymes or pathways with drugs or gene therapy to restore equilibrium. Another strategy is to adjust patients’ diets and lifestyles, such as adopting low-sugar diets or increasing physical activity, to positively affect metabolic health. Furthermore, innovative therapies, such as pairing metabolism-targeting drugs with standard chemotherapy for improved cancer outcomes, are being explored to improve existing treatments or disease responses.

This section reviews therapeutic targets in energy metabolism disorders, existing drugs, and current clinical trials. These investigations provide fresh insights into disease treatment and steer future medical progress. By understanding and precisely intervening in energy metabolism, we strive to develop more effective and personalized patient treatments.

### Targeting energy metabolism for cancer therapy

**Fig. 10:**
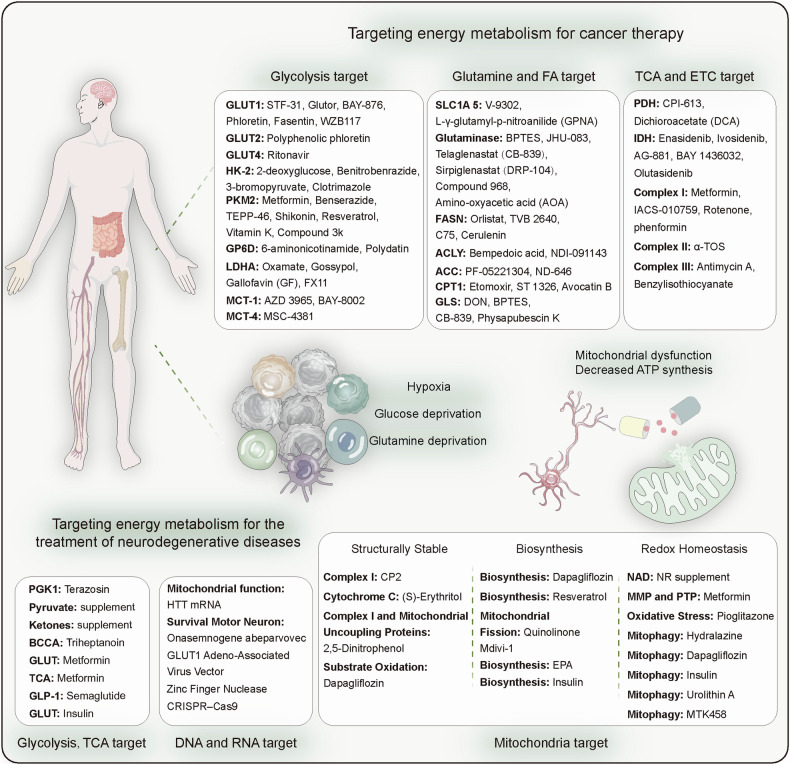
Targeting energy metabolism for cancer and neurodegenerative disease therapy. In cancer, owing to the significant increase in glycolysis, therapeutic drugs are often used to target the glycolytic process. In neurodegenerative diseases, because of mitochondrial dysfunction, therapeutic drugs are often used to target the mitochondrial metabolic process

Metabolic reprogramming, recognized as a fundamental mechanism in cancer progression, provides novel perspectives and strategic avenues for tumor therapy. In-depth exploration of the metabolic characteristics of tumor cells, coupled with the development of targeted therapies that exploit these specific traits, represents a critical and promising trajectory for future cancer research endeavors (Fig. 10 and Table 2).

**Table 2:** Summary table of selective potential drugs/compounds targeting cancer metabolism pathways

Category	Target	Drug/compound	Mode of action	Stage of Drug Development	References
Glycolysis	GLUT1	STF-31	Blocks glucose uptake	Preclinical	^1211^
		Glutor		Preclinical	^1212^
		BAY-876		Preclinical	^1213,1214^
		Phloretin		Preclinical	^945,1215^
		Fasentin		Preclinical	^1211^
		WZB117		Preclinical	^940,1216^
	GLUT2	Polyphenolic phloretin		Preclinical	^942,1217^
	GLUT4	Ritonavir		NCT01009437, NCT03147378	^948^
	HK2	2-deoxyglucose	Blocks glycolytic flux	NCT 00633087	^955^
		Benitrobenrazide		Preclinical	^1218,1219^
		3-bromopyruvate		Preclinical	^950,957,1220,1221^
		Clotrimazole		Preclinical	^950^
	PKM2	Metformin	Prevents ATP production in hypoxia cancer cells	Preclinical	^1222^
		Benserazide		Preclinical	^964^
		Shikonin		NCT01968928	^966^
		Resveratrol		NCT00256334, NCT02261844	^968,1223^
		Vitamin K		Preclinical	^969^
		Compound 3k		Preclinical	^973^
		TEPP-46	Promotes oxidative cellular damage in oxygenated cancer cells	Preclinical	^1224^
	LDHA	Oxamate	Pyruvate competitive	Preclinical	^975,977,978^
		Gallofavin	Binding of free enzyme	Preclinical	^985^
		Gossypol	NADH competitive	NCT00540722, NCT01977209	^1225–1227^
		FX11		Preclinical	^982,984^
	MCT1	AZD3965	Inhibition of glycolysis	NCT01791595	^1228,1229^
		BAY-8002		Preclinical	^991^
	MCT4	MSC-4381		Preclinical	^992^
	Glucose-6-phosphate dehydrogenase (G6PDH)	6-aminonicotinamide	Inhibition of glycolysis	Preclinical	^1230^
Glutamine Metabolism	SLC1A5	V-9302	Inhibition of glutamine transport	Preclinical	^1231^
		L-γ-glutamyl-p-nitroanilide (GPNA)	Inhibits glutamine uptake and inhibits mTOR activation	Preclinical	^996,997,1002^
	Glutaminase	BPTES	Allosteric inhibitor mTOR signaling downregulation and inhibition of TCA anaplerosis	Preclinical	^1010–1013^
		Telaglenastat (CB-839)		NCT03163667, NCT03428217, NCT03831932, NCT03875313, NCT03965845, NCT03057600, NCT03047993, NCT02861300, NCT03528642, NCT03798678, NCT05521997	^1017,1018^
		Compound 968		Preclinical	^1019^
		JHU-083	Inhibition of glutamine metabolism	Preclinical	^1232,1233^
		Sirpiglenastat (DRP-104)		NCT 04471415, NCT 06027086	^68^
	Glutamine aminotransferase	Amino-oxyacetic acid (AOA)	Inhibits transamination	Preclinical	^1021^
	FASN	Orlistat	Inhibition of de novo lipid synthesis	Preclinical	^1022,1028,1234^
Fatty Acid Metabolism		TVB2640		NCT02980029, NCT03808558, NCT03179904, NCT05743621, NCT03032484	^1024^
		C75		Preclinical	^1235^
		Cerulenin		Preclinical	^1236^
	ACLY	Bempedoic acid	Inhibition of de novo lipid synthesis	Preclinical	^1035^
		NDI-091143		Preclinical	^1036^
	Acetyl CoA carboxylase	PF-05221304	Inhibition of de novo lipid synthesis	NCT 03248882	^1237^
TCA cycle OXPHOS		ND-646		Preclinical	^1018^
	CPT1	Etomoxir	Inhibition of fatty acid oxidation	Preclinical	^727,1038^
		ST1326		Preclinical	^1043^
		Avocatin B		Preclinical	^1047^
	Pvruvate dehydrogenase	CPI-613	Inhibition of mitochondrial metabolism	NCT03699319, NCT01832857, NCT01931787	^1238,1239^
	IDH	Enasidenib	DNA and histone hypermethylation	NCT01915498	^1050,1053^
		Ivosidenib		NCT02074839, NCT03173248, NCT04250051, NCT03471260, NCT02989857	^1050,1240,1241^
		AG-881		NCT02492737	^1242^
		BAY 1436032		NCT03127735, NCT02746081	^1243,1244^
		Olutasidenib		NCT02719574, NCT03684811	^1052^
	Mitochondrial respiration complex I	Metformin	Inhibits mitochondrial complex	Preclinical	^1245^
		IACS-010759		Preclinical	^1055^
		Rotenone		Preclinical	^1056,1057^
	Mitochondrial respiration complex II	α-TOS	Inhibits mitochondrial complex	Preclinical	^1058^
	Mitochondrial respiration complex III	Antimycin A	Inhibits mitochondrial complex	Preclinical	^1246^
		Benzylisothiocyanate		Preclinical	^1059^

#### GLUT

GLUTs play crucial roles in the metabolism of cancer cells, with their overexpression linked to increased glucose uptake, which is characteristic of their glycolytic phenotype. This association makes GLUTs attractive targets for cancer therapies.^937,938^ However, their ubiquitous presence in all cell types complicates their selective inhibition.

GLUT1 inhibitors, including STF-31, GLUTOR, and BAY-876,^939^ have shown promising anticancer effects by reducing glucose uptake in cancer cells, subsequently suppressing tumor growth. For example, the natural compound WZB117 blocks GLUT1 and inhibits lung and breast cancer cell proliferation while increasing the efficacy of chemotherapies such as cisplatin and paclitaxel.^940^ Additionally, BAY-876 has been used to increase the efficacy of PD-1 checkpoint inhibitors in models of pancreatic and lung cancer.^941^ However, GLUT3 overexpression might counter some effects of GLUT1 inhibition, indicating that dual targeting of GLUT1 and GLUT3 could be more effective. Phloretin, an apple-derived compound, acts as a GLUT2 antagonist in triple-negative breast cancer. Studies suggest that phloretin inhibits cancer cell growth in a p53-dependent manner and causes cell cycle arrest.^942–944^ It also increases cancer cell sensitivity to chemotherapy by impeding GLUTs.^945^ Moreover, GLUT2 activation influences p53 signaling, affecting cell cycle control and apoptosis.^946^ Ritonavir, an antiviral drug approved by the FDA, has been identified as a GLUT4 inhibitor in multiple myeloma, highlighting its potential for new cancer treatment strategies.^947,948^

In summary, GLUT inhibitors represent a promising avenue for cancer therapy. By meticulously targeting cancer cell energy pathways, these inhibitors offer the potential for more effective and selective treatments. Future research should aim to further elucidate the mechanisms and applications of GLUT inhibitors in clinical settings to improve cancer treatment outcomes.

#### HK2

HK2, which is essential for initiating glycolysis, is frequently overexpressed in various cancers. In addition to its glycolytic role, HK2 associates with mitochondrial proteins, sustaining their function and influencing apoptosis.^949^ This makes it an appealing target for cancer therapies.

HK2 inhibitors present new potential in cancer treatment. Notably, 2-deoxy-D-glucose (2-DG) and 3-bromopyruvate (3-BP) are promising HK2-targeting agents.^950^ As a glucose analog, 2-DG is incorporated into glycolysis but halts further metabolism due to structural modifications, blocking glucose conversion to G6P, thereby decreasing ATP production and inducing cancer cell death.^951–954^ Despite inhibiting growth in multiple cancer cell lines^955^ and undergoing early clinical trials for certain cancers (NCT 00633087), its development has been curtailed by toxicity concerns.

3-BP has shown robust anticancer activity over the past twenty years by detaching HK2 from mitochondria, thereby reducing ATP levels and promoting cancer cell death. However, clinical trials of 3-BP in cancer therapy have not yet been sanctioned.^956–959^ A recent breakthrough introduced benzyl nitrile benzohydrazone (BNBZ) as a new HK2 inhibitor. By targeting HK2, it effectively inhibits pancreatic cancer growth and promotes apoptosis, providing valuable insights into HK2 inhibition. Overall, HK2 inhibitors represent promising avenues for cancer therapy. Overcoming existing challenges and advancing our understanding of HK2 mechanisms could lead to more refined and effective cancer treatment options.

#### PKM2

Pyruvate kinase, particularly PKM2, plays a vital role in cancer metabolism by converting phosphoenolpyruvate to pyruvate and producing ATP. While PKM1 is prevalent in normal tissues, PKM2 is highly expressed in tumor cells, making it an appealing target for cancer therapies.^960^

Preclinical studies have shown that metformin enhances chemotherapy sensitivity by disrupting the HIF-1α/PKM2 pathway, inducing apoptosis, and preventing epithelial‒mesenchymal transition.^961–963^ The anticancer potential of metformin, mainly through AMPK activation, is under investigation in numerous clinical trials. In addition to metformin, other agents, such as benserazide—used for PD—exhibit antitumor effects linked to PKM2 inhibition in melanoma cells.^964^ Natural compounds such as shikonin, resveratrol, vitamin K, and gliotoxin inhibit PKM2, exhibiting substantial anticancer activities. Shikonin, derived from Lithospermum, suppresses PKM2 activity and is effective against various cancers, including bladder and lung cancers.^963,965–967^ Resveratrol decreases PKM2 levels, increasing apoptosis by increasing ER stress and mitochondrial dynamics.^968^ Vitamins K3 and K5 hinder glycolysis by reducing PKM2, thus lowering HeLa cell viability,^969^ and they inhibit tumors *via* multiple pathways.^970–972^ Gliotoxin interferes with cancer cell growth by directly targeting PKM2. Compound 3k (C3k) selectively inhibits PKM2, demonstrating significant anticancer effects in cells with high PKM2 expression.^973^ Other inhibitors, such as TEPP-46 and mitapivat, have further expanded therapeutic options.^974^ In summary, PKM2 inhibitors and activators present promising avenues for cancer treatment. Understanding the role of PKM2 may lead to the development of targeted therapies that improve patient outcomes.

#### LDH

LDH plays a vital role in cancer metabolism, especially in advanced stages where elevated lactate levels are common. The isoenzyme LDHA facilitates the conversion of pyruvate to lactate, regenerating NAD^+^ from NADH. This process sustains glycolysis and creates an acidic TME associated with metastasis, recurrence, and poor prognosis.

Targeting LDHA is becoming a promising cancer treatment approach. Oxamate, a pyruvate analog, competes for the active site of LDH, displaying antitumor activity in diverse cancer cell lines, such as gastric, medulloblastoma, cervical, liver, and non-small cell lung cancers.^975–979^ Gossypol, a compound from cottonseed, inhibits LDHA by competing with NADH, offering anticancer benefits.^980^ It also affects Bcl-2 proteins, induces cell cycle arrest, and promotes autophagy. FX11, a small molecule that competes with NADH, has antitumor effects on the gallbladder, prostate, and neuroblastoma, reducing ATP and inducing oxidative stress, effectively inhibiting lymphoma and pancreatic cancer progression.^981–984^ Galloflavin targets both LDH isoforms by binding directly to the enzyme, resulting in in vitro antitumor effects across various cancer cell lines.^985–988^ When metformin is used, it enhances treatment effects against pancreatic ductal adenocarcinoma, opening new strategies for tackling solid tumors and metastasis.^989^ Additional LDHA inhibitors, such as GNE-140, NCI-006, and GSK 28387808, are being studied.^974^

With a deeper understanding of the role of LDH in cancer, LDHA inhibitors are paving the way for innovative cancer therapies. By employing various mechanisms—competing at active sites with cofactors or altering enzyme binding—these inhibitors hold promise for more targeted and effective cancer treatments.

#### Monocarboxylate transporter (MCT)

Monocarboxylate transporters (MCTs), especially MCT1, play key roles in cellular metabolism and energy balance. High MCT1 levels in tumors are associated with greater invasiveness and a worse prognosis. MCT1 supports lactate transport, helping tumor cells thrive in acidic conditions and thereby fostering growth and metastasis. AZD3965, a selective MCT1 inhibitor, shows antitumor activity in vitro against diffuse large B-cell lymphoma, non-Hodgkin lymphoma, and Burkitt lymphoma cell lines. By blocking lactate transport, AZD3965 increases lactate inside cells, inhibiting their growth. Its effectiveness was proven in vivo with a Raji xenograft Burkitt lymphoma model.^990^ It also shows strong cytotoxicity in MCT1-positive, MCT4-negative cells and enhances effects when combined with the GLS1 inhibitors doxorubicin and rituximab.^990^ AZD3965 is currently in phase I trials for solid tumors and lymphomas (NCT01791595). BAY-8002, another MCT1 inhibitor, reduces cell proliferation and tumor size in Daudi and Raji cells but may cause resistance due to increased MCT2 and MCT4 expression.^991^ Despite the good tolerance of advanced tumors to AZD3965, its development has paused. However, recent studies suggest that combining lactate-targeting methods, including MCT4 inhibitors, with immunotherapy may offer new treatment options.^992,993^ Future work should explore the roles and clinical uses of MCT1 inhibitors and their potential synergistic combinations with other therapies.

#### SLC1A5

SLC1A5, or ASCT2, is essential for the uptake of glutamine, a vital nutrient for cancer cell proliferation. Its overexpression in tumors positions SLC1A5 as a promising target for cancer therapies.^994,995^

Early inhibitors, such as V-9302 and the monoclonal antibodies KM 4008 and KM 4012, aimed to block SLC1A5. L-γ-Glutamyl-p-nitroanilide (GPNA) mimics glutamine to disrupt SLC1A5 and Na^+^-dependent amino acid transport, inhibiting glutamine absorption and mTOR pathway activation.^996^ GPNA effectively reduces cancer cell growth in vitro and in vivo, affecting lung cancer, neuroblastoma, prostate cancer, multiple myeloma, breast cancer, and endometrial cancer.^997–1003^ Research suggests that V-9302 might influence other transporters, such as SNAT2, SLC38A2, LAT1, and SLC7A5, rather than directly targeting SLC1A5,^1004^ indicating that further study is needed to clarify its mechanisms and targets. In addition to V-9302 and GPNA, compounds such as benzylserine and benzylcysteine also inhibit SLC1A5 by competing for glutamine binding sites, displaying antitumor activity in breast, endometrial, and gastric cancers.^1003,1005–1007^ Importantly, V-9302 enhances T-cell-mediated antitumor responses in triple-negative breast cancer models.^1008^ In lung and colorectal cancer cells, V-9302 is associated with NF-κB-mediated PD-L1 upregulation, potentially affecting tumor immune evasion.^1009^

Despite some uncertainties, these inhibitors show promise because they target cancer cells in a glutamine-dependent manner. Continued research should clarify their mechanisms, selectivity, and combination potential to increase cancer treatment efficacy.

#### GLS

GLS converts glutamine into glutamate, fueling tumor cell growth and survival, making GLS inhibitors a key focus in cancer treatment research. Inhibitors such as BPTES, CB-839, and compound 968 act allosterically, each with unique modes of action. BPTES has antitumor effects on several cancers, such as breast cancer, lymphoma, glioma, pancreatic cancer, non-small cell lung cancer, and renal cancer.^1010–1015^ However, its clinical potential is limited by its low solubility and bioavailability.^1016^ CB-839, a BPTES derivative, has overcome these limitations and has received FDA approval. It is undergoing clinical trials for both blood-related and solid tumors, alone and with other anticancer drugs.^1017,1018^ These trials examine combinations such as everolimus for renal cancer (NCT 03163667), talazoparib or palbociclib for solid tumors (NCT 03875313, NCT 03965845), paclitaxel for triple-negative breast cancer (NCT 03057600), azacitidine for myelodysplastic syndromes (NCT 03047993), and cabozantinib for metastatic renal cancer (NCT 03428217). While some trials fell short of expectations, CB-839 is still being studied in combination therapies.

Compound 968, which targets Rho GTPase, disrupts glutamine metabolism, thereby inhibiting tumor cell growth and invasion and shrinking tumors in animal models.^1019^ It increases the sensitivity of specific cancers, such as non-small cell lung cancer and ovarian cancer, to chemotherapy.^1020^ Sirpiglenastat (DRP-104) is another glutamine analog under early clinical trials aimed at treating advanced cancers either as a standalone or with immune checkpoint inhibitors (NCT 04471415 and NCT 06027086).^68^

In addition to GLS inhibitors, other factors related to glutamine metabolism are promising targets for cancer therapy. For example, transaminase inhibitors such as amino-oxyacetic acid (AOA) have demonstrated efficacy in reducing tumor activity in laboratory and animal studies.^1021^ Overall, targeting GLS and associated metabolic pathways offers new avenues for cancer treatment strategies.

#### FASN

FASN is a promising target for cancer therapy because it is overexpressed in various cancers. Preclinical research has shown that FASN inhibitors, such as C93 and FAS31, can significantly suppress tumor growth in small lung cancer and melanoma models.^1022,1023^ Newer inhibitors such as TVB-316 and TVB-2640 have shown reduced toxicity and are currently in trials for non-small cell lung cancer, prostate cancer, and HER2-positive metastatic breast cancer (NCT 03808558, NCT 03179904, and NCT 05743621).^1024^ Additionally, the novel compounds TVB-3664 and TVB-3166 demonstrated antitumor effects in laboratory and animal studies, but further clinical trial data are needed.

Orlistat, which is known for its ability to treat obesity, inhibits fat absorption by targeting lipases and acts against FASN, indicating that it is effective against a range of cancers.^1022,1025–1030^ Cerulenin, another FASN inhibitor, interacts with the β-ketoacyl synthase domain, but its reactive epoxy group raises safety issues. This prompted the creation of C75, a more stable alternative that targets multiple FASN sites, despite retaining side effects similar to those of cerulenin.^1031–1034^ FASN continues to be a critical focus for new cancer therapies, with research efforts geared toward maximizing efficacy while minimizing adverse effects.

#### ATP citrate lyase and ACC1 and ACC2

ACLY is a vital enzyme in cellular metabolism that is targeted by several inhibitors. Bempedoic acid, a notable ACLY inhibitor, effectively reduces lipid levels and received FDA approval in 2020 as a cholesterol-lowering drug.^1035^ In ACLY inhibitor research, NDI-091143 stands out for its strong nanomolar inhibitory activity as an allosteric inhibitor. This compound binds specifically to the homotetramer of ACLY, hindering its catalytic function. NDI-091143 competes with citrate and simultaneously inhibits ATP, allowing precise modulation of ACLY activity.^1036^

ACC1 and ACC2 regulate cellular fatty acid synthesis. The novel oral inhibitor PF-05221304 is under clinical evaluation for treating nonalcoholic fatty liver disease (NAFLD) and fibrosis (NCT 03248882). While promising for liver disease, its efficacy and application in cancer therapy need further exploration. ND-646, an allosteric inhibitor of ACC1 and ACC2, has been shown to suppress tumor growth in non-small cell lung cancer (NSCLC) xenograft models.^1018^ These findings position ND-646 as a promising candidate for cancer therapy, emphasizing its potential utility in oncology.

#### Carnitine palmitoyltransferase 1

CPT1, an enzyme on the mitochondrial outer membrane, is key to FAO. Inhibitors such as etomoxir hold promise in cancer treatment, showing antitumor effects in prostate and nasopharyngeal cancers, acute myeloid leukemia, and breast cancer.^727,1037–1039^ Etomoxir also works synergistically with CD47 antibodies and radiotherapy.^1040^ ST1326, a selective CPT1A inhibitor, has strong antitumor activity in Burkitt lymphoma and leukemia cell lines, increasing the effectiveness of the Bcl-2 inhibitor ABT-199.^1041–1045^ A limited number of studies have shown that oxfenicine, another CPT1 inhibitor, has anticancer activity in malignant melanoma cells.^1046^ Avocatin B, a natural CPT1 inhibitor from avocados, inhibits AML cell growth without harming normal hematopoietic stem cells, suggesting a potential new approach for blood cancer treatment.^1047^ Perhexiline, initially developed for angina as an inhibitor of CPT1 and CPT2,^1048,1049^ may also reduce cancer cell viability in chronic lymphocytic leukemia, as supported by animal model studies.^1048^

#### Isocitrate dehydrogenase

IDH mutations are pivotal in various cancers. Agios Pharmaceuticals’ ivosidenib (Tibsovo®) targets these mutations and has received FDA approval to treat AML and cholangiocarcinoma with IDH1 mutations.^1050^ Ivosidenib is being tested in trials for combination therapies with other cancer drugs, including a phase III trial with azacitidine (NCT 03173248), a phase I trial with cytarabine and fludarabine (NCT 04250051), and a phase I/II trial with venetoclax (NCT 03471260). It is also in a phase III trial for advanced cholangiocarcinoma (NCT 02989857).

Other IDH1 inhibitors, such as BAY 1436032, olutasidenib (FT-2102), and IDH-305, are under development. BAY 1436032, an allosteric oral inhibitor, penetrates the blood‒brain barrier,^1051^ and although its AML phase I results have shown limited responses, trials for gliomas continue (NCT 02746081). Olutasidenib is undergoing phase I/II trials for AML and myelodysplastic syndromes (NCT 02719574),^1052^ including advanced solid tumors and gliomas (NCT 03684811). IDH-305 is in phase I trials for malignancies with IDH R132 mutations (NCT 02381886). Enasidenib (Idhifa®) is an IDH2 inhibitor for the R140Q and R172K subtypes. It lowers serum 2-HG by inhibiting the α-KG conversion of mutant enzymes,^1050^ which has been FDA-approved since August 1, 2017, for relapsed/refactory AML with IDH2 mutations.^1053^ AGI-6780, another IDH2 inhibitor, shows in vitro promise for cell differentiation, although in vivo studies of tumors are lacking.^1054^ AG-881 is currently in clinical trials for AML patients with IDH2 or mixed IDH1/2 mutations (NCT 02492737).

#### ETC

The ETC, comprising four complexes (CI-IV), is vital for drug development, particularly in cancer therapy. Targeting these complexes provides innovative treatment approaches. Metformin, which is recognized for its ability to inhibit complex I, is used to treat T2DM and has shown anticancer potential in laboratory and clinical settings. In contrast, IACS-010759, a targeted complex I inhibitor, is in clinical trials for acute myeloid leukemia (AML) and advanced cancer.^1055^ While rotenone and deguelin also inhibit complex I, which has anticancer potential, their neurotoxic effects restrict their clinical adoption.^1056,1057^ Alpha-tocopheryl succinate (α-TOS) inhibits complex II, inducing cytotoxicity in tumor cells by promoting electron leakage and ROS generation.^1058^ Similarly, complex III inhibitors, such as benzyl isothiocyanate, trigger ROS accumulation and apoptosis in breast, liver, and lung cancer cells.^1059^ For complex IV and ATP synthesis, inhibitors such as resveratrol and oligomycin have shown promising antitumor activity in skin cancer, neuroblastoma, and lymphoblastic leukemia models.^1057,1060^

#### Dietary interventions in cancer treatment

Specific diets hold significant potential in preventing tumor onset, delaying tumor growth, and enhancing the efficacy of current cancer treatments. Given the heterogeneity of cancer and host metabolism, some diets are tailored to target specific vulnerabilities. Caloric restriction, ketogenic diet, and intermittent fasting are among the dietary interventions predominantly employed in cancer therapy research. Since cancer cells typically rely on glucose as their primary nutrient for energy production, restricting glucose intake is a promising therapeutic approach. Limited glucose intake redirects the body towards the utilization of ketone bodies, resulting in beneficial effects in cancer treatment with the ketogenic diet.^1061^ Owing to the high demand of cancer cells for protein synthesis, restricting essential amino acid intake in the diet can inhibit tumor growth.^1062^ However, the role of protein intake in cancer initiation and progression remains debatable. Cancer cells often exhibit high expression of lipid receptors and transport proteins. It has been demonstrated that diets rich in lipids derived from the diet and adipocytes contribute to tumor progression and metastasis.^1063,1064^ Therefore, limiting lipid elevation may have anti-tumor effects, but conclusive data from more preclinical models are needed. Importantly, dietary manipulations induce systemic responses that are not limited to the tumor itself and influence other stromal behaviors, such as the immune system and overall homeostasis. Therefore, the impact of dietary restrictions should be viewed holistically with the aim of maintaining functional anti-tumor immune responses and avoiding cachexia development. Dietary manipulation in cancer therapy may be short-term and should be combined with other treatment modalities to increase patient compliance.

#### Metformin in cancer treatment

Metformin demonstrates a significant role in energy regulation in cancer treatment, exhibiting certain inhibitory effects on various tumors. One of its primary mechanisms of anticancer activity involves the inhibition of complex I in the cell’s mitochondrial respiratory chain, thereby activating AMPK.^1065^ Activated AMPK not only exerts anticancer effects by inhibiting the mTOR signaling pathway but also suppresses fatty acid synthesis, effectively inhibiting cancer cell proliferation.^1066^ Additionally, metformin may induce intracellular and extracellular acidification by regulating lactate metabolism.^1067^ This acidification of the TME disrupts NAD^+^ regeneration, inhibiting cancer cell proliferation.^1068^ Metformin also reduces insulin-like growth factor (IGF) signaling pathway activity, decreases glucose absorption, and promotes glucose breakdown, thus reducing energy supply and growth of cancer cells.^1069^ In adjunctive therapy, the combined application of metformin with chemotherapy aids in enhancing the sensitivity of patients to chemotherapy. This effect may be attributed to the blood glucose-lowering effect of metformin, which maintains a glucose-deprived environment unfavorable for cancer cell growth, thus increasing the efficacy of chemotherapy.^1070^ Overall, the mechanisms of action of metformin in cancer treatment are diverse and complex, with the potential to inhibit tumor growth and metastasis. Comprehensively, the combined application of metformin with other treatment modalities in cancer treatment may yield superior therapeutic outcomes compared with standalone approaches.

#### Brown and beige adipose tissue in cancer treatment

BAT has recently emerged as a potential target in cancer therapy. Studies have shown that cold-induced brown adipose heat production can inhibit tumor growth.^1071^ Both BAT and tumor cells rely on glucose as their primary energy source. The activation of BAT under cold conditions increases the demand for glucose, potentially limiting the uptake of glucose by tumor cells, thereby inhibiting their growth.^1072^ Brown adipocytes can efficiently utilize glucose and other nutrients to produce heat rather than ATP, which aids in suppressing tumor growth.^1073^ Cancer cells often alter metabolic pathways to support their rapid proliferation. Activating brown adipose tissue may alter the overall metabolic state, influencing the metabolic pathways of tumor cells and thereby inhibiting their growth. This metabolic regulation could offer novel insights and approaches for cancer therapy. Therefore, studying and harnessing the anti-tumor effects of BAT may provide new research avenues for cancer treatment, offering more treatment options and opportunities.

Although metabolic enzymes present attractive therapeutic targets for cancer treatment, various targeted drugs are at different stages of clinical trials for multiple reasons. Only a few have been approved by the FDA for cancer therapy. Nucleoside analogs were among the first chemotherapeutic drugs introduced for cancer treatment; however, they not only affect cancer cells but also impact normal proliferating cells. Similarly, targeting other metabolic complexes or enzymes is restricted by their toxicity to normal tissues. Additionally, the metabolic plasticity of cancer cells, where cells can upregulate alternative pathways or acquire nutrients from the environment to adapt to metabolic changes, poses a challenging task that will require simultaneous targeting of metabolic pathways and nutrient clearance routes.^1074^ The focus of future studies on immunometabolism will be to enhance the understanding of the multifaceted functions of complex immune metabolic signaling pathways within the TME, develop highly effective targeted drugs that combine specificity and safety, or improve cancer immunotherapy in combination with immune checkpoint inhibitors (ICIs) to enhance resistance. The identification of specific, mutation-dependent metabolic vulnerabilities in particular cancers by targeting them to synergize with radiotherapy, chemotherapy, or immunotherapy to induce cytotoxicity is suggested.

### Targeting energy metabolism for neurodegenerative disease therapy

Disruptions in energy metabolism, particularly in glycolysis and mitochondrial processes, are central pathogenic mechanisms in neurodegenerative diseases. Targeting these metabolic pathways has shown therapeutic potential in treating these conditions (Fig. 10 and Table 3). Although a decrease in cerebral glucose metabolism has been widely observed in both clinical and preclinical trials of NDs, this reduction in glucose metabolism appears to be unrelated to circulating glucose levels but rather associated with increased blood sugar, such as in diabetes. Therefore, merely supplementing glucose is not a feasible strategy for treating ND; enhancing mitochondrial energy metabolism and improving impaired cerebral basal metabolism may be a more effective treatment approach.

**Table 3:** Pharmacological treatment of cardiovascular diseases

Drug	Mechanism	Results	Action
Perhexiline/ Etomoxir	Inhibit CPT1	Inhibit FAO, enhance glucose oxidation	Improve myocardial ischemia and heart failure
Trimetazidine	Block Long-chain 3-ketoacyl CoA Thioesterase	Inhibit FAO, increase glucose oxidation, Improve insulin sensitivity	Improve myocardial ischemia
Trimetazidine	Upregulate the expression of AMPK and PPAR-α	Facilitate the absorption of energy substrates and protein expression, especially ketones	Improve heart failure
Trimetazidine	Activate the AMPK/ERK pathway	Inhibiting FAO to enhance glucose oxidation	Reduce reperfusion injury
Trimetazidine	Activate the SIRT1-AMPK pathway	Enhance ATP production and SOD activity, while decreasing LPO, FFA, and NO levels	Improve myocardial infarction
Glucose-Insulin-Potassium (GIK) Solution	Reduce circulating free fatty acid levels	Inhibit FAO, Enhance Glycolysis	Reduce myocardial infarct size
Lipstatin-1	Reduce ROS	Reduce lipid peroxides	Reduce myocardial infarct size and ischemia-reperfusion injury
Simvastatin/Fluvastatin	Block the MVA pathway	Reduce lipid peroxides	Lower cholesterol levels, maintain normal heart function
Simvastatin	Activating the JAK/STAT3 pathway	Alleviate mitochondrial damage	Improve heart failure
Coenzyme Q10	Clear free radicals	Enhance mitochondrial energy production	Improve myocardial ischemia
Ferrostatin-1	Reduce ROS, prevent lipid peroxidation	Reduce iron death, maintain mitochondrial function	Protect myocardial cells
Mitotane	Reduce ROS	Reduce lipid peroxides	Rescue from DOX-induced cardiomyopathy
Dexrazoxane	Reduce ROS	Reduce lipid peroxides	Maintain mitochondrial function
Fenofibrate	Activate PPAR-α	Promote FAO, regulate cardiac energy metabolism, alleviate oxidative stress	Improve heart failure
Dichloroacetic Acid (DCA)	Inhibit PDK	Enhance glucose oxidation and reduce glycolysis	Reduce reperfusion injury
Carvedilol	Improve insulin resistance, reduce oxidative stress.	Enhance glucose oxidation	Improve heart failure
Pioglitazone	Activate PPAR-γ, Anti-inflammatory	Alleviate mitochondrial damage	Reduce reperfusion injury
Metformin	Activate AMPK	Increase glucose uptake, improve insulin resistance	Improve diabetic cardiomyopathy
Metformin	Up-regulate Sirt3, reduce the acetylation level of PGC-1a	Inhibit mitochondrial damage, improve mitochondrial respiratory function	Improve heart failure
Empagliflozin	Reduce pACC, CPT1, CD36; Enhance GLUT4	Block FAO, increase glucose uptake	Improve diabetic cardiomyopathy
SGLT2 Inhibitors	Increase levels of beta-hydroxybutyrate in the blood	Decrease energy demand, stabilize mitochondria	Improve energy metabolism in heart failure

#### Targeting glycolysis and the TCA cycle

In neurodegenerative diseases, the downregulation of PGK1, an essential glycolytic enzyme, is prevalent. Activating PGK1 presents a potential treatment strategy. Terazosin enhances PGK1 activity, increasing glycolysis and ATP levels in the brain to address energy deficits in mice.^343,1075^ Supplementing with pyruvate, a key link between glycolysis and the TCA cycle, improves brain energy status.^1076,1077^ Ketone bodies such as β-hydroxybutyrate and caprylic acid increase TCA cycle activity by increasing acetyl-CoA levels, independent of glycolysis.^1078,1079^ These ketones, which are transported to the brain, increase TCA efficiency.^1080^ Triheptanoin compensates for low levels of BCAAs in Huntington’s disease (HD), addressing energy metabolism problems.^1081,1082^ Tributyrin formulations improve mitochondrial function and ketone availability, supporting glucose metabolism in AD.^1083^ The specific effects of ketone body intervention on AD, and its potential relationship with brain lipid distribution and lipid metabolism, merit further investigation. Furthermore, while ketone supplementation has been effective in experimental animal models, its impact in clinical trials has not been as pronounced,^1084^ warranting further research on its long-term effects on a broader population of individuals with AD. This variability in response may be attributed to individual metabolic differences, emphasizing the importance of personalized treatment approaches.

Diabetes poses a risk for neurodegenerative diseases, and diabetes treatments may aid in disease management. Metformin increases ATP production *via* glucose metabolism regulation, potentially easing ND symptoms.^1085^ Semaglutide, a GLP-1 agonist, offers neuroprotection in PD and AD by increasing autophagy, reducing apoptosis, and reducing α-Syn and Aβ toxicity.^1086–1088^ The further development of complex multi-target drugs that simultaneously possess neuroprotective potential and target specific shared pathways in T2DM and AD holds promise as potential therapeutic interventions.

#### Mitochondrial targeting in neurodegenerative diseases

Mitochondrial abnormalities, such as decreased respiratory capacity, increased mitochondrial fragmentation, and imbalanced mitochondrial fission/fusion, have been identified in the brains of individuals with AD prior to the deposition of pathological β-amyloid plaques.^1089^ Thus, mitochondrial dysfunction plays a critical role in neurodegenerative diseases.^1090^ Research efforts have aimed to increase mitochondrial function by supporting the ETC, stimulating biogenesis, and reducing oxidative damage.^1091^ Coenzyme Q10 provides neuroprotective effects by enhancing mitochondrial function, but this is only effective in patients with a mitochondrial type of PD. In future treatments, a potential direction could be to assess the risk of mitochondrial dysfunction more accurately for targeted therapy. CP2, through partial inhibition of complex I, enhances mitochondrial energy efficiency, potentially increasing ATP levels in the brain.^1092^ It also activates AMPK, increasing neuronal protection against oxidative stress, reducing tau and Aβ accumulation, and improving axonal transport.^1092,1093^

Therapeutic approaches include targeting estrogen receptor-β with (S)-equol to increase cytochrome c oxidase activity in AD.^1094^ Uncoupling proteins offer new ways to bolster the cellular defense against oxidative stress. Selective inhibition of complex I and targeting of these proteins may enhance ND treatment outcomes.^1094^ Low-dose 2,4-dinitrophenol has shown promise in ND experimental models.^1095^ Dapagliflozin safeguards mitochondria in diabetic mouse models, normalizing their size and reducing oxidative damage.^1096^

Mitochondrial biogenesis, which is crucial for cellular health, is promoted by resveratrol *via* the SIRT1-AMPK-PGC-1α pathway, which also enhances autophagy to clear damaged mitochondrial components.^132,1097^ The inhibition of mitochondrial fragmentation is beneficial in ND contexts.^1098^ Quinazolinone derivatives such as mdivi-1 inhibit excess division, offering neuroprotection in AD, PD, and brain injury.^1099,1100^ EPA, an ω-3 fatty acid, also supports mitochondrial integrity by altering lipid composition.^1101,1102^

Maintaining redox balance is targeted by MitoQ, which reduces oxidative stress and offers neuroprotection in animal models of ND.^1103^ However, clinical trials have not demonstrated neuroprotective effects in PD patients, further emphasizing the differences between animal models and clinical diseases. Altering the NAD^+^/NADH ratio enhances brain energy status; NR, a precursor to NAD^+^, alleviates cognitive issues in AD models.^272^ Metformin and pioglitazone aid in mitochondrial health; hydralazine increases activity, indicating therapeutic potential.^1104^ Natural antioxidants such as alpha-tocopherol, curcumin, resveratrol, quercetin, and rosmarinic acid are also highlighted as potential therapeutic strategies to counteract oxidative damage and ROS production in AD.^1105^ However, the specific protective mechanisms of these drugs still require further investigation, including their impact on mitochondrial complexes, modulation of the calcium ion balance, and stabilization of mtDNA.

Clearing dysfunctional mitochondria is key to maintaining cellular metabolism. Insulin is crucial for regulating glucose metabolism and mitochondrial dynamics.^1106^ Urolithin A promotes mitophagy, reduces inflammation, and improves cognition.^1107,1108^ PINK1 activators such as MTK458 facilitate mitophagy, suggesting therapeutic avenues for PD.^1109^

#### RNA and DNA therapeutics

Recent advancements in oligonucleotide therapies have shown significant clinical success,^1110,1111^ particularly with the development of antagomirs and locked nucleic acids, which enhance the relevance and efficacy of oligonucleotides in treating neurodegenerative diseases.^1112,1113^ In Huntington’s disease, antisense oligonucleotides target mutant HTT mRNA to prevent disruptions in mitochondrial transport and function.^1114^ Allele-specific approaches reduce mutant HTT while preserving normal HTT expression, offering new treatment avenues. Similar strategies are being explored for PD and amyotrophic lateral sclerosis (ALS) with frontotemporal dementia (FTD), addressing mitochondrial gene mutations.^1115,1116^

Oligonucleotides and small interfering RNAs (siRNAs) modulate pre-mRNA splicing or neutralize microRNAs, increasing the expression of energy-producing genes that are often downregulated in neurodegenerative conditions.^1111,1117^ Direct gene therapies involve the delivery of full gene copies to restore deficient expression, as observed with the use of AAV vectors for spinal muscular atrophy.^1118^ Strategies targeting dysfunctional PGC-1α may restore mitochondrial functions in DA pathways in PD models.^1119^ DNA and RNA editing technologies such as zinc finger nucleases and CRISPR–Cas9 show therapeutic promise. In AD, converting ApoE4 alleles to ApoE3 through gene editing has demonstrated potential in improving brain energetics.^1117,1120^ Although in vivo APOE editing is nascent, rapid advancements are underway to neutralize ApoE4 and improve AD-related glucose metabolism.^1121^ HD-linked mitochondrial dysfunction due to excessive mRNA translation highlights a new therapeutic target, with complexes that inhibit translation offering a path to restore mitochondrial energy integrity.^1122^ Autophagy-targeting chimeric molecules eliminate dysfunctional mitochondria, restoring mitochondrial function and ATP levels in Down syndrome fibroblasts, potentially preventing AD progression.^1123^ However, the aforementioned studies were primarily conducted in mouse models, and the clinical outcomes have been consistently unsatisfactory. This discrepancy may stem from the fact that disease progression in clinical patients is more severe than that in experimental models, rendering treatments less effective. Nevertheless, further research on mitochondrial gene therapy remains crucial.

#### Dietary interventions

Diet plays a crucial role in enhancing energy metabolism in neurodegenerative diseases. As mentioned earlier, ketones can serve as a vital energy source for neurons, with dietary control being an effective strategy to intervene in ketone metabolism. A ketogenic diet is crucial for improving neuroinflammation by inhibiting glycolysis and promoting ketone body production. Ketone bodies regulate insulin secretion by inhibiting glycolysis, enhancing insulin sensitivity, and improving glucose tolerance.^1124^ They also ameliorate mitochondrial dysfunction in neurons and glial cells.^1125^ The antioxidative effects of ketone bodies on mitochondrial function occur primarily by regulating mitochondrial respiratory complexes, reducing ROS levels, and enhancing antioxidant capacity.^1126^ Following ketone supplementation, an increase in glutathione levels in hippocampal mitochondria of rats can be observed, which helps protect neurons from damage.^1127^ The supplementation of ketone bodies in AD and PD patients results in improvements in cognitive and motor functions.^1128,1129^ However, further research is needed to understand how to enhance the brain’s absorption and utilization of ketone bodies. The consumption of foods rich in antioxidants, such as vitamin C, vitamin E, and polyphenols, assists in scavenging free radicals, reducing oxidative stress-related damage to neurons. Certain nutrients in the diet, such as tyrosine and tryptophan, serve as precursors for neurotransmitter synthesis, enhancing neurotransmitter release and neural signal transmission. Foods containing polyunsaturated fatty acids and omega-3 fatty acids contribute to improving lipid metabolism and alleviating neuroinflammatory responses. The Mediterranean diet and the Jiangnan diet are rich in fiber, antioxidants, polyunsaturated fatty acids, phenolic compounds, and other beneficial substances, which have positive protective effects on neurons.^1130^

Identifying new vectors, novel therapeutic targets, and reliable transgenic delivery pathways remains a primary focus in research on gene therapy for NDs. While clinical trials related to gene therapy are ongoing, satisfactory outcomes have not yet been achieved. Future research should focus on the cellular and molecular mechanisms associated with neurodegenerative diseases to identify more effective diagnostic and therapeutic targets. Despite promising therapeutic avenues for ND treatment indicated by recent studies, the majority of new treatments have failed in clinical trials. The development of new drugs remains a constant area of research aimed at delaying disease progression and preventing cell death. Personalized treatments focusing on comprehensive metabolomics and genetics are essential for addressing neurodegenerative diseases, considering their prevalence in aging populations and co-occurrence with multiple conditions.

Targeting molecular pathways for populations with metabolic disturbances may be more effective, such as the use of PPAR agonists like pioglitazone, which has shown therapeutic efficacy in clinical trials. Statin drugs, on the other hand, may be more beneficial for populations with comorbidities like hyperlipidemia. Recognizing the limitations of single-target drugs such as antioxidants and neuroprotective agents in improving ND symptoms, there is a continued emphasis on developing new drugs or combination therapies to address multifactorial causes, potentially offering more effective treatments for this disease.

Inflammation and energy metabolism collectively influence disease progression, yet the efficacy of anti-inflammatory drugs in alleviating ND symptoms is limited. Further exploration is needed to understand the role and therapeutic potential of inflammation at different stages of the disease. Understanding the unique nature of the blood-brain barrier is crucial for improving drug permeability and targeting to avoid peripheral organ side effects. Additionally, in addition to targeting neuronal cells, targeting immunometabolic reprogramming in astrocytes to prevent neuroinflammation may present a new avenue for ND treatment, necessitating the differentiation of various cell types within the substance.^1131–1133^

### Targeting energy metabolism for cardiovascular disease therapy

**Fig. 11:**
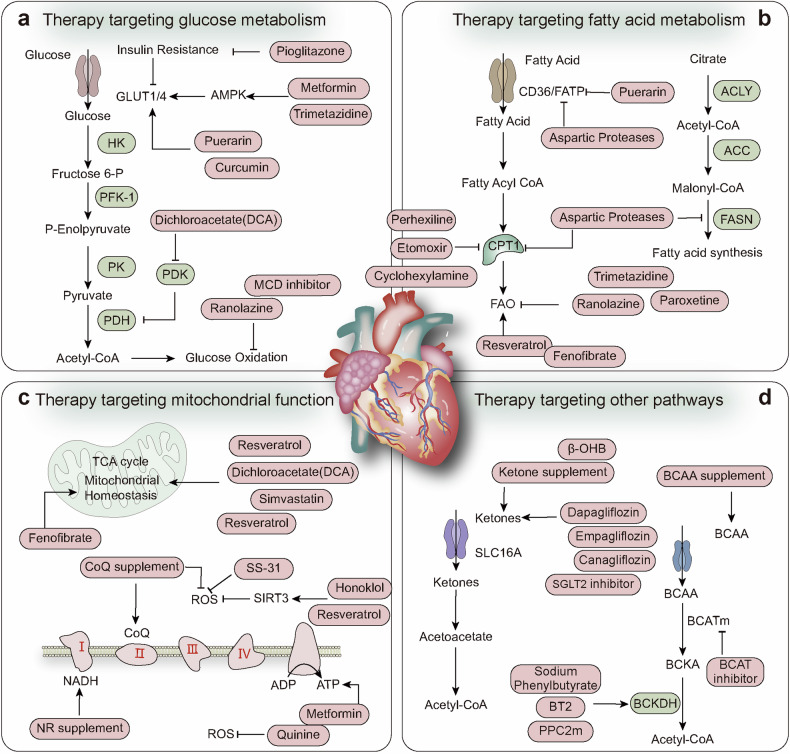
Targeting energy metabolism for cardiovascular disease therapy. **a** Various drugs, by targeting the expression of glucose transport proteins and glycolytic enzymes, can increase energy production in the heart and inhibit the process of oxidative stress. **b** Medications that target FAO inhibit the highly oxygen-consuming process of FAO and synthesis, shifting towards glycolysis, which can maintain the stability of heart function under hypoxic conditions. **c** By targeting mitochondria to maintain the stability of the TCA cycle and the electron respiratory chain, the generation of ROS can be reduced, ATP production can be promoted, and the stability of the heart can be maintained. **d** By supplementing with external energy substances, including ketone bodies and branched-chain amino acids, the energy synthesis and functional stability of the heart can be promoted

Harnessing the modulation of energy metabolism represents a novel therapeutic approach that shows promise for cardiovascular disease management. The study of metabolic interventions has increasingly concentrated on the dynamics of cardiac fatty acid and glucose metabolism, along with mitochondrial oxidative capacity, as pivotal areas impacting cardiovascular health. Strategically adjusting metabolic processes within cardiomyocytes—including the enhancement of glucose oxidation and the reduction of FAO—offers a pathway to bolster the energy provision for heart cells, consequently increasing cardiac pump efficiency (Fig. 11).

#### Targeting glycolysis

Glycolysis has emerged as a pivotal therapeutic target in cardiovascular diseases, serving as a critical energy source for cardiomyocytes adapting to hypoxic conditions. The enhancement of glycolytic pathways holds promise for early intervention. Metformin, by activating AMPK, restores glucose uptake in insulin-resistant myocardial cells, showing efficacy in diabetes-related cardiac issues.^1134^ Pioglitazone improves insulin sensitivity and glucose uptake while preventing mitochondrial dysfunction, mitigating damage during ischemia‒reperfusion.^1135,1136^ Trimetazidine activates AMPK, enhancing mitochondrial function, insulin sensitivity, and GLUT4 translocation and increasing glucose uptake.^1137^ Ranolazine significantly augments glucose oxidation in ischemic and reperfused rat hearts,^1138^ whereas malonyl-CoA decarboxylase inhibitors increase glucose oxidation and improve ischemic cardiac function.^1139^

Notably, trimetazidine shifts cardiac metabolism from FAO to glucose utilization by inhibiting mitochondrial long-chain 3-ketoacyl CoA thiolase.^1140^ Carvedilol optimizes substrate preference from fatty acid to glucose oxidation, reducing oxygen consumption and enhancing efficiency.^1141^ Neuregulin modulates PACC, CPT1, CD36, and PGC-1α expression while increasing GLUT4 transport, optimizing cardiac energy metabolism.^1142^

Natural compounds such as puerarin, curcumin, and resveratrol show therapeutic potential. Puerarin improves postinfarction function in diabetic mice by modulating GLUT4 and CD36 expression and translocation.^1143^ Curcumin activates the SIRT1-Fox1 and PI3K/Akt pathways in diabetic rat hearts, promoting glucose uptake and utilization and inhibiting oxidative stress and apoptosis.^1144^ Resveratrol normalizes FAO, enhances glucose use, and mitigates oxidative stress.^428^ These findings underscore the importance of targeting glycolysis and metabolic modulation in cardiovascular disease management.

#### Targeting mitochondria

Mitochondria, as principal energy sources, play indispensable roles in cardiovascular health by regulating intracellular calcium levels, oxidative reactions, and apoptosis.^1145^ However, their dysfunction is a catalyst for cardiovascular disease progression, as evidenced by significant mitochondrial disruptions during the early stages of these diseases.^1146^ The pathogenesis of cardiovascular conditions is closely linked to oxidative stress, which arises from an imbalance in ROS and is coupled with decreased endogenous antioxidant enzymes.^1147^

Enhanced lipid peroxidation, which leads to vascular membrane damage, is central to cardiovascular disease mechanisms.^1148^ To address such challenges, several mitochondria-protective drugs have been identified. For example, coenzyme Q10 supports mitochondrial integrity by increasing ATP levels, enhancing radical scavenging, and promoting electron transport in the respiratory chain.^1149,1150^ Metformin mitigates oxidative stress, reduces mitochondrial HSCoA depletion, and elevates cardiac HSSA and ATP levels.^1151^

Similarly, simvastatin sustains mitochondrial function by inducing lipid droplet accumulation, facilitating mitochondrial autophagy, and preventing mitochondrial damage.^422^ MCD inhibitors enhance mitochondrial antioxidative capacity by modulating malonyl-CoA levels, consequently reducing FAO and proton generation.^430^ Dichloroacetate (DCA), a PDK inhibitor, enhances Krebs cycle flux, preventing myocardial hypertrophy and restoring contractile reserve without altering the expression of key metabolic regulators.^1152^ Pioglitazone, a PPAR-γ agonist, has the potential to prevent mitochondrial disruption and intramyocellular lipid imbalance, which are crucial for reversing severe pulmonary arterial hypertension and vascular remodeling.^1135^ Nicotinamide riboside (NR), an NAD^+^ precursor, has been demonstrated to protect mitochondrial function in peripheral blood mononuclear cells and reduce inflammation in advanced heart failure patients.^1153^ Fenofibrate contributes uniquely to cardiovascular treatment by enhancing mitochondrial function and FAO and increasing SDH activity and the expression of genes such as PPAR-α and SIRT3.^441^

In addition to synthetic drugs, natural compounds have shown remarkable mitochondrial protective effects. Antioxidants reduce the risk of heart disease through their ability to scavenge free radicals, which inhibits the generation of ROS and enhances endogenous antioxidant enzymes.^1154^ Anthocyanins counteract mitochondrial dysfunction through multiple pathways, including the inhibition of cytochrome C reduction and caspase activation.^1155^ Resveratrol preserves the mitochondrial membrane potential in cardiomyocytes, inhibits apoptosis, increases mitochondrial respiratory enzyme activity, and curtails ROS production.^428^ Increased lipid peroxidation severely impacts the mitochondrial function of cardiac cells by inducing ferroptosis, making the inhibition of ferroptosis a current focus in the treatment of cardiovascular diseases. Treatment strategies primarily involve reducing the extent of lipid peroxidation, decreasing iron ion concentrations, disrupting iron transport, and regulating the expression of ferroptosis-related proteins.^1156^

#### Targeting FAO

Although FAO is a primary energy source, is relatively inefficient because of its high reliance on oxygen molecules during metabolism.^432^ Enhancing metabolic efficiency in the heart is paramount, especially in cardiac diseases where the oxygen supply may be compromised. CPT1 is a key player in mitochondrial fatty acid uptake, and modulating its ability to inhibit fatty acid absorption could redirect cardiac metabolism toward more energy-efficient pathways under pathological conditions. Inhibiting CPT1 with compounds such as perhexiline and etomoxir has been shown to reduce FAO, thereby mitigating heart failure symptoms.^433^ Etomoxir, in particular, inhibits CPT1 and promotes glucose oxidation, conferring cardioprotection against ischemic injury.^433^ In patients with chronic heart failure, CPT1 inhibitors such as perhexiline and etomoxir have been shown to improve the left ventricular ejection fraction.^1157,1158^ These inhibitors optimize cardiac energy metabolism by curtailing long-chain fatty acid uptake and activating glucose dehydrogenase to increase carbohydrate oxidation.^1159^ Notably, etomoxir effectively prevents contractile dysfunction in animal models, likely by enhancing myocardial performance. Perhexiline, a CPT1 inhibitor, induces a metabolic shift from fatty acid to glucose oxidation, triggering a complex rebalancing of carbon and nucleotide phosphate fluxes.^1159^ This metabolic redirection not only optimizes energy production but also enhances metabolic flexibility by increasing lactate and amino acid uptake.^1159^ Furthermore, perhexiline may exert additional effects beyond metabolic redirection, such as reducing ROS production throughout the cardiovascular system by altering the NADH/NADPH ratio.^1160^ Given that ROS are key contributors to cardiovascular damage, this property of perhexiline could have significant implications for the prevention and treatment of cardiovascular diseases.

In addition to limiting lipid absorption, directly inhibiting FAO represents a promising therapeutic approach.^1158^ Trimetazidine, a drug that restricts FAO by blocking the enzyme 3-ketoacyl-CoA thiolase while simultaneously promoting glucose oxidation,^1161^ not only improves the cardiac energy supply but also may regulate cardiac function by reducing systemic resting energy expenditure.^1162^ In patients with dilated cardiomyopathy and myocardial ischemia, trimetazidine has been shown to increase the left ventricular ejection fraction,^1163^ indicating a direct positive impact on cardiac function. Moreover, trimetazidine treatment has been found to attenuate insulin resistance and improve insulin sensitivity in patients with idiopathic dilated cardiomyopathy.^1163^ Improving insulin sensitivity is particularly crucial for diabetic patients, as insulin resistance underlies the pathogenesis of T2DM and is associated with various metabolic disorders. By enhancing insulin sensitivity, trimetazidine may contribute to improved metabolic states in the heart. Abnormal lipid metabolism is closely associated with heart diseases, exacerbating cardiomyocyte death and decreasing bioenergetic production through mechanisms such as the induction of ferroptosis. Ferroptosis is closely associated with other types of cell death, including apoptosis, necroptosis, and necrosis. Future research should focus on understanding how the regulation of this mixed cell death affects cardiac energy metabolism. Past studies have highlighted the roles of ZBP1, AIM2, and other regulators of mixed cell death in heart injury; however, research on how these molecules specifically impact the cardiac energy metabolism process is limited. Investigating the functions of these proteins and identifying additional common regulatory molecules as potential intervention targets are crucial.

#### Dietary interventions

Dietary adjustments play a crucial role in the treatment of cardiovascular diseases. The proportions of fats, proteins, and carbohydrates in the diet directly influence the energy metabolism of individuals with cardiovascular diseases. The intake of antioxidants and unsaturated fatty acids in the Mediterranean diet contributes to improving lipid metabolism, benefiting cardiovascular health.^1164^ Diets high in fiber and low in saturated fats help control chronic inflammation, improve lipid and glucose metabolism, thereby reducing the risk of cardiovascular diseases. Components of the diet, such as fiber and probiotics, play crucial regulatory roles in balancing the gut microbiota. The Jiangnan dietary pattern shares similarities with the Mediterranean diet in terms of nutritional components and plays a positive role in controlling cardiovascular diseases. However, Western HFDs rich in choline can be converted by gut bacteria, leading to elevated levels of trimethylamine N-oxide, consequently promoting the development of cardiovascular diseases.^1165^ Foods rich in antioxidants such as vitamin C, vitamin E, and flavonoids in the diet aid in scavenging free radicals, reducing oxidative stress damage and thereby promoting mitochondrial energy production. A ketogenic diet has a beneficial effect on cardiovascular diseases, primarily because of its anti-inflammatory and antioxidant effects and the provision of alternative fuel for cardiac metabolism.^91^ Moreover, the role of intermittent fasting in improving cardiovascular diseases also relies on ketone generation and the enhancement of risk factors such as obesity and lipid imbalances.^91^

Cardiovascular diseases often involve alterations in lipid and glucose metabolism. Shifting the balance from fatty acid β-oxidation to glucose oxidation to optimize energy substrate metabolism has been suggested as a therapeutic strategy for treating (ischemic) heart disease. However, increased glycolysis may lead to myocardial hypertrophy, necessitating the prioritization of FAO to alleviate cardiac hypertrophy. This underscores the dynamic transitions in heart disease, demanding corresponding changes in treatment approaches. Recent research on cardiovascular disease treatment has focused on various natural or synthetic active compounds that protect mitochondrial function and maintain energy production. These compounds act by reducing ROS, inhibiting abnormal lipid peroxidation, and stabilizing the ETC, with promising results from clinical trials of these active substances.^1166,1167^

Despite significant efforts, there are currently no approved treatments specifically targeting cardiac metabolism. This is partly due to differences in energy metabolism between animal models and human physiology and a lack of reliable non-invasive detection methods. With the increasing prevalence of cardiovascular diseases, more significant challenges are posed for effective preventive and therapeutic strategies, necessitating continued exploration and validation of new biomarkers to enhance early diagnosis of cardiovascular diseases. While advances in metabolomics have led to the discovery of numerous biomarkers, translating metabolomics research findings into diagnostic and therapeutic methods in clinical practice still faces various challenges, including accuracy, cost, and practicality. The development of gene editing technologies like CRISPR/Cas9 offers opportunities to screen potential pathogenic genes and target treatments for certain genetic cardiovascular diseases. However, substantial variations in metabolic levels exist among individuals, highlighting the challenge of studying and understanding the differences in cardiovascular disease metabolic characteristics among various individuals for the personalized treatment and prevention of cardiovascular diseases.

### Targeting energy metabolism for autoimmune disease therapy

Energy metabolism is a critical factor in the pathogenesis of autoimmune diseases, particularly in supporting the proliferation and function of activated T and B cells, which have high energy demands. These metabolic perturbations are not isolated events but are interconnected and interact within the body. The consequence of this interaction is the breakdown of immune tolerance, manifested by the proliferation of follicular helper T (Tfh) and Th17 cells, impaired function of Tregs, aberrant B-cell activation leading to excessive autoantibody production, elevated levels of inflammatory mediators, multiorgan inflammation, and tissue damage, ultimately culminating in the development of autoimmune disorders. Thus, modulating energy metabolism to ensure proper differentiation and function of immune cells is paramount for maintaining immune homeostasis and treating autoimmune conditions.

Current therapeutic approaches for autoimmune diseases include the use of nonsteroidal anti-inflammatory drugs (NSAIDs) to manage pain and inflammation, glucocorticoids (GCSs) to suppress an overactive immune system, and disease-modifying antirheumatic drugs (DMARDs) to inhibit the release of inflammatory mediators and immune cell proliferation (Table 4). Patients diagnosed with autoimmune diseases in the early stages may benefit from these interventions, as they help mitigate inflammatory symptoms and prevent disease progression.^1168^ As research has advanced, the significant connection between these therapeutic agents and the modulation of energy metabolism has become increasingly evident. Ongoing studies targeting energy metabolism are providing novel insights and strategies for the treatment of autoimmune diseases, offering promising avenues for future therapeutic interventions.

**Table 4:** Pharmacological treatment of autoimmune diseases

Drug	Mechanism	Results	Action
CG-5	Inhibit glucose transport	Inhibit Th1 and Th17 differentiation	Alleviate RA
2-DG	Competitive binding HK2	Inhibit immune cell activation	Alleviate arthritis.
Metformin	Activate AMPK, Akt/mTOR	Inhibit FLSs	Alleviate arthritis, SLE
Lonidamine	Inhibit HK1 and HK2	Restore anti-inflammatory macrophage phenotype	Alleviate joint damage
3-Br-PA	Inhibit HK1 and SDH	/	Alleviate arthritis
Repaglinide	Activate mTOR	Promote Treg differentiation and function, inhibit Th17 cells	Alleviate RA
Iguratimod	Inhibit HIF-1α-HK2 axis	Inhibit Tfh cell function	Alleviate RA
MitoTempo	Improve mitochondrial oxidative stress	Reduce neutrophil NETosis	Alleviate SLE
NAC	Boost mitochondrial membrane potential in T cells	Reduce T cell proliferation	Alleviate SLE
BZ-423	Increase ROS and promote apoptosis	Inhibit overactive autoreactive T cells	Alleviate SLE
Tofacitinib	Inhibit JAK-STAT pathway	Inhibit FLSs	Alleviate RA
Cyclosporine	Target MAPK pathway mediated OXPHOS and FAO	Inhibit Th17 cell response, promote Treg cell proliferation	Alleviate RA, SLE
Tacrolimus	Impact ETC complexes II and III to inhibit mitochondrial function	Promote T cell senescence	SLE
Mycophenolate	Promote mitochondrial ROS production	Enhance Treg cell activity	Alleviate SLE
Celastrol	Enhance FAO	Promote Treg cell differentiation	Alleviate RA
Abacavir	Enhance FAO and Foxp3 acetylation	Promote Treg cell differentiation	Alleviate RA

#### Targeting glycolysis

Imbalances in immune cell homeostasis are pivotal in the progression of autoimmune diseases, driving their continuous progression. Adaptations in the local microenvironment lead to a reprogramming of energy metabolism to accommodate these changes. Immune cells exhibit distinct metabolic preferences; for example, in RA, increased glycolysis is evident through elevated GLUT1 activity, yet this pattern differs between CD8^+^ and Tregs.^567,1169^ These cells offset GLUT1 deficits by upregulating GLUT3 or GLUT6,^1169^ whereas Treg cells rely on FAO instead of glucose uptake.^567^ Given these metabolic variations, managing glucose influx via GLUT1 regulation has emerged as a promising therapeutic avenue. The investigative use of CG-5, a broad-spectrum glucose transport inhibitor, highlights its potential in autoimmune therapy by curbing Th1 and Th17 differentiation and diminishing T-cell proliferation in mixed lymphocyte reactions.^1170^

HK, the enzyme that initiates glycolysis through glucose phosphorylation, is competitively inhibited by 2-deoxyglucose (2-DG), which structurally resembles glucose and binds to HK2, thereby hindering its activity through the accumulation of phosphorylated 2-DG.^1171^ Preclinical trials have demonstrated the efficacy of 2-DG in decelerating arthritis in K/BxN mice and suppressing immune cell activation.^1171^ Metformin, a staple in diabetes management, inhibits FLSs *via* the Akt/mTOR pathway, alleviating arthritis symptoms.^1172^ When used alongside 2-DG, metformin enhances IL-2 production in CD4^+^ T cells and mitigates disease symptoms in lupus-prone mice.^1173^ Inhibiting HK1 and HK2 with lonidamine has been shown to alleviate joint damage in collagen-induced arthritis models^1174^ while reducing IL-1β and TNF-α levels and restoring macrophage anti-inflammatory functions in RA models. 3-Bromopyruvate (3-Br-PA) not only affects SDH but also inhibits HK2, showing promise as a novel treatment for inflammatory arthritis.^582^ Its application, alongside FX11, an LDHA inhibitor, effectively decreases lactate production in stimulated synovial fibroblasts, attenuating inflammation in mice.^579,1175^ Rapamycin, which targets mTOR, has been confirmed to aid in Treg development and function in SLE while inhibiting Th17 cell maturation.^1176,1177^ Through dual disruption of glycolysis and glutaminolysis, bioactive compounds from plants, such as C28MS, significantly alleviate arthritis severity, suggesting their potential as RA treatments.^1178^

The metabolic processes underlying anti-inflammatory drug actions in autoimmune disease treatments often involve glycolysis modulation. Among nonsteroidal anti-inflammatory drugs, aspirin dissociates mitochondrial-bound HK2 by disrupting its interaction with VDAC1,^1179^ whereas diclofenac suppresses GLUT1 and HK2 activity, thus reducing glycolysis.^1180^ Methotrexate, a csDMARD, diminishes HK2 and GLUT expression in RA FLSs,^1181^ and iguratimod disrupts the HIF-1α-HK2 axis, compromising Tfh cell function.^1182^ Tofacitinib, a csDMARD, impairs glycolysis in RA-FLSs, decreasing the expression of HK2, GSK3A, LDHA, and HIF1A^1183^ and promoting the dissociation of HK2 from the mitochondria.^1184^

#### Targeting mitochondria

In autoimmune disorders, immune cells such as activated T and B cells often increase their mitochondrial OXPHOS to fulfill the substantial energy requirements for proliferation and immune responses. However, excessive production of ROS can damage mtDNA, proteins, and lipids, leading to cellular dysfunction and intensified tissue inflammation. Thus, targeting mitochondrial processes represents a promising therapeutic strategy.

MitoTempo, a mitochondrion-targeted antioxidant that mimics superoxide dismutase, effectively neutralizes mitochondrial oxidative stress. Its administration has been shown to decelerate disease progression in MRL/lpr lupus mouse models, demonstrating potential for broader clinical applications.^1185^

N-Acetylcysteine (NAC), a robust antioxidant, has shown positive therapeutic effects in SLE patients.^1186^ NAC treatment significantly curtails the production of anti-double-stranded DNA antibodies and reduces T-cell proliferation, which is vital in managing SLE. By increasing the mitochondrial membrane potential in T cells, NAC facilitates apoptosis, aiding in the control of abnormal T-cell activation and proliferation. In CD4^+^ T cells, NAC additionally enhances the expression of immune regulatory markers such as Foxp3 and CD25, potentially modulating immune responses and mitigating disease symptoms.

BZ-423, a 1,4-benzodiazepine derivative, exerts its effects by increasing ROS levels and promoting apoptosis. In SLE mouse models, BZ-423 effectively inhibits hyperactive autoreactive T cells with elevated ATP synthase expression.^1187^ Treatment with BZ-423 converts mitochondrial oxygen to ROS, triggering apoptosis and significantly improving clinical manifestations in SLE models.

Metformin modulates immune responses by activating AMPK and inhibiting mitochondrial complex I activity.^1188^ This action reduces ROS formation, effectively blocking NETosis and IFNa production in SLE.^1189,1190^ Inhibitors of the JAK-STAT pathway, such as tofacitinib, possess strong anti-inflammatory properties, significantly decreasing the mitochondrial membrane potential, mass, and ROS generation in RA synovial fibroblasts.^1183^ These inhibitors affect key mitochondrial genes and increase OXPHOS and ATP production while reducing the expression of genes related to glycolytic pathways and related genes.^1183^

Itaconate, an SDH inhibitor, modulates succinate and inflammatory cytokine levels in activated macrophages,^1191^ showing associations with decreased disease activity and enhanced treatment outcomes in animal models of arthritis.^1192^

#### Targeting FAO

Elevated levels of BAFF have been observed in various autoimmune diseases.^1193,1194^ B cells exposed to high levels of BAFF show enhanced metabolic capabilities and can evade tolerance checkpoints.^1195,1196^ The BAFF-specific monoclonal antibody Belimumab, which inhibits this dysregulated signaling pathway, has been approved as an adjunct therapy for SLE and has demonstrated efficacy in clinical trials, including improvements in B-cell dysfunction.^1197^ While the primary effects of rapamycin are attributed to alterations in T cells, it also inhibits BAFF-mediated mTORC1 signaling in B cells, thereby limiting their proliferation and survival.^1198^

Statins, by competitively binding to the active site of HMG-COA reductase, effectively block the biosynthesis of cellular cholesterol. In the treatment of SLE, statins not only regulate lipid metabolism but also exert beneficial immunomodulatory effects, offering therapeutic advantages to SLE patients.^1199^ Pioglitazone, a PPAR agonist, promotes the functional expansion of dendritic cells in SLE patients by activating AMPK and inhibiting the mTOR1 signaling pathway. These cells express high levels of PPAR receptors, and the effect of pioglitazone has been validated in vitro.^1200^ Furthermore, by enhancing the expression of CD36 and activating FAO, pioglitazone promotes lipid absorption, offering a new perspective on metabolic regulation in SLE patients.^1201^

N-butyldeoxynojirimycin (NB-DNJ) is a compound with lipid metabolism-regulating capabilities that acts through the inhibition of glycosphingolipid (GSL) synthesis. NB-DNJ modulates the function of CD4^+^ T cells from SLE patients by enhancing TCR signaling, as confirmed in vitro.^1202^ Additionally, NB-DNJ reduces autoantibody production in cocultures of B and T cells, suggesting a novel strategy for immunoregulatory treatment in SLE.

#### Dietary interventions

Dietary patterns also play a role in altering metabolic processes in autoimmune diseases. Dietary interventions can modulate blood lipids, benefiting both patients with SLE and patients with RA by reducing disease activity scores.^1203^ Increasing the intake of omega-3 fatty acids in the diet can raise HDL levels and lower triglyceride levels in adolescent-onset SLE patients,^1204^ whereas in adult SLE patients, it leads to an increase in HDL and a decrease in VLDL. Short-chain fatty acids (SCFAs) consumed in the diet promote the expansion of Tregs in the gut, indicating the multifaceted role of SCFAs in regulating T cell differentiation.^1205^ Oral lipid supplements may enhance the effectiveness of conventional therapies by increasing essential fatty acid levels to boost the systemic inflammatory response, potentially relieving joint pain and predicting responsiveness to DMARDs in RA patients.^1206^ The effects of the aforementioned dietary components suggest that the Mediterranean diet and Jiangnan dietary patterns contribute to improving energy metabolism in autoimmune diseases. However, the impact of intermittent fasting on autoimmune diseases remains inconclusive and requires further research,^1207^ especially concerning the role of the ketogenic diet, for which current studies are still very limited.

Various pathways of energy metabolism have emerged as potential regulators of immune cell differentiation and hold promise for treating autoimmune diseases. Inhibiting different energy metabolic pathways can induce metabolic reprogramming, necessitating a shift towards alternative pathways to maintain cell differentiation and function. Therefore, adopting an integrated approach combining metabolomics and proteomics aids in comprehensively understanding how metabolic enzymes and metabolites influence immune homeostasis under both normal physiological and autoimmune conditions. Importantly, key enzymes in metabolic pathways may exhibit isoforms in different tissues and cells, underscoring the importance of identifying isoform subtypes of enzymes in specific T cell subsets associated with different autoimmune diseases.

## Conclusion and perspectives

The precise regulation of energy metabolism is fundamental for maintaining the balance between energy supply and demand within biological systems. This review outlines the well-established roles of energy metabolism in both health and disease, focusing on key processes such as glycolysis, OXPHOS, FAO, and amino acid metabolism. Disruptions in energy metabolism not only drive the abnormal proliferation of cancer cells and synovial fibroblasts but also lead to imbalances in the differentiation of immune cell populations, including Th17 cells, Tfh cells, Treg cells, and macrophages. These disruptions also cause significant changes in the expression of numerous proteins and enzymes involved in energy metabolism. Metabolic signaling pathways, including the mTOR, SIRT, AMPK, HIF, and Myc pathways, play crucial roles in reprogramming cellular metabolism by regulating the balance between anabolic and catabolic processes. These pathways offer valuable targets for developing new therapeutic approaches with reduced side effects and the potential for targeted elimination of pathological cells. However, despite the promising potential of interventions aimed at correcting metabolic dysfunctions, translating these concepts into practical therapies remains challenging.

In neurodegenerative diseases such as AD and PD, abnormalities in energy metabolism result in insufficient glucose uptake and mitochondrial dysfunction, leading to inadequate energy supply and oxidative stress. These disruptions trigger neuronal malnutrition, structural changes, and functional loss. In contrast, cancer cells undergo metabolic reprogramming to enhance glycolysis and glutaminolysis, adapting to hypoxic and nutrient-deprived environments, which promotes tumor proliferation and metastasis. Metabolic alterations in tumor cells also significantly impact immune cells; effector T cells and M1 macrophages increase glycolysis, whereas memory T cells, Tregs, and M2 macrophages primarily rely on FAO. This metabolic shift provides a survival advantage to Tregs in the TME, leading to competition between tumor and immune cells for energy substrates. Modulating energy metabolism presents a new strategy for treating both neurodegenerative diseases and cancer. Improving mitochondrial function and enhancing OXPHOS could help restore the cellular energy balance and slow the progression of neurodegenerative diseases. Conversely, inhibitors targeting key enzymes or transporting proteins in the glycolytic pathway could reduce the energy supply to tumor cells and inhibit tumor growth. Extensive research into drugs that target energy metabolism is ongoing and has shown promising therapeutic effects; however, the efficacy and safety of these approaches require further validation through clinical studies.

Looking ahead, several key areas in energy metabolism research deserve more in-depth exploration. Mechanistically: 1. Focus on the role of inflammation in energy metabolism: The interplay between inflammation and energy metabolism is reciprocal. However, understanding how inflammation either promotes or disrupts energy metabolism processes, as well as how energy metabolism influences inflammation and the regulatory mechanisms required to maintain their balance, necessitates further investigation. 2. Examine energy interactions among different cell types: The exchange of energy between immune cells and the interaction of energy between cancer cells and stromal cells profoundly impact the overall energy balance in the disease environment. Therefore, exploring the mechanisms of this energy transfer is crucial. 3. Emphasizing regulatory mechanisms: The regulation of energy metabolism has been a cornerstone and challenge in research. Future exploration should delve into the dynamic changes in regulatory signals, particularly focusing on the role of epigenetic modifications in metabolic processes. 4. Development of disease models: Many diseases, especially brain tissue disorders, lack reliable experimental models. The emergence of 3D organoids provides an effective means to explore related mechanisms.

Regarding detection approaches: 1. Focus on metabolic heterogeneity among different cells: Current detection methods struggle to accurately differentiate metabolic variances and dynamic changes between different cell types within disease environments. The development of more precise, noninvasive dynamic monitoring techniques is a significant contemporary challenge. 2. Addressing interindividual variability and early diagnosis: Establishing long-term individual health records and tracking systems to identify disease type, stage, and risk prediction markers on the basis of individual characteristics and metabolic changes is essential. 3. Integration of CRISPR-Cas9 gene editing with multi-omics technologies: The integration of CRISPR-Cas9 gene editing technology with mass spectrometry, single-cell metabolomics, spatial transcriptomics, and other multi-omics methods is crucial for identifying metabolic biomarkers and formulating personalized therapeutic algorithms. Recent studies have successfully linked gene mutations to transcriptional phenotypes through CRISPR screening and single-cell transcriptomics *via* Perturb-seq methods.^1208^ Additionally, the development of the CRISPR-human Organoids-Single-cell RNA Sequencing (CHOOSE) system offers a comprehensive screening approach for functional loss in organoids, thus providing a detectable pathway for precise metabolic regulation of diseases.^1209^ These findings indicate that the combination of CRISPR-Cas9 gene editing technology with single-cell metabolomics and human organoids holds promise for accurately monitoring cellular metabolic changes in relevant diseases. By combining gene editing with mass spectrometry technology, the wide analysis of metabolites in CRISPR-edited cell or animal models to detect changes in metabolic pathways is achievable. The integration of gene editing with spatial genomics enables detailed mapping of the relationships between gene expression patterns and metabolites, including their spatial distributions within tissues and organs.

Therapeutic strategies: 1. Develop multitarget drugs that target multiple metabolic processes, such as glucose metabolism, lipid metabolism, and protein metabolism, while simultaneously regulating multiple key enzymes or signaling pathways. Combining this approach with immunotherapy and dietary therapy, among other methods, can address the inefficiency of current single-target therapies. 2. Address discrepancies between laboratory research and clinical settings: Human pathogenesis and metabolic plasticity are often more complex than animal models. Bridging this gap and focusing on personalized treatments constitute critical aspects for successful clinical translation. 3. Ensuring the safety of gene therapy: Therapies based on CRISPR-Cas9 are on the rise. Future emphasis should focus on improving the targeting specificity and effectiveness of gene therapy to minimize damage to healthy tissues, possibly achieved through enhancing delivery vehicles. 4. Focus on dietary interventions: Approaches such as intermittent fasting and starvation-based therapies have shown initial success in disease treatment. Further clarification of disease dynamics through the aforementioned diagnostics, identification of treatment windows, and tailoring of dietary plans can increase patient compliance and treatment efficacy. 5. Other considerations: The presence of key enzymes as isoenzymes in different tissues and cells underscores the versatility and adaptability of metabolic pathways. Furthermore, mitochondrial transfer is also quite common in diseases. Recent studies suggest that transferring mitochondria from BMSCs to CD8^+^ T cells significantly enhance antitumor efficacy,^1210^ providing a promising therapeutic avenue.

In essence, the regulation of energy metabolism as a therapeutic strategy shows considerable promise amidst challenges and hope. Successfully integrating these findings into clinical practice will necessitate rigorous and comprehensive efforts in both fundamental and translational research.
